# *meso*-Tetrahexyl-7,8-dihydroxychlorin and Its Conversion to ß-Modified Derivatives

**DOI:** 10.3390/molecules29092144

**Published:** 2024-05-05

**Authors:** Daniel Aicher, Dinusha Damunupola, Christian B. W. Stark, Arno Wiehe, Christian Brückner

**Affiliations:** 1 Institut für Chemie und Biochemie, Freie Universität Berlin, Takustr. 3, 14195 Berlin, Germany; 2 Department of Chemistry, University of Connecticut, 55 N Eagleville Rd., Storrs, CT 06269-3060, USA

**Keywords:** *meso*-alkylporphyrins, *meso*-hexylporphyrin, *meso*-hexylchlorin, *meso*-hexylporphyrionoids, porphyrin β-position modification

## Abstract

*meso*-Tetrahexylporphyrin was converted to its corresponding 7,8-dihydroxychlorin using an osmium tetroxide-mediated dihydroxylation strategy. Its diol moiety was shown to be able to undergo a number of subsequent oxidation reactions to form a chlorin dione and porpholactone, the first *meso*-alkylporphyrin-based porphyrinoid containing a non-pyrrolic building block. Further, the diol chlorin was shown to be susceptible to dehydration, forming the porphyrin enol that is in equilibrium with its keto-chlorin form. The *meso*-hexylchlorin dione could be reduced and it underwent mono- and bis-methylation reactions using methyl-Grignard reagents, and trifluoromethylation using the Ruppert-Prakash reagent. The optical and spectroscopic properties of the products are discussed and contrasted to their corresponding *meso*-aryl derivatives (where known). This contribution establishes *meso*-tetrahexyl-7,8-dihydroxychlorins as a new and versatile class of chlorins that is susceptible to a broad range of conversions to generate functionalized chlorins and a pyrrole-modified chlorin analogue.

## 1. Introduction

Hydroporphyrins–chlorins (dihydroporphyrins) and bacteriochlorins (tetrahydroporphyrins) are nature’s principle light harvesting pigments [1]. Synthetic hydroporphyrins and hydroporphyrin analogues have been prepared due to their fundamental importance in the understanding of these photosynthetic pigments [2], for use in bioimaging [3], as markers in fluorescence-guided surgery [4,5,6], as photosensitizers in the photodynamic [3,7] or photothermal [8] therapy of tumors, as photoantimicrobials [9,10], as light-harvesting dyes in dye-sensitized solar cells [11], as catalysts [12], and for many other biological and technical applications [13,14,15,16,17]. The vast majority of synthetic porphyrins (and hydroporphyrins) fall into two classes: the β-alkylporphyrins, modeled closely after nature’s β-alkyltetrapyrroles, and the popular *meso*-arylporphyrins that have no direct precedent in nature.

**Scheme 1:**
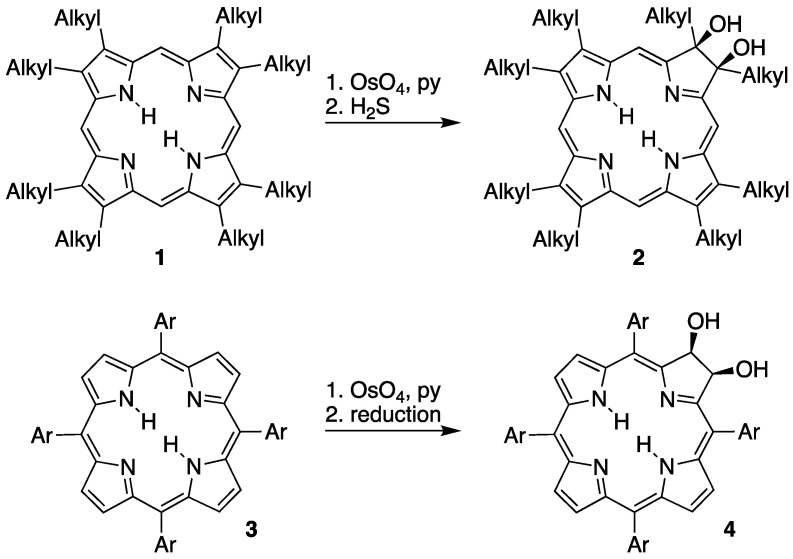
The osmium tetroxide-mediated dihydroxylation of β-alkyl- (**1**) and *meso*-arylporphyrins (**3**) to generate the corresponding dihydroxychlorins **2** and **4**, respectively.

Hydroporphyrins can be prepared by the conversion of porphyrins, by total synthesis, or by the modification of tetrapyrroles derived from nature [13,14,15,16,17]. One well-established method to convert porphyrins to chlorins is through their OsO_4_-mediated dihydroxylation. This versatile method is suitable for converting β-alkylporphyrins **1** or *meso*-arylporphyrins **3** to their corresponding 7,8-dihydroxychlorins **2** and **4**, respectively (Scheme 1) [18,19,20,21,22,23,24]. The regiochemistry and other mechanistic aspects of this reaction are well understood [25,26]. Importantly, the diol functionality in the dihydroxychlorins can be used as a synthetic handle for a number of subsequent functional group transformations, generating a host of chlorins and chlorin analogues [19,20,24,27,28,29,30].

**Figure 1:**
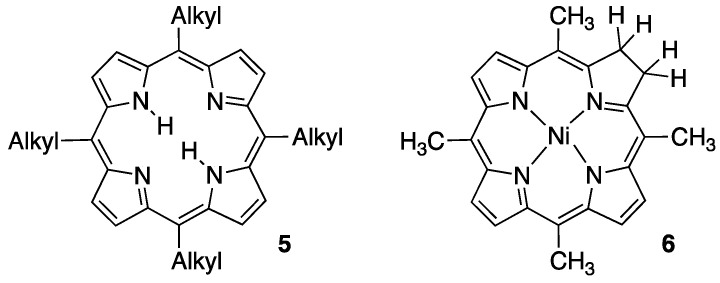
General structure of *meso*-tetraalkylporphyrins **5** and literature-known [*meso*-tetramethylchlorinato]nickel(II) (**6**).

Curiously, the chemistry of *meso*-alkylporphyrins (**5**) (Figure 1), although known for decades [31,32,33], has been far less studied, by a wide margin, compared to that of their β-alkyl- or *meso*-aryl analogues [34,35,36,37,38,39,40,41,42,43,44,45,46,47,48,49,50]. *meso*-Alkyl-porphyrins have also not been, outside of the patent literature [51,52], converted to chlorins. In fact, reports on *meso*-alkylchlorins, in general, are rare: the lone example is *meso*-tetramethylchlorin and its metal complexes, such as *meso*-tetramethylchlorin nickel complex **6** [32,53,54]. The *meso*-tetramethylchlorin metal complexes were formed as by-products during the synthesis of its corresponding metalloporphyrin by means of a 4 × 1-type metal-templated condensation of a suitably derivatized pyrrole [32,53,54].

We recently reported on a neutral tetraalkylporphyrin with high solubility in aqueous solutions [50]. This porphyrin is potentially attractive for biomedical applications. An increase in the intensity of its absorption spectrum in the red region of its optical spectrum and/or a bathochromic shift would much increase its applicability. Typically, the desired optical properties shift is achieved by the conversion of a porphyrin to a chlorin [16,55]. The osmium tetroxide-mediated dihydroxylation of a porphyrin is irreversible, produces a chlorin, and, by virtue of the introduction of the diol functionality, can be expected to increase the amphiphilicity of the molecule, a benefit for its application in photomedicine [43,56,57]. However, neither this dihydroxylation reaction nor the synthetic manipulation of the diol moiety have been, outside of the patent literature [51,52], applied to any *meso*-alkylporphyrins.

This contribution reports the OsO_4_-mediated dihydroxylation and subsequent diol functional group manipulations of an archetype *meso*-tetraalkylporphyrin, *meso*-tetrahexylporphyrin. This work identifies *meso*-alkyl-7,8-dihydroxychlorins as a new class of chlorins that are susceptible to a broad range of conversions to generate functionalized chlorins and a pyrrole-modified chlorin analogue.

## 2. Results and Discussion

### 2.1. The Osmylation of meso-Tetrahexylporphyrin ***7***

**Scheme 2:**
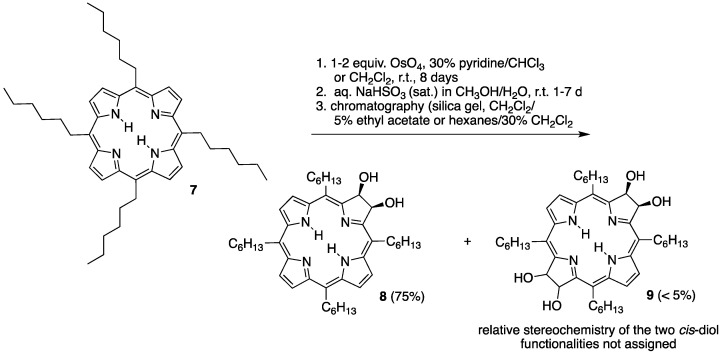
Dihydroxylation of *meso*-tetrahexylporphyrin **7** to produce the corresponding dihydroxychlorin **8** and tetrahydroxybacteriochlorin **9**.

The OsO_4_-mediated dihydroxylation of *meso*-tetrahexylporphyrin **7** took place under standard conditions that are also applicable to octaalkylporphyrins and *meso*-tetraphenylporphyrin: 1–2 equiv OsO_4_ in CHCl_3_/pyridine (optionally applied in two aliquots) over several days at ambient temperature, followed by the reductive cleavage of the initially formed osmate ester (Scheme 2). We found that the osmylation takes place at a comparable, or possibly slightly lower rate, as in *meso*-tetraphenylporphyrin, a finding in line with our expectations for the electron-rich porphyrin **7** [26].

**Figure 2:**
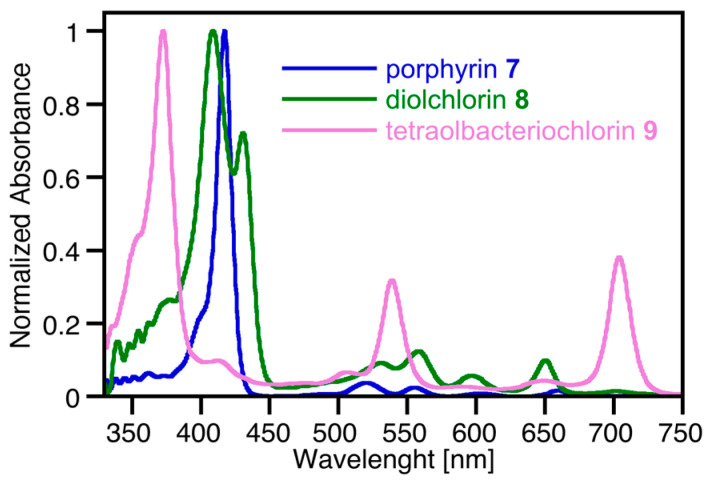
UV–vis spectrum of the compounds indicated (CH_2_Cl_2_).

The success of the dihydroxylation reaction is indicated by the formation of the main polar product **8** with the characteristic optical properties of a chlorin, coupled with a double Soret band (Figure 2). Product **8** can be isolated in satisfactory yields. Its ^1^H NMR spectrum shows a loss of the four-fold symmetry of the starting porphyrin and the formation of a two-fold symmetric product, with diagnostic pyrroline proton signals (at 6.1 ppm); elemental analysis and ESI+ HR mass spectrometry confirmed its expected composition (reproductions of key spectra of all new compounds are provided as Supplementary Material).

Parallel to the dihydroxylation of *meso*-tetraarylporphyrins [58,59], an ‘over-oxidized’ tetrahydroxybacteriochlorin derivative **9** was also observed as a minor side product. A single isomer of bacteriochlorin **9** was spectroscopically characterized, but the relative stereochemistry of its two *cis*-diol functionalities was not assigned.

### 2.2. The Transformations of meso-Tetrahexyl-7,8-dihydroxychlorin ***8***

**Scheme 3:**
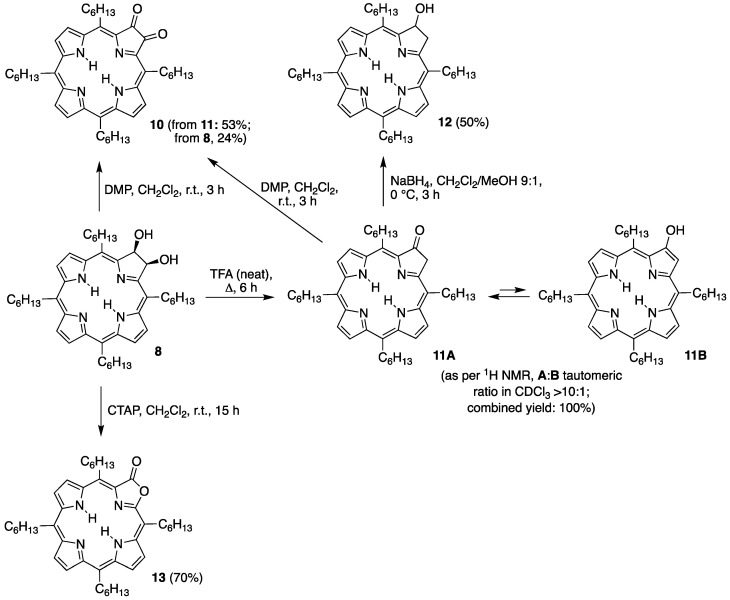
Functional group transformations of *meso*-tetrahexyl-2,3-dihydroxychlorin **8**.

We, and others, have demonstrated the versatility of the diol functionality of octaalkyl- and *meso*-tetraphenylchlorin diols with respect to their functional group transformation, generating a number of porphyrin and chlorin analogues [19,20,27,28,29]. We were now also able to demonstrate the applicability of some of these reactions to *meso*-tetrahexyl-7,8-dihydroxychlorin **8** (Scheme 3).

The oxidation of the *meso*-tetraarylchlorin diols to their corresponding diones using a range of oxidants is well known [28,60]. Using Dess–Martin periodinane (DMP), this transformation is also applicable to chlorin diol **8**, forming *meso*-tetrahexylchlorin dione **10**. The success of the reaction was demonstrated by the loss of the pyrroline hydrogen signals in the ^1^H NMR spectrum of product **10**, its characteristically broadened optical spectrum, and its composition (as per ESI+ HR-MS). The formation of side products and the overall relatively low yield of the dione could be rationalized by the formation of side products resulting from the oxidation of the α-position of its *meso*-alkyl chains (see below). An alternate path to dione **10** was more successful (the conversion of **11** to **10**, see Scheme 3 and below).

The (adventitious) acid-catalyzed or thermally driven dehydration of *meso*-tetraarylchlorin diols [61,62], as well as the related pinacol–pinacolone rearrangement of octaalkychlorin diols [19,27,63], have been previously described. Accordingly, the treatment of chlorin diol **8** with acid induces this dehydration reaction in a satisfying yield, generating ketone-chlorin **11**. An inspection of the ^1^H NMR spectrum of free base **11** (CHCl_3_, 25 °C) shows that the equilibrium position of this keto-enol tautomerism lies, at a minimum, in the ratio of 10:1 on the side of the ketone-chlorin **11A** over its tautomeric form, enol-porphyrin **11B**. This differs from the position of equilibrium for its corresponding *meso*-tetraphenyl derivative; metalation or solvents play a large role in the establishment of this equilibrium [64,65]. Irrespective of the presence of a ketone form in the tautomeric mixture, **11** is inert to reactions with Grignard or alkyl lithium reagents. It is, however, highly susceptible to oxidation with the DMP, smoothly providing dione **10**.

**Figure 3:**
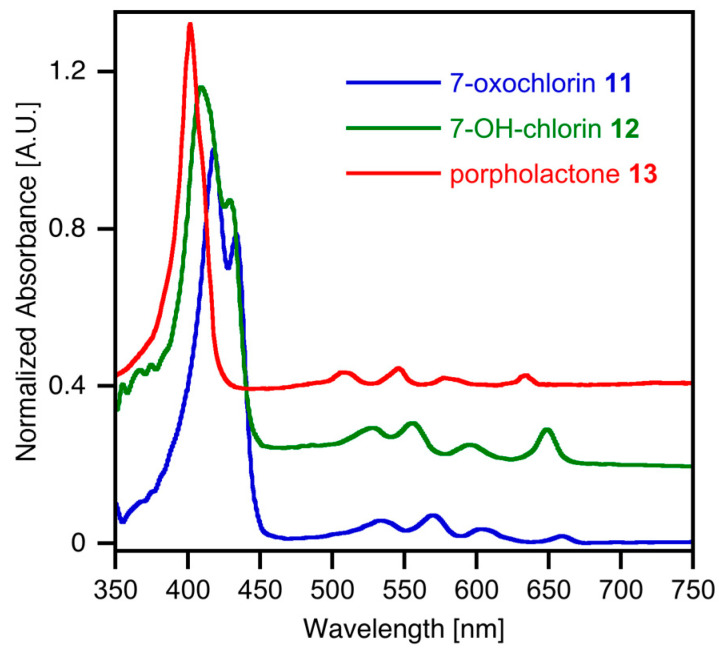
Stacked, normalized UV–vis spectra of the compounds indicated (CH_2_Cl_2_).

The reduction of ketone/enol **11** with NaBH_4_ forms mono-β-hydroxychlorin **12**, characterized by its chlorin-like optical spectrum (Figure 3) and presence of a complex set of peaks assigned to three non-equivalent pyrroline hydrogen atoms (that are, in part, also coupled to the -OH proton). Its corresponding *meso*-tetraphenylchlorin was observed to form as a side product during the hydrogen sulfide reduction of the diol osmate ester [58], or by an alumina-catalyzed (oxygen-mediated) oxidation of its corresponding tetrahydrochlorin [66].

Over the years, we have developed the cetyltrimethylammonium permanganate (CTAP)-induced oxidation of *meso*-tetraarylporphyrins or *meso*-tetraaryldihydroxychlorins to form their corresponding porpholactones [67,68,69,70]. This conversion is complementary to a number of other oxidation reactions that form porpholactones [71,72,73,74,75]. When a *meso*-hexylchlorin diol was reacted with CTAP under standard conditions, the polar diol converted to form non-polar compound **13** with a porphyrin-like optical spectrum (Figure 2). Its ^1^H NMR spectrum indicates the presence of six non-equivalent β-pyrrole protons, and its diagnostic composition (as per ESI+ HRMS) highlights the loss of a carbon atom (and the uptake of two oxygen atoms). Both its FTIR and {^1^H}^13^C NMR spectra show signatures for the presence of a carbonyl functionality (ν_C=O_ at 1842 cm^−1^ and a peak at 168 ppm, respectively). All data support the formation of the target *meso*-hexylporpholactone **13**, the first example of a *meso*-alkylporphyrin analogue containing a non-pyrrolic heterocycle [16,76,77,78].

### 2.3. Direct Oxidations of meso-Tetrahexylporphyrin

**Scheme 4:**
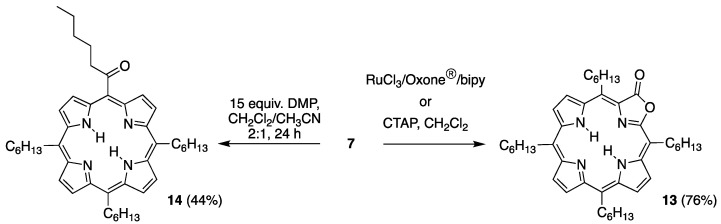
One-step oxidative transformations of *meso*-tetrahexylporphyrin **7**.

In the reactions of diol chlorin **8** or keto-enol chlorin **11** with DMP (Scheme 3), we noticed the formation of several side products with porphyrin-like optical spectra. A slight variation of the reaction solvent used, when applied to *meso*-tetrahexylporphyrin **7**, provided the main product **14** in good yield (Scheme 4). Its composition indicated that a single CH_2_-to-C=O oxidation had taken place. Its ^1^H NMR indicated the presence of all pyrrole hydrogens, but the region corresponding to the most shielded CH_2_ group, the group closest to the ring, had become more complex, suggestive of the formation of three non-equivalent *meso*-hexyl groups, supporting its assignment as the 1’-oxohexyl derivative **14**. Evidently, the porphyrin ring activated the *meso*-hexyl group to allow for an alkane CH oxidation, a reaction not ordinarily observed in DMP-mediated oxidations [79,80].

A number of direct—i.e., not requiring any prior porphyrin functionalization—porphyrin-to-porpholactone conversions are known; chief among them are the RuCl_3_/oxone^®^/bipy oxidation developed by Zhang and co-workers [75,81] and the CTAP reaction referred to above [69,70,82]. Both reactions can be applied to *meso*-tetrakis(pentafluorophenyl)porphyrins, but the CTAP oxidation is ineffective for the oxidation of *meso*-tetraphenylporphyrins [70]. We were thus surprised to find that both RuCl_3_/oxone^®^/bipy oxidation and CTAP-mediated oxidation are applicable to the oxidation of free base *meso*-tetrahexylporphyrin **7** to generate the target *meso*-tetrahexylporpholactone **13**. In fact, CTAP-mediated oxidation is highly efficient and rapid (5 min), generating porpholactone **13** in a high yield and with only a modicum of side products, even outperforming Ru-based oxidation (or a two-step oxidation via chlorin diol **8**, Scheme 3).

**Scheme 5:**
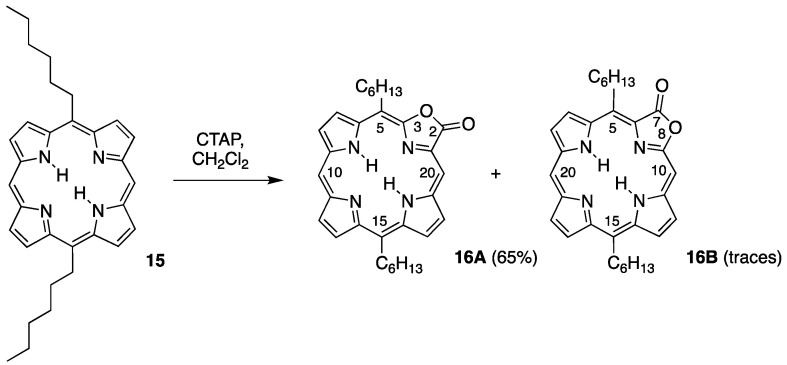
Direct CTAP oxidation of *meso*-5,15-dihexylporphyrin **15**.

The direct (and clean) porphyrin-to-porpholactone conversion achieved using CTAP is also possible for the previously reported 5,15-dihexylporphyrin **15** [41,45] (Scheme 5). Here, two compounds of near-identical UV–vis spectra and identical compositions (as per ESI+ HRMS), but with very differing quantities, are formed. The major compound **16A** could be fully spectroscopically characterized and its ^1^H NMR spectrum, for example, supports its assignment as the target dihexylporpholactone, but the second compound **16B** was only formed in insufficient quantities to fully characterize it by NMR spectroscopy. Nonetheless, based on the identical UV–vis and mass spectra of the two compounds, we assigned them to be the two possible lactone regioisomers **16A** and **16B**.

**Figure 4:**
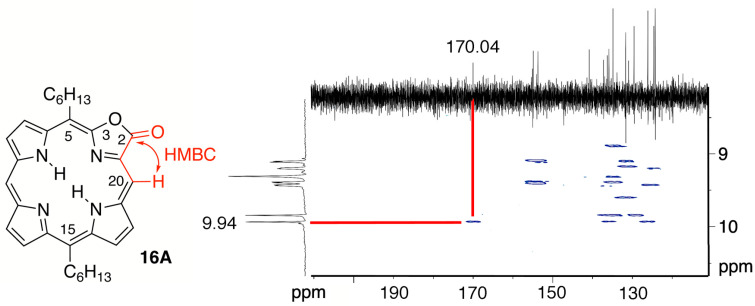
Illustrated partial ^1^H,^13^C-HMBC NMR spectrum observed for dihexylporpholactone isomer **16A**.

The assignment of these specific regioisomers can be accomplished using the heteronuclear three-bond correlation HMBC (^3^*J*_C,H_) spectrum of the major fraction, isomer **16A** (Figure 4): In the {^1^H}^13^C NMR spectrum of **16A**, the most down-field-shifted quaternary carbon signal at 168 ppm can be assigned to the carbonyl carbon atom; likewise, the two most down-field-shifted signals in its ^1^H NMR spectrum (s at 9.94 and 9.84 ppm) stand out and can be clearly assigned to the two non-equivalent *meso*-hydrogen atoms. A clear three-bond interaction between the carbonyl carbon of the lactone moiety and one of the *meso*-protons (at 9.94 ppm) can be seen in the HMBC spectrum of **16A**, unequivocally identifying this porpholactone as the regioisomer **16A,** shown carrying its carbonyl group on the side of the (less sterically encumbered) *meso*-position; the other isomer **16B** would not be expected to show any ^3^*J*_C,H_ coupling between the carbonyl carbon atom and any *meso*-hydrogen atom. The sterically less encumbered orientation of the lactone moiety was also the prevalent orientation found in 5,15-diphenylporpholactones [68].

### 2.4. The Transformations of meso-Tetrahexylchlorin-7,8-dione ***10***

**Scheme 6:**
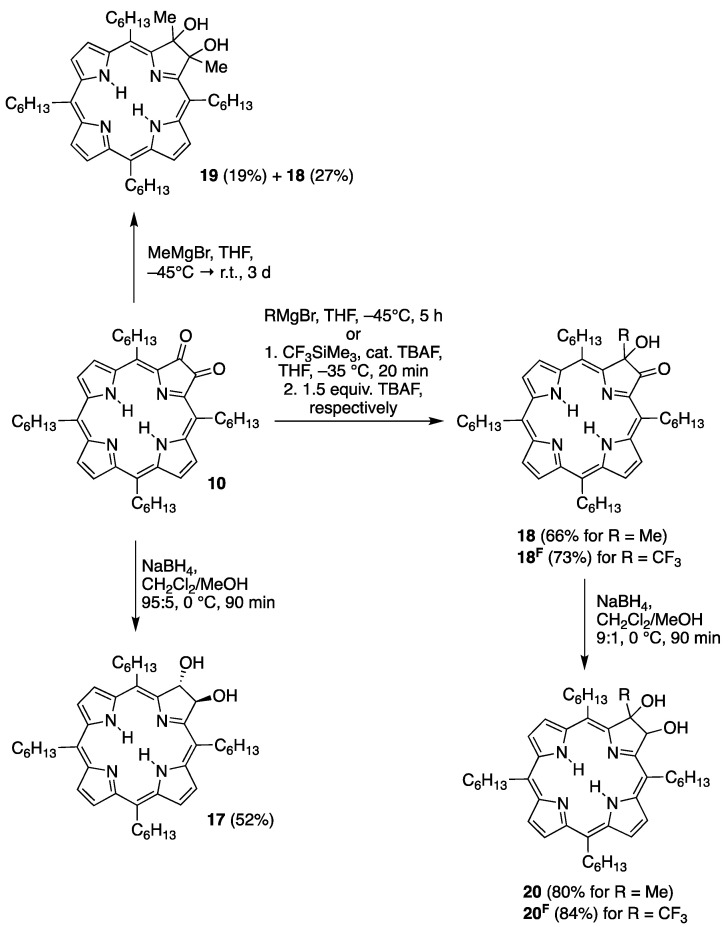
Transformations of *meso*-tetrahexylporphyrin-7,8-dione **10**.

The carbonyl-type reactivity of the ketone groups in *meso*-tetraarylchlorin-7,8-diones has been amply demonstrated [30,71,83,84]. We were able to confirm this also for *meso*-alkyldione **10**. Correspondingly, dione **10** could be reduced with NaBH_4_ to its corresponding diol **17** possessing a UV–vis spectrum that is near-identical to that of diol **9**. Both compounds possess the same composition (as per HR-MS). We suggest that diol **17** is the *trans*-diol isomer of the *cis*-isomer **9** (Scheme 6). The ^1^H NMR spectrum of the 2-fold rotationally symmetric *trans*-isomer **17** and its mirror-symmetric *cis*-isomer **9** vary slightly, most significantly with respect to a 0.5 ppm difference in the shift of the pyrroline proton; their R_f_-values differ also, with diol **17** being less polar than diol **9**.

**Figure 5:**
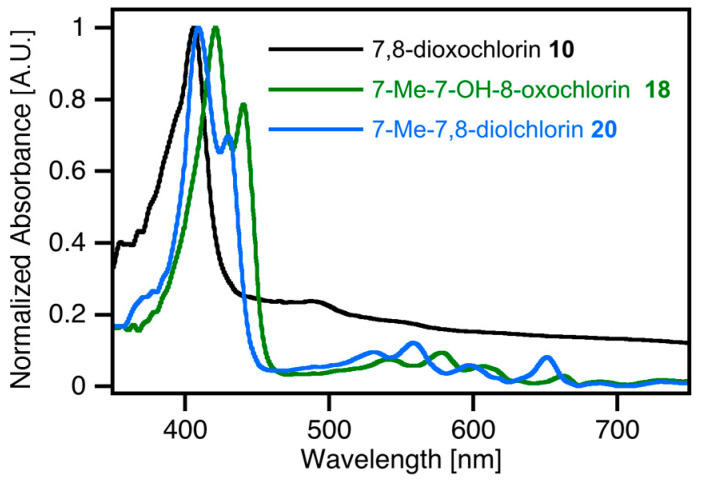
UV–vis spectra of the compounds indicated (CH_2_Cl_2_).

Dione **10** is also susceptible to single and double methyl-Grignard addition, forming α-hydroxyketone **18** in satisfying yields and (*trans*) diol **19** in moderate yields, even under more forcing conditions. This reaction has precedent in the *meso*-arylporphyrin series [85]. α-Hydroxyketone **18** can be reduced with NaBH_4_ to the corresponding (likely *trans*) diol **20**. Dione **10** also undergoes nucleophilic CF_3_-group addition using the Ruppert-Prakash reagent, CF_3_SiMe/TBAF [86,87], yielding product **18^F^**, without showing the formation of a bis-adduct. Like α-hydroxyketone **18**, the ketone moiety in its trifluoromethyl analogue **18^F^** can also be reduced to form the corresponding chlorin diol **20F**. All novel compounds had the expected analytical and spectroscopic properties. All diols (**17**, **19**, **20**, and **20F**) show very similar chlorin-like UV–vis spectra (see, e.g., Figure 5 and ESI); the strong electronic influence of the β-ketone is clearly visible. These compounds add to the series of hydroporphyrins with a redox state between that of the dione and the diol chlorin, some of which were explored in the *meso*-aryl series as well [88].

In summary, using *meso*-tetrahexylporphyrin **7** as a representative of the much under-studied *meso*-alkylporphyrins, we have shown that it is readily converted to a range of chlorins and porpholactone using multiple reactions, most of which had precedents in the chemistry of the better-investigated *meso*-aryl- and β-alkylporphyrins. The key steps are the smooth OsO_4_-mediated dihydroxylation of the porphyrin to its corresponding dihydroxychlorin, which functionalizes the porphyrin toward further transformations. Other direct oxidative conversions of the porphyrin and of the *meso*-hexyl group could also be demonstrated, with some reaction features unique to the *meso*-alkylporphyrin/chlorin. Thus, this contribution establishes *meso*-tetrahexyl-7,8-dihydroxychlorins as a new and versatile class of chlorins that is susceptible to a broad range of conversions to generate functionalized chlorins and pyrrole-modified chlorin analogues. The *meso*-alkylporphyrin and -chlorins are readily derived, soluble, and stable, ideal prerequisites for further work on this class of compounds.

## 3. Materials and Methods

### 3.1. Materials

Aluminum-backed, silica gel 60, 250 μm thick analytical plates were used for analytical TLC; either 20 × 20 cm, glass-backed, silica gel 60, 500 µm thick preparative TLC plates or standard grade, 60 Å, 32–63 µm flash column silica gel were used for the chromatographic separation and purification of the products.

All chemicals and solvents were used as received or purified/dried according to standard procedures [89]. Cetyltrimethylammonium permanganate (CTAP) [90], *meso*-tetrahexylporphyrin **7** [34,46], and 5,15-dihexylporphyrin **15** [41,43] were prepared as described in the literature.

### 3.2. Instruments

^1^H and {^1^H}^13^C NMR spectra were recorded on Bruker instruments in the solvents indicated and were referenced to residual solvent peaks or internal TMS. Where present, structural assignments were performed with the help of COSY (^3^*J*_H,H_), HMQC (^1^*J*_C,H_), and HMBC (^2^*J*_C,H_ and ^3^ J_C,H_) spectra and NOE experiments. UV–vis spectra were recorded either on Cary 50, 60, or 100 (Varian, Palo Alto, CA, USA, now Agilent, Santa Clara, CA, USA) or Specord S300 UV-vis (Analytik Jena, Jena, Germany) spectrometers in 1 cm glass or quartz cells, in the solvents indicated. Fluorescence emission spectra were recorded on a Cary Eclipse spectrometer in 1 cm glass or quartz cells, in the solvent indicated. FT-IR spectra were recorded on an Alpha (Bruker, Billerica, MA, USA) instrument (diamond ATR). Mass spectrometry analyses were performed on a QStar Elite (AB Sciex, Framingham, MA, USA) Quadrupole-TOF, Agilent 6210 ESI-TOF (Agilent, Santa Clara, CA, USA), or Ionspec QFT-7 ESI-FTICR (Varian Inc., Lake Forest, CA, USA) mass spectrometer.

### 3.3. General Procedures

#### 3.3.1. General Procedure A: Hydride Reduction

The starting porphyrin is dissolved in CH_2_Cl_2_/MeOH and, at 0 °C, 5–10 equivalents of NaBH_4_ are added (in portions). Stirring continued until the TLC control indicated the consumption of the starting material. Water was added to the reaction mixture and the organic phase was separated in a separatory funnel. If the aqueous phase was colored, it was extracted with CH_2_Cl_2_ or ethyl acetate. The combined organic phases were washed with water, dried over Na_2_SO_4_ (anhyd), and the solvent removed by rotary evaporation. The residue was chromatographed and the fractions recrystallized.

#### 3.3.2. General Procedure B: CTAP Oxidation

The starting porphyrin or chlorin was, in a round-bottom flask equipped with a stir bar, dissolved in CH_2_Cl_2_, and freshly prepared CTAP was added and stirred at ambient conditions. The reaction’s progress was observed by TLC; once the desired conversion had taken place, the solvent was reduced by rotary evaporation, the concentrate passed through a pad of Celite^®^, and the pad was washed with CH_2_Cl_2_ until the eluent was largely colorless. The combined filtrates were evaporated to dryness by rotary evaporation and the crude mixture was chromatographed and the fractions recrystallized.

#### 3.3.3. General Procedure C: DMP Oxidation

The starting porphyrin was dissolved in CH_2_Cl_2_ and a solution of 5–6 equivalents of Dess–Martin periodinane (DMP) dissolved in CH_2_Cl_2_ was added drop-wise in ambient conditions until the TLC control indicated the consumption of the starting material. Water was added to the reaction mixture and the organic phase was separated in a separatory funnel. If the aqueous phase was colored, it was extracted with CH_2_Cl_2_ or ethyl acetate. The combined organic phases were washed with water, dried over Na_2_SO_4_ (anhyd), and the solvent removed by rotary evaporation. The residue was chromatographed and the fractions recrystallized.

### 3.4. Osmium Tetroxide-Mediated Dihydroxylation of meso-Tetrahexylporphyrin (***7***): Formation of meso-Tetrahexyl-7,8-cis-dihydroxychlorin (***8***) and meso-Tetrahexyl-7,8,7,18-cis-tetrahydroxybacteriochlorin (***9***)

In a 50 mL round-bottom flask, *meso*-tetrahexylporphyrin (**7**) (148 mg, 0.2 mmol, 1 equiv.) was dissolved in a mixture of CHCl_3_ (15 mL, EtOH-stabilized) and freshly distilled pyridine (2 mL). A solution of OsO_4_ (2.9 mL of an OsO_4_ stock solution of 1.0 g OsO_4_, 3.93 mmol, in 25 mL of 30% pyridine/CHCl_3_, amounting to 0.46 mmol, 2.3 equiv.) was added to the mixture. [CAUTION: note the hazard and risk of using OsO_4_; the use of a fume hood and suitable PPE—nitrile gloves, safety goggles, and a lab coat—are required.] The flask was stoppered, shielded from light with aluminum foil, and magnetically stirred at ambient temperature for ~7 days. The progress of the reaction was monitored by occasional TLC for the consumption of the starting material. Once no further progress was noted, the solvent was removed to dryness on a rotary evaporator at the lowest temperature feasible. The crude osmate ester product was then dissolved in a solution of 10% MeOH/CHCl_3_ (~15 mL) and vigorously stirred with a sat. aqueous (or 1:1 MeOH/H_2_O) NaHSO_3_ solution (~20 mL) for up to 7 days (monitored by TLC). Once all the intermediate osmate ester was consumed, the mixture was extracted with CHCl_3_ twice, and its organic fraction was isolated and dried over Na_2_SO_4_ anhyd. The drying agent was removed by filtration and the filtrate was evaporated to dryness by rotary evaporation. The resulting residue was dissolved in a minimal amount of CH_2_Cl_2_ and loaded onto a silica gel column and eluted with CH_2_Cl_2_. The first fraction, eluted with 90% hexanes/CH_2_Cl_2_, was starting material **7** (~5%). Target chlorin diol **8** was eluted with 30% hexanes/CH_2_Cl_2_. Alternatively, silica gel column chromatography using 95% CH_2_Cl_2_/5% ethyl acetate is suitable. Slow evaporation from a hexanes/CH_2_Cl_2_ mixture (or recrystallization from CH_2_Cl_2_:MeOH) provided product **8** as a purple fluffy solid (101 mg, 75%). A second, more polar light pink fraction was identified as tetrahydroxybacteriochlorin **9**.

**8**: R_f_ (silica–20% hexanes/CH_2_Cl_2_) = 0.69; R_f_ (silica–95% CH_2_Cl_2_/5% ethyl acetate) = 0.85. ^1^H NMR (400 MHz; CDCl_3_): δ −1.87–2.11 (br s, 1H), 0.92–0.89 (m, 6H), 1.55–1.37 (m, 8H), 1.70 (ddq, *J* = 36.0, 14.5, 7.2 Hz, 4H), 2.10–1.99 (m, 2H), 2.38 (dq, *J* = 14.4, 7.4 Hz, 2H), 2.76–2.58 (m, 1H), 4.11 (t, *J* = 8.0 Hz, 2H), 4.56–4.40 (m, 2H), 6.11 (s, 1H), 8.73 (d, *J* = 4.9 Hz, 1H), 8.95 (d, *J* = 4.8 Hz, 1H), 9.12 (s, 1H) ppm. {^1^H}^13^C NMR (126 MHz, CDCl_3_): δ 159.7, 152.0, 139.5, 133.9 (*α*-C), 129.6 (C-17, C-18), 124.7, 121.4 (C-2, C-3, C-12, C-13), 110.8 (*meso*-C), 73.0 (C-7, C-8), 38.1 (C-32), 36.2 (C-26), 35.0 (C-31), 32.9 (C-25), 31.9, 31.9 (C-28, C-34), 30.4, 30.2 (C-27, C-34), 22.9, 22.9 (C-29, C-35), 14.3, 14.3 (C-30, C-36) ppm. UV–vis (CH_2_Cl_2_) λ_max_ (log ε): 402 (5.31), 423 (sh), 522 (3.07), 550 (3.12), 588 (3.45), 640 (2.39) nm. Fl (λ_excitation_ = λ_Soret_) λ_max_ (rel. intensity) = 652 (1.0), 720 (0.09) nm. MS (EI, 170 °C): *m*/*z* = 680 (39%, [M]^+^), 662 (100%, [M − H_2_O]^+^), 646 (21%, [M–2 OH]^+^), 609 (21%, [M–C_5_H_11_]^+^), 591 (45%, [M − H_2_O–C_5_H_11_]^+^). HR-MS (ESI+, 100% CH_3_CN, 30 V cone voltage, TOF detection): *m*/*z* calc’d for C_44_H_65_N_4_O_2_ [M + H]^+^, 681.5108; found 681.5134.

**9**: ^1^H NMR (500 MHz, DMSO-d_6_): δ = –1.83 (s, 2H, NH), 0.92 (t, *J* = 7.3 Hz, 12 H, 4 × CH_3_), 1.33–1.40 (m, 8 H, 4 × CH_2_), 1.42–1.48 (m, 8 H, 4 × CH_2_), 1.68 (m, 8 H, 4 × CH_2_), 2.04–2.18 (m, 8 H, 4 × CH_2_), 4.15–4.21 (m, 4 H, 2 × CH_2_), 4.35–4.42 (m, 4 H, 2 × CH_2_), 5.72–5.76 (m, 4 H, -OH), 6.22–6.25 (m, 4 H, pyrroline-H), 8.92–8.94 (m, 4 H, pyrrole-H) ppm. {^1^H}^13^C NMR (126 MHz, DMSO-d_6_): δ = 14.05 (CH_3_), 22.27 (CH_2_), 29.69 (CH_2_), 30.69 (CH_2_), 31.38 (CH_2_), 32.61 (CH_2_), 35.52 (CH_2_), 72.56 (pyrroline-C), 113.42 (*meso*-C), 120.47 (β-C), 134.97 (α-C), 158.82 (α-C) ppm. UV–vis (CH_2_Cl_2_): λ_max_ (log ε): 373 (5.26), 413 (4.26), 507 (3.92), 539 (4.68), 650 (3.77), 704 (4.77) nm. HRMS (ESI, TOF detection): *m*/*z* calc’d. for C_44_H_67_N_4_O_4_^+^ ([M + H]^+^): 715.5157; found 715.5153.

### 3.5. DMP Oxidation of meso-Tetrahexyl-7,8-cis-dihydroxychlorin (***8***) or meso-Tetrahexylchlorin-7-one (***11***): Formation of meso-Tetrahexylporphyrin-7,8-dione (***10***)

According to General Procedure C, *meso*-tetrahexyl-7,8-*cis*-dihydroxychlorin (**8**) or *meso*-tetrahexylchlorin-7-one (**11**) (250 mg, 0.37 mmol), in CH_2_Cl_2_ (15 mL), was reacted with a 15% (*w*/*w*) solution of Dess–Martin periodinane (DMP) in CH_2_Cl_2_ (1.6 g, 1.9 mmol) over 3 h in ambient conditions. Column chromatography on silica–2:1 CH_2_Cl_2_/hexane. Recrystallization from CH_2_Cl_2_/MeOH provided the dione **10** as a purple solid in 53% yield (133 mg).

**10**: ^1^H NMR (500 MHz, CDCl_3_): δ –2.60 (s, 2 H, NH), 0.94–0.97 (m, 12 H, 4 × CH_3_), 1.36–1.53 (m, 16 H, 8 × CH_2_), 1.69–1.80 (m, 8 H, 4 × CH_2_), 1.95–2.03 (m, 4 H, 2 × CH_2_), 2.34–2.41 (m, 4 H, 2 × CH_2_), 4.35–4.41 (m, 4 H, 2 × CH_2_), 4.58–4.63 (m, 4 H, 2 × CH_2_), 9.00–9.02 (m, 2 H, β-H), 9.11–9.13 (m, 4 H, β-H) ppm. {^1^H}^13^C NMR (126 MHz, CDCl_3_): δ = 14.30 (CH_3_), 14.34 (CH_3_), 22.89 (CH_2_), 22.94 (CH_2_), 30.22 (CH_2_), 30.41 (CH_2_), 31.15 (CH_2_), 31.97 (CH_2_), 32.01 (CH_2_), 35.56 (CH_2_), 36.44 (CH_2_), 38.35 (CH_2_), 114.35 (*meso*-C), 122.80 (*meso*-C), 124.76 (β-C), 125.28 (β-C), 131.66 (β-C), 136.78 (α-C), 138.47 (α-C), 139.48 (α-C), 154.41 (α-C), 189.22 (β-CO) ppm. UV–vis (CH_2_Cl_2_): λ_max_ (log ε): 406 (5.31), 491 (4.23), 715 (3.72) nm. HRMS (ESI+, TOF detection): *m*/*z* calc’d for C_44_H_61_N_4_O_2_^+^ ([M + H]^+^): 677.4789; found: 677.4793. HRMS (ESI^−^, TOF detection): *m*/*z* calc’d for C_44_H_59_N_4_O_2_^−^ ([M − H]^−^): 675.4644; found: 675.4621.

### 3.6. Dehydration of meso-Tetrahexyl-7,8-cis-dihydroxychlorin (***8***): Formation of meso-Tetrahexylchlorin-7-one (***11***)

Dihydroxychlorin **8** (400 mg, 0.59 mmol) was dissolved in TFA (30 mL) and heated to 65 °C [CAUTION: Trifluoroacetic acid is a strongly corrosive acid that poses an inhalation hazard. The use of a fume hood and suitable PPE—nitrile gloves, safety goggles, and a lab coat—are required]. After 8 h, the mixture was added to ice water in a separatory funnel and a 20% aqueous solution of NaOH was added until neutrality was reached. The product is extracted with ethyl acetate (2×), the combined organic phases were washed with water, dried over Na_2_SO_4_ (anhyd.), and the solvent removed by rotary evaporation. Recrystallization of the residue from CH_2_Cl_2_/MeOH delivered the final product **11** as a purple solid in 96% yield (375 mg).

**11**: ^1^H NMR (500 MHz, CDCl_3_): (keto tautomer, 10:1 dominant over the enol tautomer) δ −2.49 (s, 1 H, NH), −2.26 (s, 1 H, NH), 0.94–0.99 (m, 12 H, 4 × CH_3_), 1.35–1.55 (m, 16 H, 8 × CH_2_), 1.61–1.67 (m, 2 H, CH_2_), 1.73–1.83 (m, 6 H, 3 ×CH_2_), 2.00–2.14 (m, 4 H, 2 × CH_2_), 2.37–2.48 (m, 4 H, 2 × CH_2_), 3.77–3.81 (m, 2 H, CH_2_), 4.51 (s, 2 H, β-H), 4.62–4.72 (m, 6 H, 3 × CH_2_), 8.84 (d, *J* = 4.8 Hz, 1 H, β-H), 9.13 (d, *J* = 4.7 Hz, 1 H, β-H), 9.16 (d, *J* = 4.8 Hz, 1 H, β-H), 9.19 (d, *J* = 4.6 Hz, 1 H, β-H), 9.22 (d, *J* = 4.5 Hz, 1 H, β-H), 9.25 (d, *J* = 4.6 Hz, 1 H, β-H) ppm. {^1^H}^13^C NMR (126 MHz, DMSO-d_6_): δ =14.25 (CH_3_), 14.27 (CH_3_), 14.33 (CH_3_), 22.86 (CH_2_), 22.96 (CH_2_), 30.17 (CH_2_), 30.34 (CH_2_), 30.44 (CH_2_), 30.81 (CH_2_), 31.88 (CH_2_), 32.00 (CH_2_), 32.06 (CH_2_), 35.04 (CH_2_), 35.18 (CH_2_), 35.72 (CH_2_), 35.78 (CH_2_), 36.09 (CH_2_), 38.22 (CH_2_), 38.39 (CH_2_), 46.40 (β-C), 109.56 (*meso*-C), 116.00 (*meso*-C), 119.46 (*meso*-C), 122.86 (β-C), 123.12 (*meso*-C), 123.65 (β-C), 124.41 (β-C), 125.64 (β-C), 130.26 (β-C), 131.34 (β-C), 135.30 (α-C), 136.41 (α-C), 138.36 (α-C), 138.45 (α-C), 145.96 (α-C), 152.36 (α-C), 154.57 (α-C), 155.13 (α-C), 205.62 (β-CO) ppm. UV–vis (CH_2_Cl_2_): λ_max_ (log ε): 419 (5.39), 433 (5.29), 534 (4.20), 570 (4.29), 604 (4.00), 659 (3.76) nm. HRMS (ESI+, TOF detection): *m*/*z* calc’d for C_44_H_63_N_4_O^+^ ([M + H]^+^): 663.4996; found: 663.4937.

### 3.7. Hydride Reduction of meso-Tetrahexylchlorin-7-one (***11***): Formation of meso-Tetrahexyl-7-hydroxychlorin (***12***)

According to General Procedure A, chlorin **11** (70 mg, 0.11 mmol) was reacted with NaBH_4_ (28 mg, 0.74 mmol) in 9:1 CH_2_Cl_2_/MeOH (3 mL) over 3 h in ambient conditions. Column chromatography (silica–3:1 CH_2_Cl_2_/hexane, then 99:1 CH_2_Cl_2_/ethyl acetate), followed by recrystallization of the main fraction (from CH_2_Cl_2_/MeOH), provided chlorin **12** as a violet solid in 50% yield (35 mg).

**12**: ^1^H NMR (500 MHz, CDCl_3_): δ = –1.81 (s, 1 H, NH), –1.78 (s, 1 H, NH), 0.93–1.00 (m, 12 H, 4 × CH_3_), 1.37–1.55 (m, 16 H, 8 × CH_2_), 1.70–1.83 (m, 8 H, 4 × CH_2_), 2.02–2.07 (m, 1 H, β-OH), 2.14–2.22 (m, 2 H, CH_2_), 2.24–2.32 (m, 2 H, CH_2_), 2.39–2.48 (m, 4 H, 2 × CH_2_), 4.03–4.20 (m, 2 H, CH_2_), 4.28–4.39 (m, 2 H, CH_2_), 4.40–4.52 (m, 2 H, β-H), 4.67–4.76 (m, 4 H, 2 × CH_2_), 6.58–6.63 (m, 1 H, β-H), 8.95 (d, *J* = 5.0 Hz, 1 H, β-H), 8.99 (d, *J* = 4.8 Hz, 1 H, β-H), 9.16 (d, *J* = 4.5 Hz, 1 H, β-H), 9.19 (d, *J* = 4.5 Hz, 1 H, β-H), 9.25–9.28 (m, 2 H, β-H), ppm. {^1^H}^13^C NMR (126 MHz, CDCl_3_): δ = 14.35 (CH_3_), 22.92 (CH_2_), 22.98 (CH_2_), 30.33 (CH_2_), 30.43 (CH_2_), 30.47 (CH_2_), 30.52 (CH_2_), 32.07 (CH_2_), 32.09 (CH_2_), 34.12 (CH_2_), 35.01 (CH_2_), 35.23 (CH_2_), 35.43 (CH_2_), 35.57 (CH_2_), 37.20 (CH_2_), 38.18 (CH_2_), 38.23 (CH_2_), 44.50 (β-C-8), 73.24 (β-C-7), 109.93 (*meso*-C), 110.95 (*meso*-C), 120.80 (β-C), 121.06 (β-C), 121.77 (*meso*-C), 122.24 (*meso*-C), 124.90 (β-C), 125.53 (β-C), 129.58 (β-C), 130.02 (β-C), 134.21 (α-C), 134.81 (α-C), 139.71 (α-C), 139.82 (α-C), 151.81 (α-C), 152.71 (α-C), 161.44 (α-C), 162.69 (α-C) ppm. UV–vis (CH_2_Cl_2_): λ_max_ (log ε): 410 (5.37), 430 (5.22), 529 (4.36), 556 (4.42), 596 (4.09), 649 (4.37) nm. HRMS (ESI): *m*/*z* calc’d for C_44_H_65_N_4_O^+^ ([M + H]^+^): 665.5153; found: 665.5156.

### 3.8. CTAP Oxidation of meso-Tetrahexylporphyrin (***7***) or meso-Tetrahexyl-7,8-cis-dihydroxychlorin (***8***): Formation of meso-Tetrahexylporpholactone (meso-Tetrahexyl-7-oxo-8-oxa-porphyrin) (***13***)

Prepared according to General Procedure B from porphyrin **7** or chlorin diol **8** (50 mg, 0.08 mmol, 1 equiv.) in CH_2_Cl_2_ (12 mL) and CTAP (0.375 mmol, 152 mg, 5 equiv.) over a 10–30 min reaction time. The crude mixture was chromatographed (silica–10% CH_2_Cl_2_/hexanes), resulting in a 76% yield of **13** (254 mg). R_f_ (silica–30% CH_2_Cl_2_/hexanes) = 0.54. ^1^H NMR (400 MHz, CD_2_Cl_2_): δ = 9.38 (s, 2 H), 6.38 (m, 2 H), 5.73-–5.66 (m, 4 H), 3.83 (d, *J* = 7.6 Hz, 1 H), 3.71 (m, 1 H), 3.65 (dt, *J* = 17.5, 8.9 Hz, 1 H), 3.65 (m, 4 H), 2.94 (dt, *J* = 17.3, 8.9 Hz, 1 H) ppm. {^1^H}^13^C NMR (101 MHz, CD_2_Cl_2_): δ = 117.1, 107.8.6, 107.4, 106.4, 78.5, 70.1, 67.2, 66.4, 40.5 ppm. UV–vis (CH_2_Cl_2_) λ_max_ = 402 (Soret), 519, 547, 592, 634 nm. Fl (λ_excitation_ = λ_Soret_) λ_max_ (rel. intensity) = 635 (1.0), 704 (0.17) nm. FT-IR spectrum (neat, ATR): ν_C=O_ = 1842 cm^–1^. HR-MS ESI+ (100% CH_3_CN, 30 V cone voltage): *m*/*z* = 664.4716 calc’d for C_43_H_60_N_4_O_2_ [M]^+^; found 664.4704.

### 3.9. DMP Oxidation of meso-Tetrahexylporphyrin (***7***): Formation of 5-(1′-oxo-hexyl)-10,15,20-trihexylporphyrin (***14***)

According to General Procedure C, porphyrin **7** (150 mg, 0.23 mmol) was reacted in 2:1 CH_2_Cl_2_/CH_3_CN (14 mL) with a 15% (*w*/*w*) solution of Dess–Martin periodinane (DMP) in CH_2_Cl_2_ (3.0 g, 3.6 mmol) over 12 h in ambient conditions. Column chromatography on silica–2:1 CH_2_Cl_2_/hexane. Recrystallization from CH_2_Cl_2_/MeOH provided product **14** as a purple solid in 44% yield (67 mg).

**14**: ^1^H NMR (500 MHz, CDCl_3_): δ = –2.72 (s, 2 H, NH), 0.93–0.97 (m, 12 H, 4 × CH_3_), 1.36–1.59 (m, 16 H, 8 × CH_2_), 1.76–1.85 (m, 6 H, 3 × CH_2_), 2.14 (m, 2 H, CH_2_), 2.44–2.56 (m, 6 H, 3 × CH_2_), 3.72 (t, *J* = 7.6 Hz, 2 H, CH_2_), 4.88 (t, *J* = 8.1 Hz, 4 H, 2 × CH_2_), 4.94 (t, *J* = 8.2 Hz, 2 H, CH_2_), 9.10 (d, *J* = 4.8 Hz, 2 H, β-H), 9.44 (d, *J* = 4.9 Hz, 2 H, β-H), 9.46 (d, *J* = 4.8 Hz, 2 H, β-H), 9.49 (d, *J* = 4.9 Hz, 2 H, β-H) ppm. {^1^H}^13^C NMR (126 MHz, CDCl_3_): δ = 14.14 (CH_3_), 14.30 (CH_3_), 14.31 (CH_3_), 22.76 (CH_2_), 22.89 (CH_2_), 22.91 (CH_2_), 25.45 (CH_2_), 30.39 (CH_2_), 30.48 (CH_2_), 31.81 (CH_2_), 32.05 (CH_2_), 32.06 (CH_2_), 35.51 (CH_2_), 36.10 (CH_2_), 38.83 (CH_2_), 39.10 (CH_2_), 50.79 (COCH_2_), 117.12 (*meso*-C), 120.05 (*meso*-C), 121.53 (*meso*-C), 210.22 (CO) ppm. UV–vis (CH_2_Cl_2_): _λmax_ (log ε): 411 (5.52), 519 (4.19), 553 (3.86), 597 (3.65), 654 (3.68) nm. HRMS (ESI): *m*/*z* calc’d for C_44_H_61_N_4_O^+^ ([M + H]^+^): 661.4840; found: 661.4837.

### 3.10. CTAP Oxidation of 5,15-Dihexylporphyrin (***15***): Formation of 5,15-Dihexyl-3-oxo-2-oxa-porphyrin) (***16A***) and 5,15-Dihexyl-7-oxo-8-oxa-porphyrin) (***16B***)

Prepared according to General Procedure B from porphyrin **15** (50 mg, 0.1 mmol) in CH_2_Cl_2_ (12 mL) and CTAP (0.5 mmol, 202 mg, 5 equiv.) over a 5–10 min reaction time. The filtered reaction mixture was separated by preparative thin-layer chromatography (silica–40% CH_2_Cl_2_/hexanes) to obtain **16A** in 55% yield (29 mg) **16B** in less than 5% yield (<3 mg).

**16A**: R_f_ (silica–40% CH_2_Cl_2_/hexanes) = 0.67. ^1^H NMR (400 MHz, CD_2_Cl_2_): δ = –2.93 (s, 1 H), –2.23 (s, 1 H), 0.96 (t, *J* = 8.2 Hz, 6 H), 1.52–1.39 (m, 8 H), 1.74 (dd, *J* = 15.2, 7.7 Hz, 2 H), 1.82 (t, *J* = 7.5 Hz, 2 H), 2.34 (dq, *J* = 15.4, 7.7 Hz, 3 H), 2.50 (dt, *J* = 15.5, 7.8 Hz, 2 H), 4.52 (t, *J* = 7.9 Hz, 2 H), 4.87 (t, *J* = 8.0 Hz, 2 H), 9.10 (d, *J* = 4.4 Hz, 1 H), 9.19 (d, *J* = 3.4 Hz, 1 H), 9.30 (s, 2 H), 9.39 (d, *J* = 4.4 Hz, 1 H), 9.43 (d, *J* = 4.4 Hz, 1 H), 9.84 (s, 1 H), 9.93 (s, 1 H) ppm. ^13^C NMR (101 MHz; CD_2_Cl_2_): δ = 155.09, 154.92, 153.7, 140.8, 137.2, 136.3, 135.9, 134.9, 131.7, 131.0, 129.6, 126.1, 124.6, 124.3, 105.6, 101.8, 100.0, 38.4, 35.2, 34.9, 30.3, 29.80, 29.69 ppm. UV–vis (CH_2_Cl_2_) λ_max_ (log ε) = 402 (Soret, 5.50), 509 (4.18), 546 (4.20), 577 (3.92), 584 (sh), 634 (3.95) nm. Fl (λ_excitation_ = λ_Soret_) λ_max_ (rel. intensity) = 636 (1.0), 704 (0.18) nm. HR-MS ESI+ (100% CH_3_CN, 30 V cone voltage, TOF detection): *m*/*z* calc’d for C_31_H_36_N_4_O_2_ [M]^+^ 496.2838; found 496.2845.

**16B**: R_f_ (silica–40% CH_2_Cl_2_/hexanes) = 0.61. UV–vis (CH_2_Cl_2_) λ_max_ (rel. I) = 402 (Soret, 1.0), 509 (0.047), 545 (0.050), 577 (0.026), 584 (sh), 633 (0.028) nm. MS ESI+ (100% CH_3_OH, 30 V cone voltage, TOF detection): *m*/*z* calc’d for C_31_H_37_N_4_O_2_ [M + H]^+^ 497.2911; found 497.2900.

### 3.11. Hydride reduction of meso-Tetrahexylporphyrin-7,8-dione (***10***): Formation of meso-Tetrahexyl-7,8-trans-dihydroxychlorin (***17***)

Prepared according to General Procedure A from dione **10** (25 mg, 0.03 mmol) in 95:5 CH_2_Cl_2_/MeOH (3 mL) and NaBH_4_ (10 mg, 0.26 mmol) over 90 min at 0 °C. Chromatographic purification of the crude material (silica–95:5 CH_2_Cl_2_/ethyl acetate) provided chlorin **17** in 52% yield (13 mg) as a purple solid.

**17**: ^1^H NMR (250 MHz, CDCl_3_): δ = –1.91 (br s, 2 H, NH), 0.90–0.99 (m, 12 H, 4 × CH_3_), 1.33–1.57 (m, 16 H, 8 × CH_2_), 1.69–1.85 (m, 8 H, 4 × CH_2_), 2.22–2.47 (m, 8 H, 4 × CH_2_), 4.24–4.34 (m, 4 H, 2 × CH_2_), 4.63–4.73 (m, 4 H, 2 × CH_2_), 6.26 (s, 2 H, β-H), 8.48 (d, *J* = 4.9 Hz, 2 H, β-H), 9.17 (s, 2 H, β-H), 9.26 (d, *J* = 4.9 Hz, 2 H, β-H) ppm. UV–vis (CH_2_Cl_2_): λ_max_ (log ε): 409 (5.48), 426 sh (5.36), 528 (4.46), 558 (4.53), 594 (4.25), 648 (4.35) nm. HRMS (ESI): *m*/*z* calc’d for C_44_H_65_N_4_O_2_^+^ ([M + H]^+^): 681.5102; found: 681.5091.

### 3.12. Methyl-Grignard Addition to meso-Tetrahexylporphyrin-7,8-dione (***10***): Formation of meso-Tetrahexyl-8-hydroxy-8-methyl-chlorin-7-one (***18***) and meso-Tetrahexyl-7,8-dihydroxy-7,8-dimethyl-chlorin (***19***)

Dione **10** (50 mg, 0.07 mmol) was dissolved in dry THF (3 mL) and a 1 M solution of MeMgBr in THF (4 mL, 4 mmol) was added at –45 °C under inert conditions; the mixture was allowed to warm to ambient temperature and stirred for 3 d. After this time, water was added and the product mixture extracted with CH_2_Cl_2_ or ethyl acetate. The combined organic phases were dried over Na_2_SO_4_ (anhydr.) and chromatographed (silica–2:1 CH_2_Cl_2_/hexane); the first fraction of **18** was in a 24% (14 mg) and the second fraction of **19** in 19% (10 mg) yield, both as purple solids, after their crystallization from CH_2_Cl_2_/MeOH. Adding only 2 mL (2 mmol) of the 1 M MeMgBr solution in THF to dione **10** (50 mg, 0.07 mmol), dissolved in dry THF (3 mL) for a 5 h reaction time at –45 °C, followed by the same workup and chromatography conditions, yields only product **18** in 66% (34 mg) yield (66%)

**18**: ^1^H NMR (500 MHz, CDCl_3_): δ = –1.89 (s, 1 H, NH) –1.69 (s, 1 H, NH), 0.92–0.98 (m, 12 H, 4 × CH_3_), 1.35–1.53 (m, 16 H, 8 × CH_2_), 1.71–1.84 (m, 8 H, 4 × CH_2_), 1.94 (s, 3 H, β-CH_3_), 1.96–2.28 (m, 4 H, 2 × CH_2_), 2.38–2.45 (m, 4 H, 2 × CH_2_), 3.56 (s, 1 H, β-OH), 4.48–4.55 (m, 1 H, CH_A_), 4.62–4.78 (m, 7 H, 3 × CH_2_, CH_B_), 9.15 (d, *J* = 4.7 Hz, 1 H, β-H), 9.17–9.19 (m, 1 H, β-H), 9.22 (d, *J* = 4.7 Hz, 1 H, β-H), 9.23–9.24 (m, 1 H, β-H), 9.28–9.31 (m, 2 H, β-H) ppm. {^1^H}^13^C NMR (126 MHz, CDCl_3_): δ = 14.32 (CH_3_), 22.90 (CH_2_), 22.99 (CH_2_), 26.07 (β-CH_3_), 30.25 (CH_2_), 30.37 (CH_2_), 30.43 (CH_2_), 30.72 (CH_2_), 31.50 (CH_2_), 32.04 (CH_2_), 32.60 (CH_2_), 35.38 (CH_2_), 35.54 (CH_2_), 36.47 (CH_2_), 37.76 (CH_2_), 38.29 (CH_2_), 81.22 (C-7), 111.80 (*meso*-C), 115.95 (*meso*-C), 120.57 (*meso*-C), 123.29 (β-C), 124.68 (β-C), 124.70 (β-C), 126.17 (β-C), 130.68 (β-C), 131.43 (β-C), 136.66 (α-C), 136.33 (α-C), 138.22 (α-C), 140.39 (α-C), 140.65 (α-C), 152.68 (α-C), 154.53 (α-C), 159.27 (α-C), 212.62 (β-CO) ppm. UV–vis (CH_2_Cl_2_): λ_max_ (log ε): 421 (5.17), 441 (5.07), 541 (4.05), 578 (4.12), 607 (3.90), 662 (3.72) nm. HRMS (ESI): *m*/*z* calc’d for C_45_H_65_N_4_O_2_^+^ ([M + H]^+^): 693.5102; found: 693.5081.

**19**: ^1^H NMR (500 MHz, CDCl_3_): δ = –1.10 (s, 2 H, NH), 0.91–0.97 (m, 12 H, 4 × CH_3_), 1.34–1.50 (m, 16 H, 8 × CH_2_), 1.66–1.76 (m, 14 H, 4 × CH_2_, 2 × β-CH_3_), 1.93–2.01 (m, 2 H, CH_2_), 2.06–2.16 (m, 2 H, CH_2_), 2.33–2.40 (m, 4 H, 2 × CH_2_), 2.88 (s, 2 H, β-OH), 4.26–4.34 (m, 2 H, CH_2_), 4.46–4.53 (m, 2 H, CH_2_), 4.56–4.66 (m, 4 H, 2 × CH_2_), 8.95 (d, *J* = 4.9 Hz, 2 H, β-H), 9.07 (s, 2 H, β-H), 9.17 (d, *J* = 4.9 Hz, 2 H, β-H) ppm. {^1^H}^13^C NMR (126 MHz, CDCl_3_): δ = 14.31 (CH_3_), 22.89 (CH_2_), 23.00 (CH_2_), 23.57 (β-CH_3_), 30.40 (CH_2_), 30.68 (CH_2_), 31.68 (CH_2_), 32.04 (CH_2_), 35.18 (CH_2_), 37.79 (CH_2_), 37.91 (CH_2_), 90.27 (β-C), 111.52 (*meso*-C), 121.99 (*meso*-C), 122.06 (β-C), 125.39 (β-C), 129.60 (β-C), 134.13 (α-C), 141.19 (α-C), 151.72 (α-C), 159.47 (α-C) ppm. UV–vis (CH_2_Cl_2_): λ_max_ (log ε): 415 (4.95), 436 (4.75), 535 (4.03), 565 (4.08), 602 (3.85), 657 (3.99) nm. HRMS (ESI): *m*/*z* calc’d for C_46_H_69_N_4_O_2_^+^ ([M + H]^+^): 709.5415; found: 709.5432.

### 3.13. Trifluoromethylation of meso-Tetrahexylporphyrin-7,8-dione (***10***): Formation of meso-Tetrahexyl-8-hydroxy-8-trifluoromethyl-chlorin-7-one (***18^F^***)

Dione **10** was dissolved in THF (3 mL) and trifluoromethyltrimethylsilane (50 µL, 0.38 mmol) was added between –35 and –45 °C, in addition to a catalytic quantity of tetrabutylammonium fluoride (TBAF). After 20 min of stirring, additional TBAF (33 mg, 0.11 mmol) was added and the mixture stirred for 10 min. The mixture was allowed to warm, water was added, and the product extracted with CH_2_Cl_2_ or ethyl acetate. The combined organic fractions were dried over Na2SO4 (anhydr.), the solvent removed using rotary evaporation, and the residue chromatographed (silica–1:1 CH_2_Cl_2_/hexane). Recrystallization of the main fraction from CH_2_Cl_2_/MeOH provided **18^F^** in 73% yield (40 mg) as a purple solid.

**18^F^**: ^1^H NMR (500 MHz, CDCl_3_): δ = –1.76 (s, 1 H, NH), –1.62 (s, 1 H, NH), 0.91–1.00 (m, 12 H, 4 × CH_3_), 1.35–1.53 (m, 16 H, 8 × CH_2_), 1.69–1.86 (m, 8 H, 4 × CH_2_), 1.99–2.15 (m, 3 H, CH_2_, CH_A_), 2.25–2.35 (m, 1 H, CH_B_), 2.36–2.46 (m, 4 H, 2 × CH_2_), 4.45–4.57 (m, 4 H, CH_2_, CH_A_, 1 × β-OH), 4.65–4.77 (m, 5 H, 4 × CH_2_, CH_B_), 9.15–9.17 (m, 1 H, β-H), 9.19–9.21 (m, 2 H, β-H), 9.22–9.24 (m, 1 H, β-H), 9.30–9.33 (m, 2 H, β-H) ppm. {^1^H}^13^C NMR (126 MHz, CDCl_3_): δ = 14.30 (CH_3_), 14.34 (CH_3_), 22.89 (CH_2_), 22.99 (CH_2_), 30.15 (CH_2_), 30.37 (CH_2_), 30.42 (CH_2_), 30.70 (CH_2_), 31.72 (CH_2_), 31.97 (CH_2_), 32.02 (CH_2_), 33.22 (CH_2_), 35.52 (CH_2_), 36.31 (CH_2_), 38.10 (CH_2_), 38.31 (CH_2_), 38.34 (CH_2_), 113.41 (*meso*-C), 115.09 (*meso*-C),122.10 (*meso*-C), 123.88 (β-C), 124.97 (β-C), 125.26 (β-C), 126.22 (β-C), 131.12 (β-C), 131.63 (β-C), 136.09 (α-C), 136.36 (α-C), 138.36 (α-C), 139.88 (α-C), 140.39 (α-C), 149.11 (α-C), 153.34 (α-C), 154.60 (α-C), 205.89 (β-CO) ppm. ^19^F NMR (471 MHz, CDCl_3_): δ = –75.36 (s, 3 F, CF_3_) ppm. UV–vis (CH_2_Cl_2_): λ_max_ (log ε): 426 (5.16), 449 (5.12), 552 (4.10), 588 (4.19), 612 (4.13), 668 (3.99) nm. HRMS (ESI): *m*/*z* calc’d for C_45_H_62_F_3_N_4_O_2_+ ([M + H]+): 747.4819; found: 747.4822.

### 3.14. Hydride Reduction of meso-Tetrahexyl-8-hydroxy-8-methyl-chlorin-7-one (***18***): Formation of meso-Tetrahexyl-7,8-dihydroxy-8-methyl-chlorin (***20***)

According to General Procedure A, *meso*-tetrahexyl-7,8-dihydroxy-8-methyl-chlorin **20** was obtained from *meso*-tetrahexyl-8-hydroxy-8-methyl-chlorin-7-one (**18**) (25 mg, 0.04 mmol) in 9:1 CH_2_Cl_2_ (3 mL) at 0 °C and NaBH_4_ (5 mg, 0.13 mmol) over 90 min. Chromatographic purification (silica–95:5 CH_2_Cl_2_/ethyl acetate) and recrystallization from CH_2_Cl_2_/ethyl MeOH provided **20** in 80% yield (20 mg) as a purple solid.

**20**: ^1^H NMR (500 MHz, CDCl_3_): δ = –1.73 (s, 2 H, NH), 0.91–0.98 (m, 12 H, 4 × CH_3_), 1.35–1.52 (m, 16 H, 8 × CH_2_), 1.64–1.79 (m, 8 H, 4 × CH_2_), 2.07–2.23 (m, 7 H, 2 × CH_2_, 1 × β-CH_3_), 2.36–2.45 (m, 4 H, 2 × CH_2_), 4.26–4.45 (m, 3 H, CH_2_, CH_A_), 4.51–4.74 (m, 5 H, 2 × CH_2_, CH_B_), 6.38 (s, 1 H, β-H), 8.98 (d, *J* = 5.0 Hz, 1 H, β-H), 9.01 (d, *J* = 4.9 Hz, 1 H, β-H), 9.11–9.14 (m, 2 H, β-H), 9.21 (d, *J* = 5.0 Hz, 1 H, β-H), 9.23 (d, *J* = 4.9 Hz, 1 H, β-H) ppm. {^1^H}^13^C NMR (126 MHz, CDCl_3_): δ = 14.32 (CH_3_), 22.52 (β-CH_3_), 22.91 (CH_2_), 22.95 (CH_2_), 22.98 (CH_2_), 30.40 (CH_2_), 30.44 (CH_2_), 30.54 (CH_2_), 32.05 (CH_2_), 32.54 (CH_2_), 33.95 (CH_2_), 35.22 (CH_2_), 35.53 (CH_2_), 36.79 (CH_2_), 37.51 (CH_2_), 38.07 (CH_2_), 38.17 (CH_2_), 85.32 (β-C), 87.37 (β-C), 111.04 (*meso*-C), 111.31 (*meso*-C), 121.54 (*meso*-C), 121.92 (*meso*-C), 121.15 (β-C), 122.40 (β-C), 125.19 (β-C), 125.21 (β-C), 129.80 (β-C), 130.01 (β-C), 134.28 (α-C), 134.74 (α-C), 140.05 (α-C), 140.86 (α-C), 152.11 (α-C), 152.43 (α-C), 158.06 (α-C), 161.69 (α-C) ppm. UV–vis (CH_2_Cl_2_): λ_max_ (log ε): 410 (5.18), 431 (5.02), 531 (4.09), 559 (4.21), 598 (3.88), 652 (4.07) nm. HRMS (ESI+, TOF detection): *m*/*z* calc’d for C_45_H_67_N_4_O_2_+ ([M + H]^+^): 695.5259; found: 695.5240.

### 3.15. Hydride Reduction of meso-Tetrahexyl-8-hydroxy-8-trifluoromethyl-chlorin-7-one (***18^F^***): meso-Tetrahexyl-7,8-dihydroxy-8-trifluoromethyl-chlorin (***20^F^***)

According to General Procedure A, *meso*-tetrahexyl-7,8-dihydroxy-8-trifluoromethyl-chlorin **20^F^** was obtained from *meso*-tetrahexyl-8-hydroxy-8-trifluoromethyl-chlorin-7-one (**18^F^**) (25 mg, 0.03 mmol) in 9:1 CH_2_Cl_2_ (3 mL) at 0 °C and NaBH_4_ (5 mg, 0.13 mmol) over 90 min. Chromatographic purification (silica–95:5 CH_2_Cl_2_/ethyl acetate) and recrystallization from CH_2_Cl_2_/MeOH provided **20^F^** in 80% yield (21 mg) as a purple solid.

**20^F^**: ^1^H NMR (500 MHz, CDCl_3_): δ = –1.51 (br s, 2 H, NH), 0.88–0.99 (m, 12 H, 4 × CH_3_), 1.26–1.61 (m, 18 H, 9 × CH_2_), 1.71–1.82 (m, 6 H, 3 × CH_2_), 1.95–2.02 (m, 2 H, CH_2_), 2.16–2.33 (m, 2 H, CH_2_), 2.36–2.44 (m, 4 H, 2 × CH_2_), 2.84–2.93 (m, 1 H, β-OH), 3.93 (br s, 1 H, β-OH), 4.20–4.28 (m, 1 H, CH_A_), 4.35–4.46 (m, 3 H, CH_2_, CH_B_), 4.56–4.72 (m, 4 H, 2 × CH_2_), 6.93–6.96 (m, 1 H, β-H), 8.97 (d, *J* = 5.1 Hz, 1 H, β-H), 9.03 (d, *J* = 5.0 Hz, 1 H, β-H), 9.09 (d, *J* = 4.6 Hz, 1 H, β-H), 9.13 (d, *J* = 4.6 Hz, 1 H, β-H), 9.20–9.22 (m, 2 H, β-H) ppm. {^1^H}^13^C NMR (126 MHz, CDCl_3_): δ =14.25 (CH_3_), 14.30 (CH_3_), 22.89 (CH_2_), 23.00 (CH_2_), 30.21 (CH_2_), 30.39 (CH_2_), 30.44 (CH_2_), 30.62 (CH_2_), 31.96 (CH_2_), 32.04 (CH_2_), 32.87 (CH_2_), 33.04 (CH_2_), 35.12 (CH_2_), 35.55 (CH_2_), 36.47 (CH_2_), 38.04 (CH_2_), 38.09 (CH_2_), 38.15 (CH_2_), 89.18 (β-C), 109.89 (*meso*-C), 113.78 (*meso*-C), 121.49 (*meso*-C), 122.52 (β-C), 122.85 (β-C), 123.30 (*meso*-C), 124.95 (β-C), 125.80 (β-C), 129.88 (β-C), 130.41 (β-C), 134.24 (α-C), 135.02 (α-C), 140.30 (α-C), 140.58 (α-C), 149.30 (α-C), 153.15 (α-C), 153.25 (α-C), 155.25 (α-C) ppm. {^1^H}^19^F NMR (471 MHz, CDCl_3_): δ = –72.65 (s, 3 F, CF_3_) ppm. UV–vis (CH_2_Cl_2_): λ_max_ (log ε): 409 (5.34), 431 (5.17), 533 (4.22), 562 (4.40), 599 (4.08), 653 (4.24) nm. HRMS (ESI): *m*/*z* calc’d for C_45_H_64_F_3_N_4_O_2_^+^ ([M + H]^+^): 749.4976; found: 749.4955.

## Data Availability

The original contributions presented in the study are included in the article/Supplementary Material; further inquiries can be directed to the corresponding author/s.

## Supplementary Materials

The following supporting information can be downloaded at https://www.mdpi.com/article/10.3390/molecules29092144/s1: reproductions of the key spectra of all new compounds.
