# Supplementary material for: meso-Tetrahexyl-7,8-dihydroxychlorin and Its Conversion to ß-Modified Derivatives
Source: Molecules. 2024 May 5;29(9):2144. doi: 10.3390/molecules29092144 (PMC11085094; doi:10.3390/molecules29092144)
Supplement: Supplementary file 1 [file molecules-29-02144-s001.zip › molecules-2985141-supplementary.pdf]

Supporting Information to

meso-Tetrahexyl-7,8-dihydroxychlorin and its Conversion to ß-Modified Derivatives

Daniel Aicher,[a] Dinusha Damunupola,[b] Christian B.W. Stark,[a,c,*]
Arno Wiehe,[a,d,*] Christian Brückner[b,*]

a Freie Universität Berlin, Institut für Chemie und Biochemie, Takustr. 3, 14195 Berlin, Germany.

b University of Connecticut, Department of Chemistry, 55 N Eagleville Rd., Storrs, CT 06269-3060, U.S.A.

c Current affiliation: Universität Hamburg, Department of Chemistry, Institute of Organic Chemistry, Martin-Luther-King-Platz 6, 20146 Hamburg, Germany

d Current affiliation: biolitec research GmbH, Otto-Schott-Str. 15, 07745 Jena, Germany

Select Reproduction of Spectroscopic Data for:

meso-Tetrahexylporphyrin (**7**) Known compound; data included for comparison. 2

5,15-Dihexylporphyrin (**15**). Known compound; data included for comparison. 4

meso-Tetrahexyl-7,8-*cis*-dihydroxychlorin (**8**) 6

meso-Tetrahexyl-7,8,17,18-*cis*-tetrahydroxybacteriochlorin (**9**). 11

meso-Tetrahexylporphyrin-7,8-dione (**10**). 14

meso-Tetrahexylchlorin-7-one (**11**). 18

meso-Tetrahexyl-7-hydroxychlorin (**12**). 21

meso-Tetrahexylporpholactone (*meso*-Tetrahexyl-7-oxo-8-oxa-porphyrin) (**13**). 24

5-(1'-Oxo-hexyl)-10,15,20-trihexylporphyrin (**14**). 27

5,15-Dihexyl-3-oxo-2-oxa-porphyrin) (**16A**) 30

5,15-Dihexyl-7-oxo-8-oxa-porphyrin) (**16B**). 33

meso-Tetrahexyl-7,8-*trans*-dihydroxychlorin (**17**). 35

meso-Tetrahexyl-8-hydroxy-8-methyl-chlorin-7-one (**18**) 38

meso-Tetrahexyl-7,8-dihydroxy-7,8-dimethyl-chlorin (**19**). 41

meso-Tetrahexyl-8-hydroxy-8-trifluoromethyl-chlorin-7-one (**18^F**). 44

meso-Tetrahexyl-7,8-dihydroxy-8-methyl-chlorin (**20**). 48

meso-Tetrahexyl-7,8-dihydroxy-8-trifluoromethyl-chlorin (**20^F**). 51

meso-Tetrahexylporphyrin (7). Known compound;[1] data included for comparison.

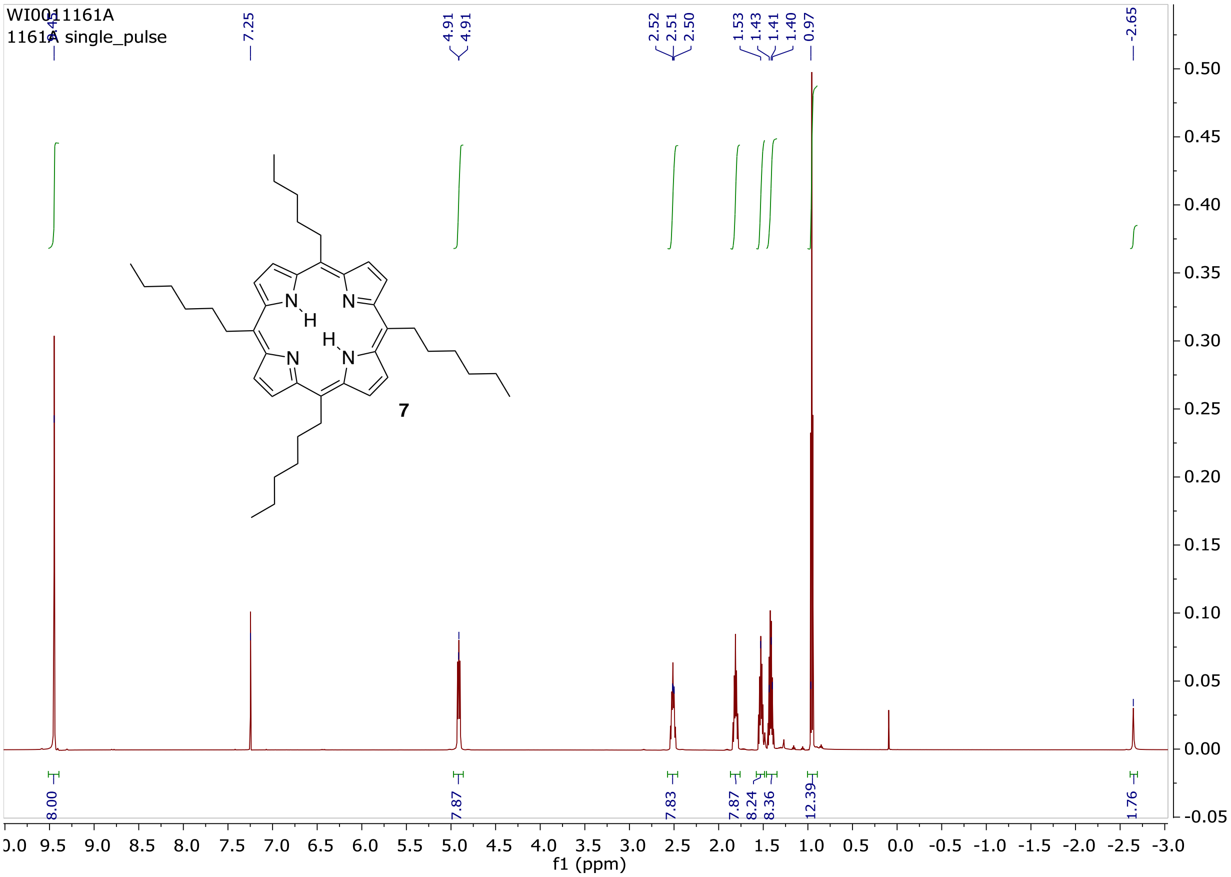

Figure S-1. ^{1}H NMR (600 MHz, $CDCl_3$) spectrum of *meso*-tetrahexylporphyrin **7**.

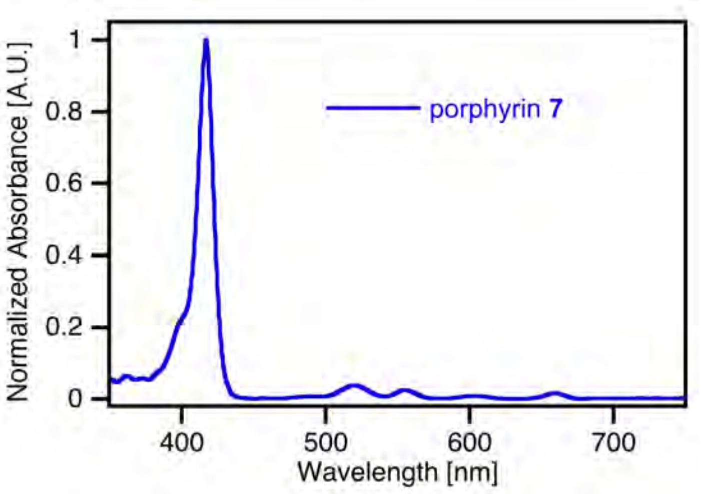

Figure S-2. UV-vis spectrum (CH_2Cl_2) of *meso*-tetrahexylporphyrin **7**.

[1] (a) Senge, M.O.; Bischoff, I.; Nelson, N.Y.; Smith, K.M. Synthesis, reactivity and structural chemistry of 5,10,15,20-tetraalkylporphyrins. *J. Porphyrins Phthalocyanines* **1999**, *3*, 99–116. (b) Wiehe, A.; Shaker, Y.M.; Brandt, J.C.; Mebs, S.; Senge, M.O. Lead structures for applications in photodynamic therapy. Part 1: Synthesis and variation of *m*-THPC (Temoporfin) related amphiphilic A_2BC-type porphyrins. *Tetrahedron* **2005**, *61*, 5535–5564. (c) Plamont, R.; Kikkawa, Y.; Takahashi, M.; Kanesato, M.; Giorgi, M.; Chan Kam Shun, A.; Roussel, C.; Balaban, T.S. Nanoscopic Imaging of *meso*-Tetraalkylporphyrins Prepared in High Yields Enabled by Montmorrilonite K10 and 3 Å Molecular Sieves. *Chem.–Eur. J.* **2013**, *19*, 11293-11300.

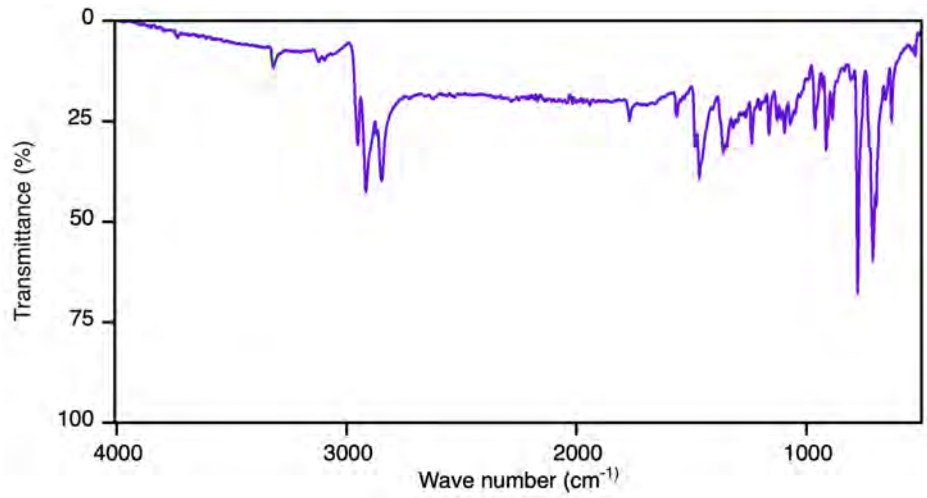

Figure S-3. FT-IR (neat, diamond ATR) spectrum of *meso*-tetrahexylporphyrin **7**.

5,15-Dihexylporphyrin (15). Known compound;[2] data included for comparison.

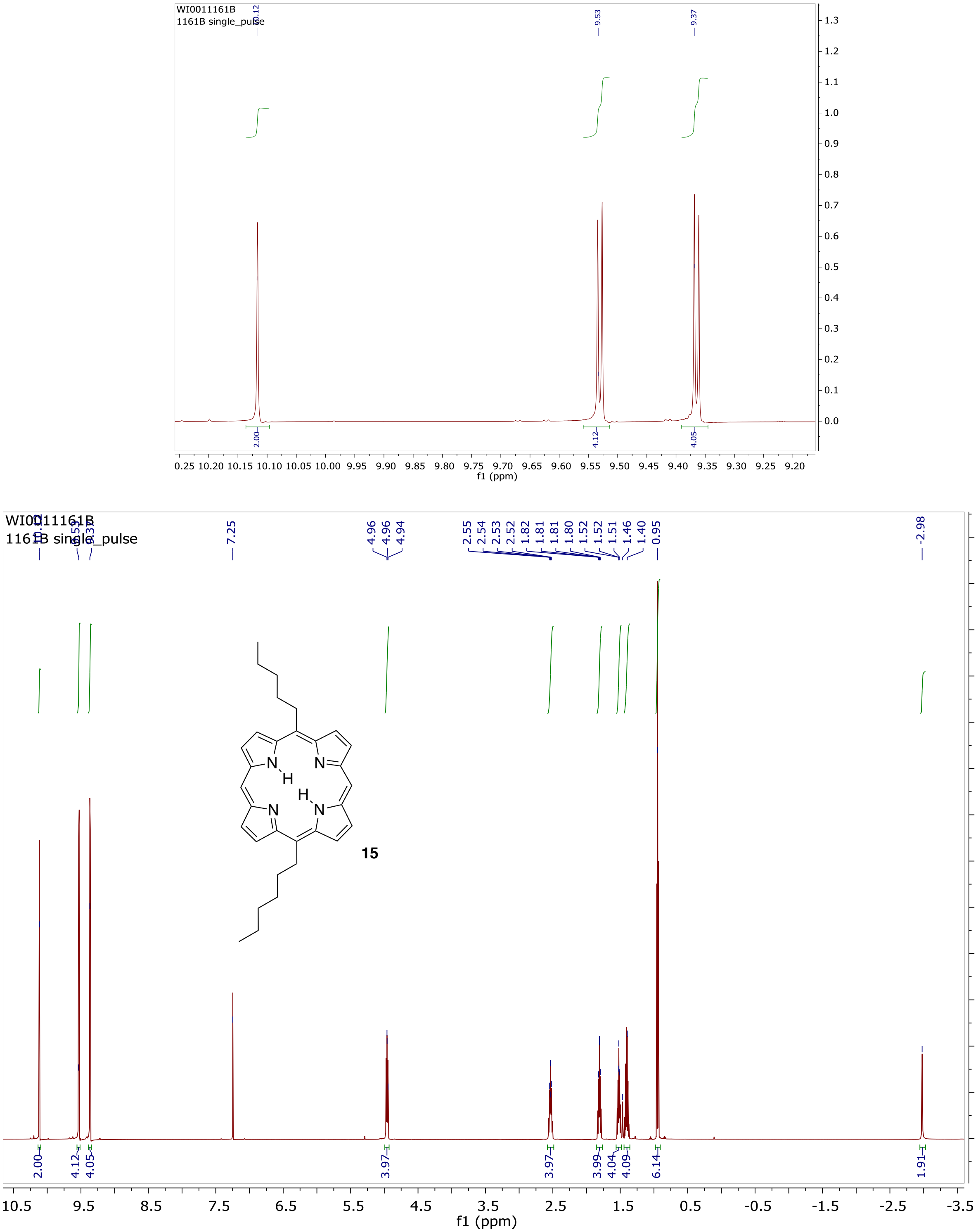

Figure S-1. ^{1}H NMR (600 MHz, $CDCl_3$) spectrum of 5,15-dihexylporphyrin **15** (full spectrum, bottom, and detail, top)

---

[2] Hiroto, S.; Osuka, A. Meso-Alkyl-Substituted meso-meso Linked Diporphyrins and meso-Alkyl-Substituted meso-meso, β–β, β–β Triply Linked Diporphyrins. *J. Org. Chem.* **2005**, *70*, 4054-4058.

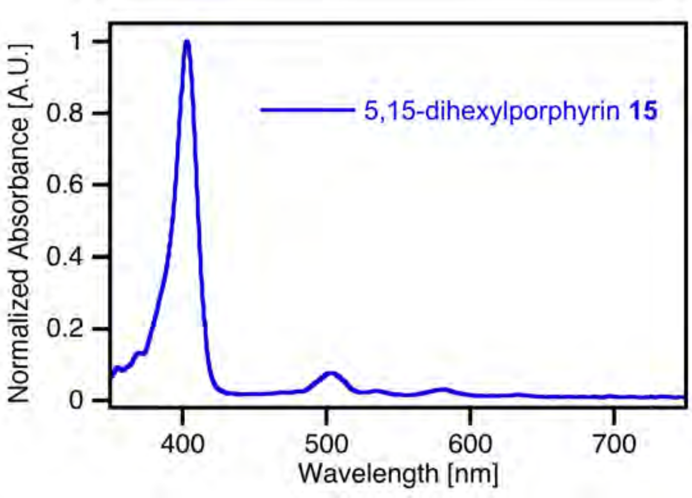

Figure S-2. UV-vis spectrum (CH_2Cl_2) of 5,15-dihexylporphyrin **15**.

meso-Tetrahexyl-7,8-*cis*-dihydroxychlorin (8)

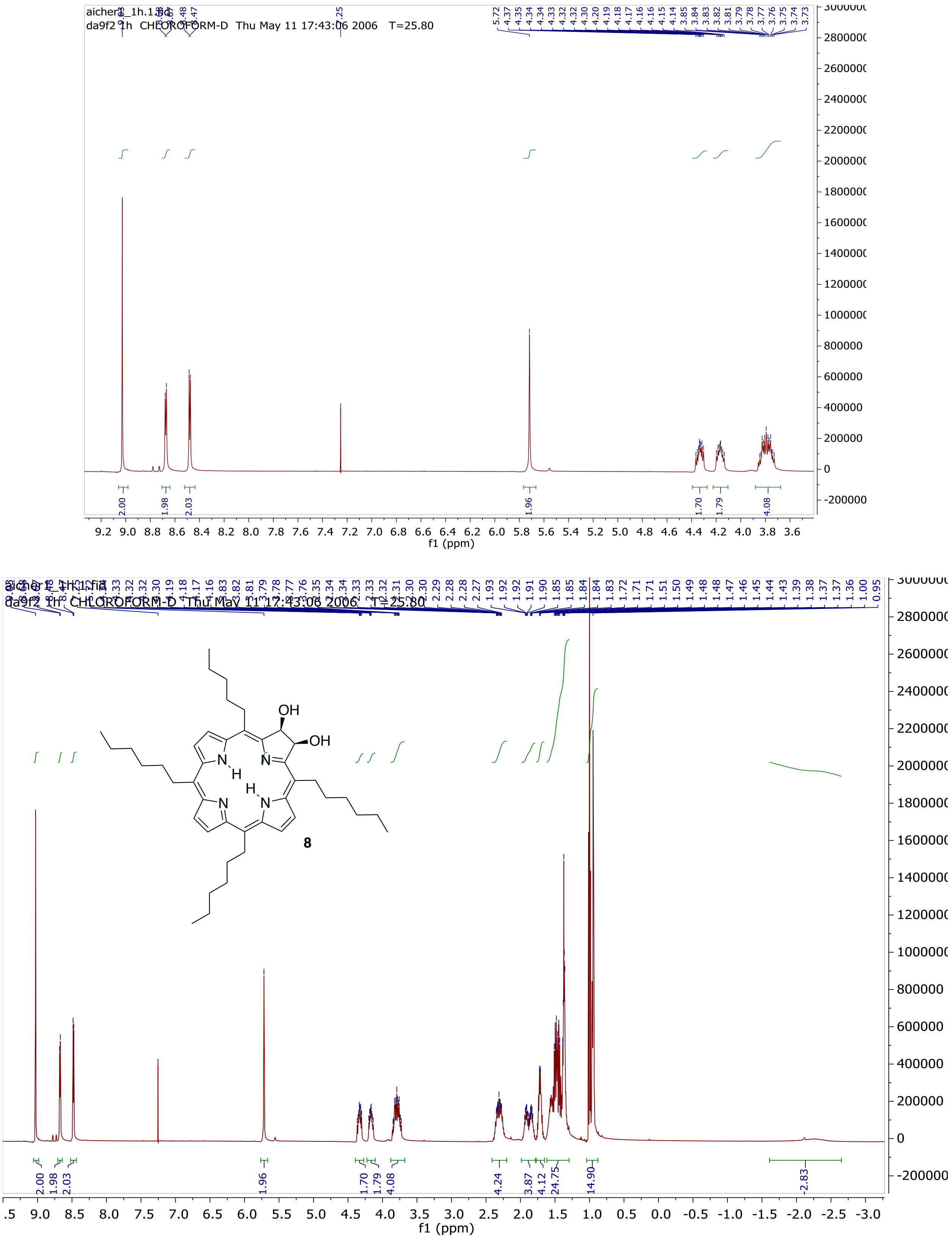

Figure S-1. ^{1}H NMR (500 MHz, $CDCl_3$) spectrum of *meso*-tetrahexyldihydroxychlorin **8** (full spectrum, bottom, and detail, top)

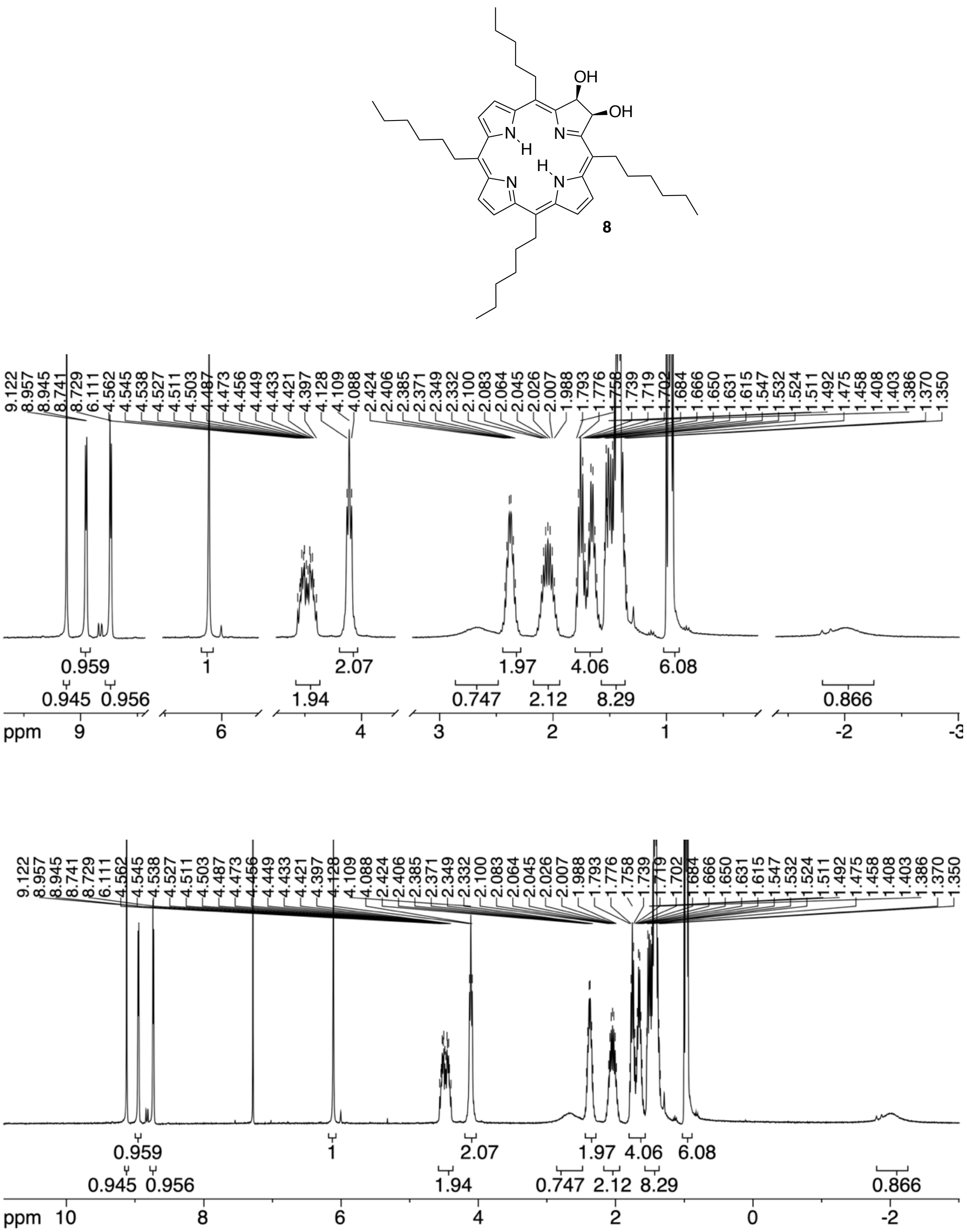

Figure S-2. ^{1}H NMR (400 MHz, $CDCl_3$) spectrum of *meso*-tetrahexyldihydroxychlorin **8** (full spectrum, bottom, and detail, top)

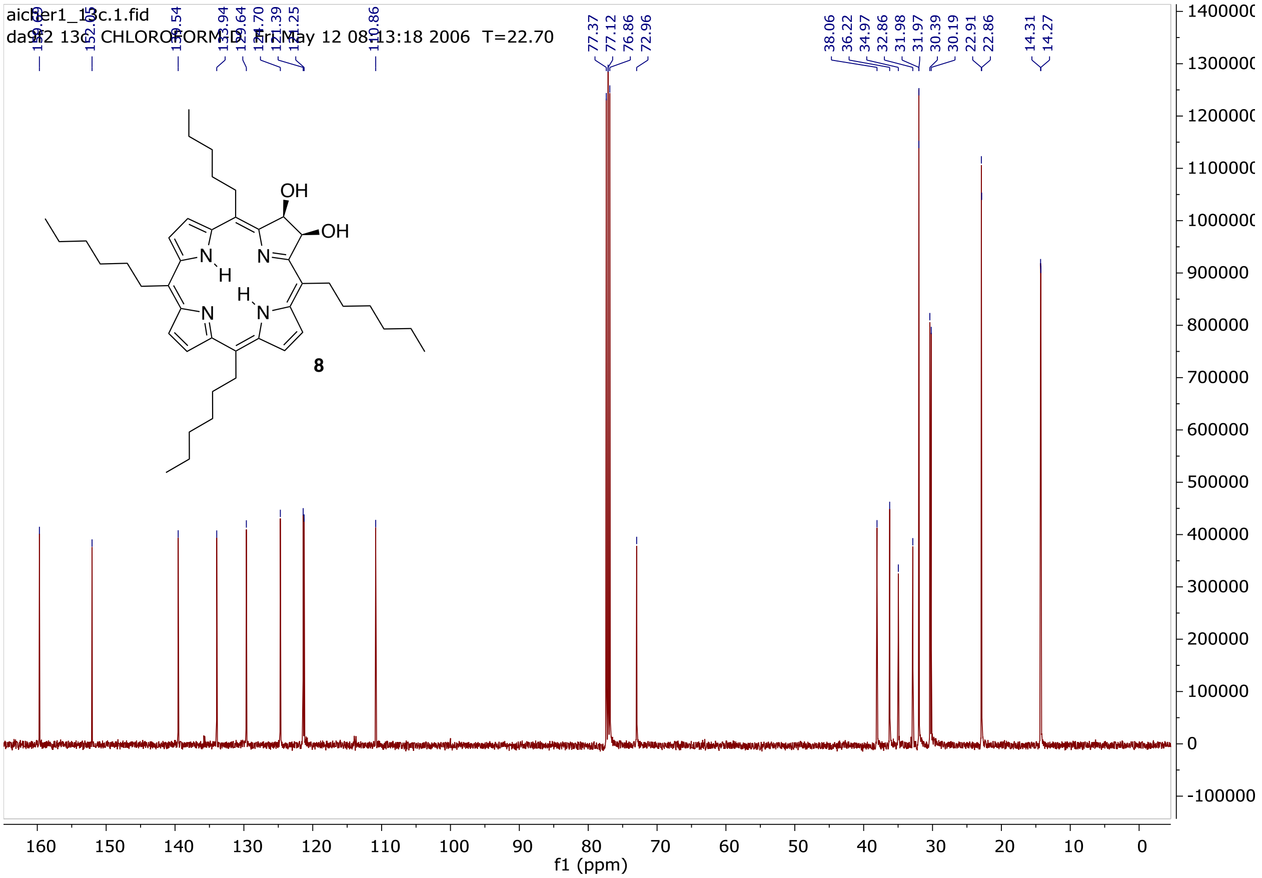

Figure S-3. ^{13}C NMR (126 MHz, $CDCl_3$) spectrum of *meso*-tetrahexyldihydroxychlorin **8**.

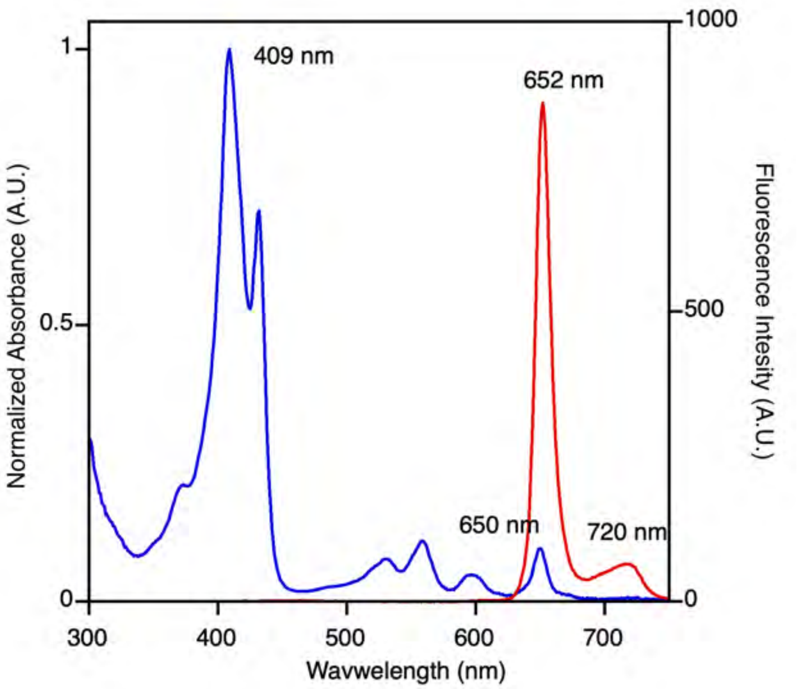

Figure S-4. UV-vis (blue) and fluorescence emission (red) spectra (CH_2Cl_2) of *meso*-tetrahexyldihydroxychlorin **8**; $\lambda_{excitation} = \lambda_{Soret}$.

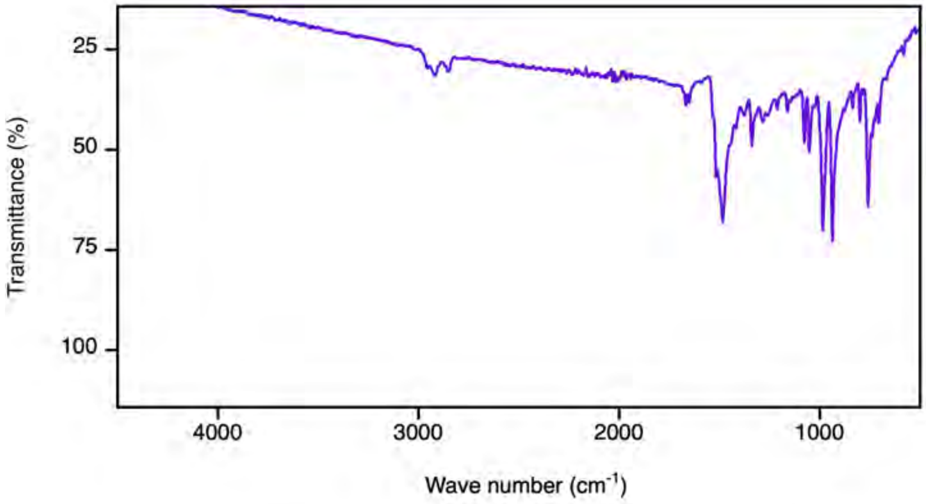

Figure S-5. FT-IR spectrum (neat, ATR) of *meso*-tetrahexyldihydroxychlorin **8**.

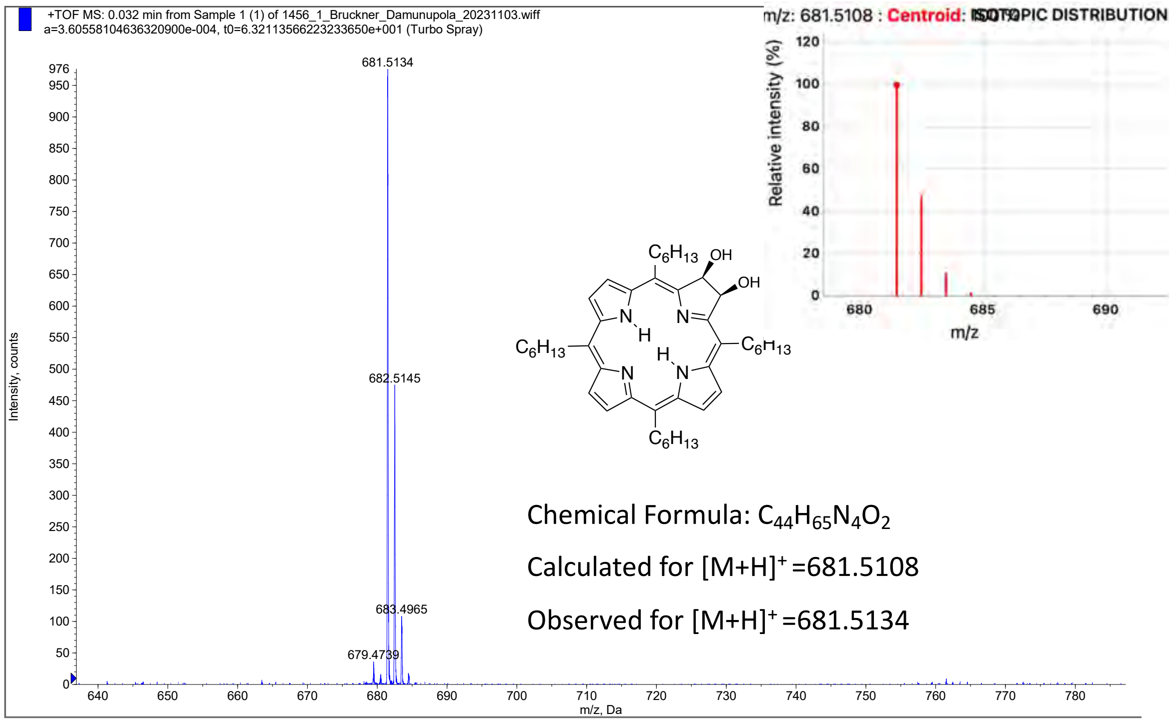

Figure S-6. HR-MS spectrum (ESI+, CH_3CN, TOF) of *meso*-tetrahexyldihydroxychlorin **8**.

Zentrale Meßabteilung Massenspektrometrie - Inst.f.Org.Chemie der Freien Universität Berlin

Hochauflösung - peak-match-Methode - Meßbedingungen:Massenspektrometer MAT 711 der Fa. Finnigan MAT, Bremen - Emission: 80 eV; Elektronenenergie: 0,8 mA; Ionenbeschleunigung: 8 kV; Ionenquellentemperatur: 150 °C - Aufnahmetemperatur: 170 °C - Auflösung: 12.500 (10 % rel. Tal)

Name: Biolitec / D. Aicher   Probenbezeichnung: [DA 9 F 2]

Bearbeitungs-Nr.: 7/28.445   Datum: 25.7.2006

Referenzmasse:	Dekade:	Gefundene Masse:	Berechnete Masse	Summenformel:	Zuordnung:
PFK 654,960125	1,0389980	680,50225	680,50293	$[C_{44}H_{64}N_4O_2]^{+\cdot}$	$[M]^{+\cdot}$

ATOMIC COMPOSITION REPORT (MANUAL)

Selected Isotopes:

Symbol	Min	Max	V'cy	Name
C	0	80	4	Carbon-12
H	0	90	1	Hydrogen-1
O	0	10	2	Oxygen-16
N	0	6	3	Nitrogen-14

Allowable error = minimum of 5.0 ppm, 5.0 mmu.

Mass	Calculated	ppm	mmu	Formula
680.50225	680.50159	-1.0	-0.7	$C_{43}H_{68}O_6$
	680.50293	1.0	0.7	$C_{44}H_{64}O_2N_4$ *
	680.50024	-2.9	-2.0	$C_{41}H_{66}O_5N_3$
	680.50427	3.0	2.0	$C_{46}H_{66}O_3N$
	680.50476	3.7	2.5	$C_{32}H_{68}O_9N_6$
	680.49890	-4.9	-3.3	$C_{39}H_{64}O_4N_6$

***** End of Atomic Composition Report *****

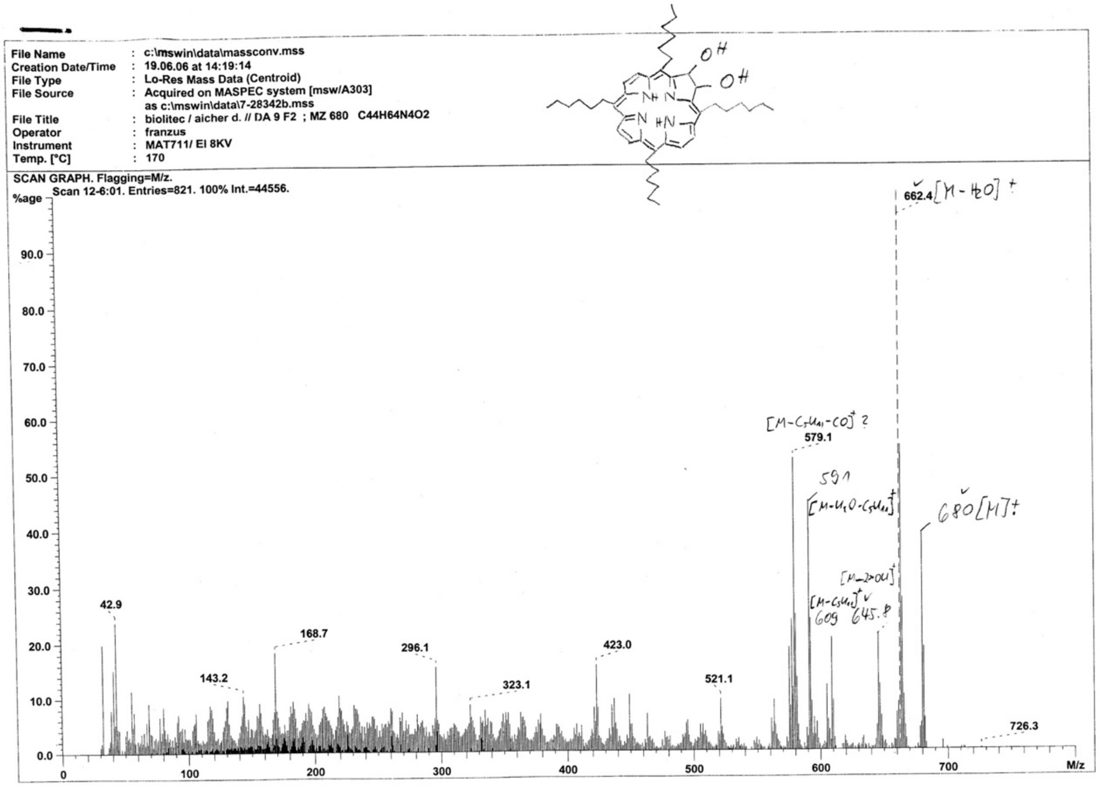

Figure S-7. High-resolution (top) and low-resolution (bottom) mass spectra (EI) of *meso*-tetrahexyldihydroxychlorin **8**.

meso-Tetrahexyl-7,8,17,18-*cis*-tetrahydroxybacteriochlorin (9).

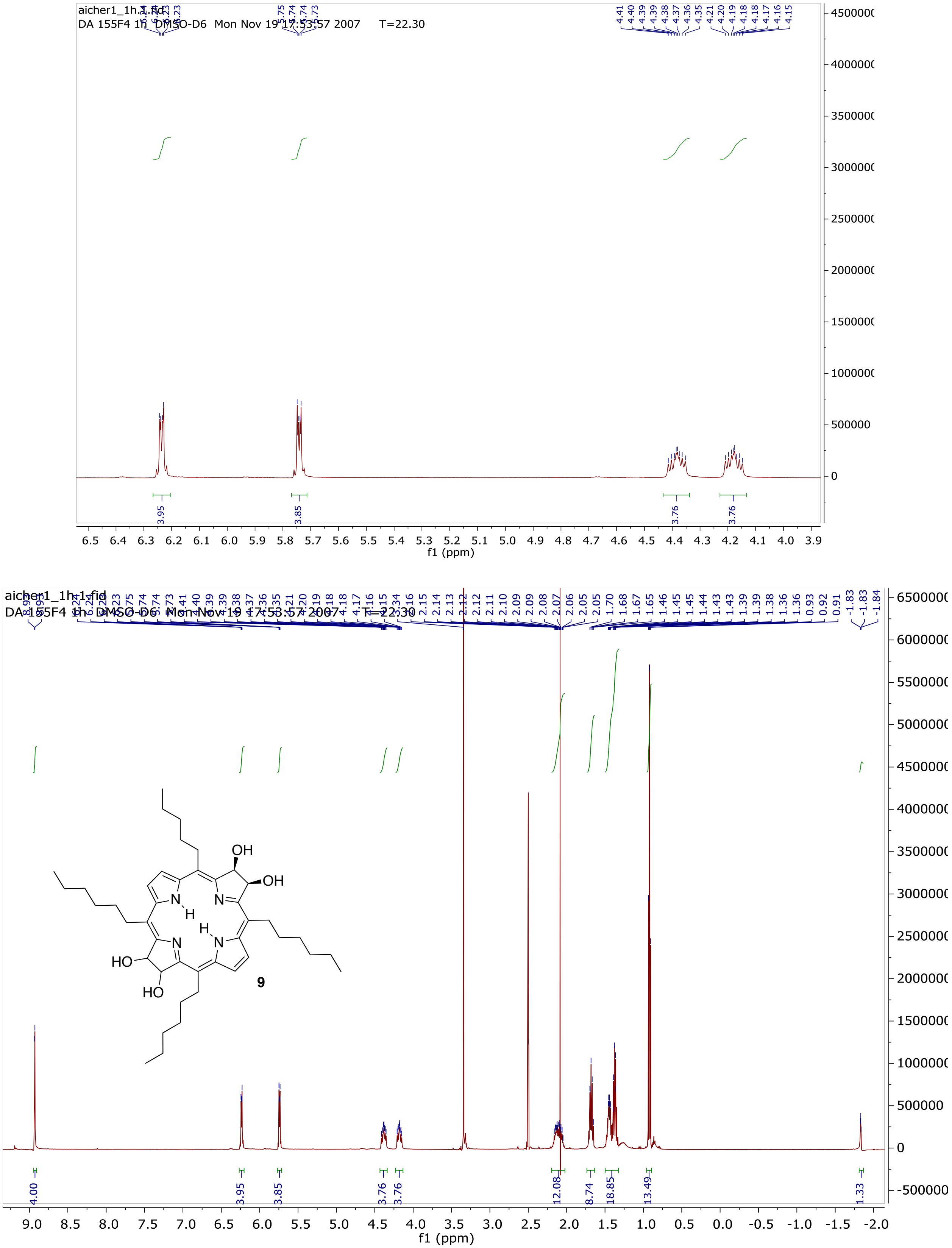

Figure S-1. ^{1}H NMR (500 MHz, DMSO-d_6) spectrum of *meso*-tetrahexyltetrahydroxybacteriochlorin **9** (full spectrum, bottom, and detail, top)

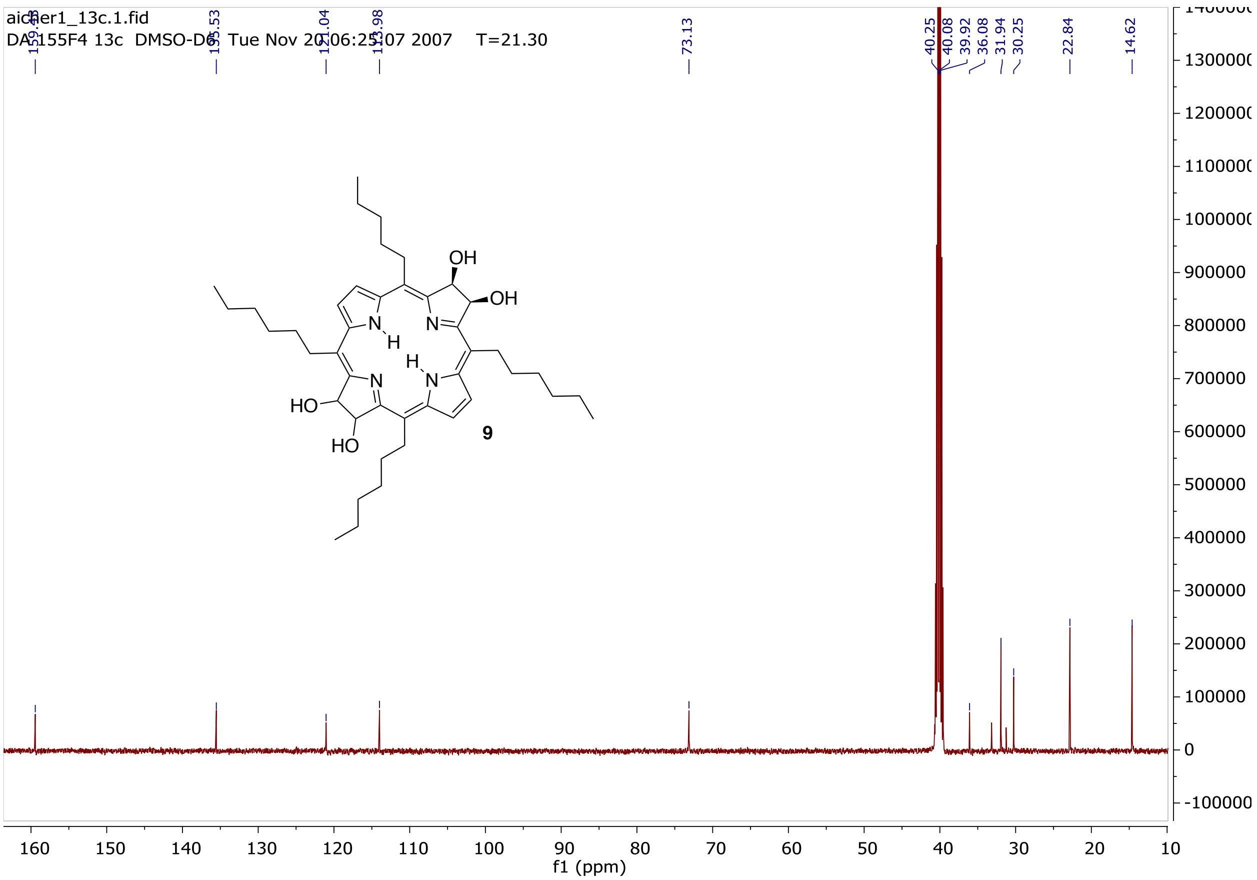

Figure S-2. ^{13}C NMR (126 MHz, DMSO-d$_6$) spectrum of *meso*-tetrahexyltetrahydroxybacteriochlorin **9**.

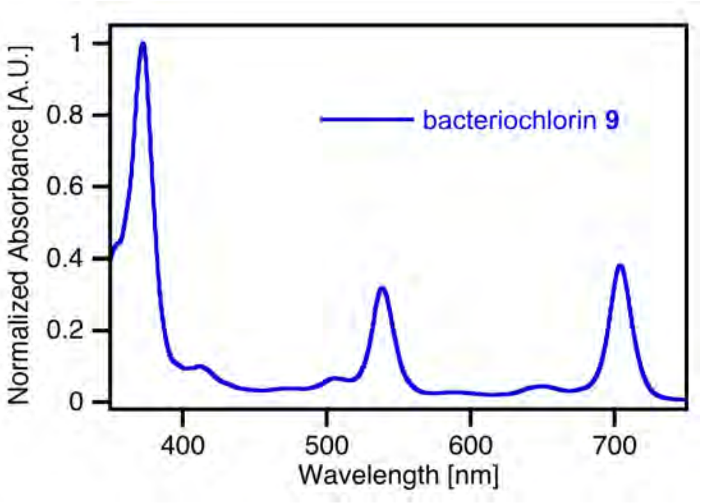

Figure S-3. UV-vis spectrum (CH_2Cl_2) of *meso*-tetrahexyltetrahydroxybacteriochlorin **9**.

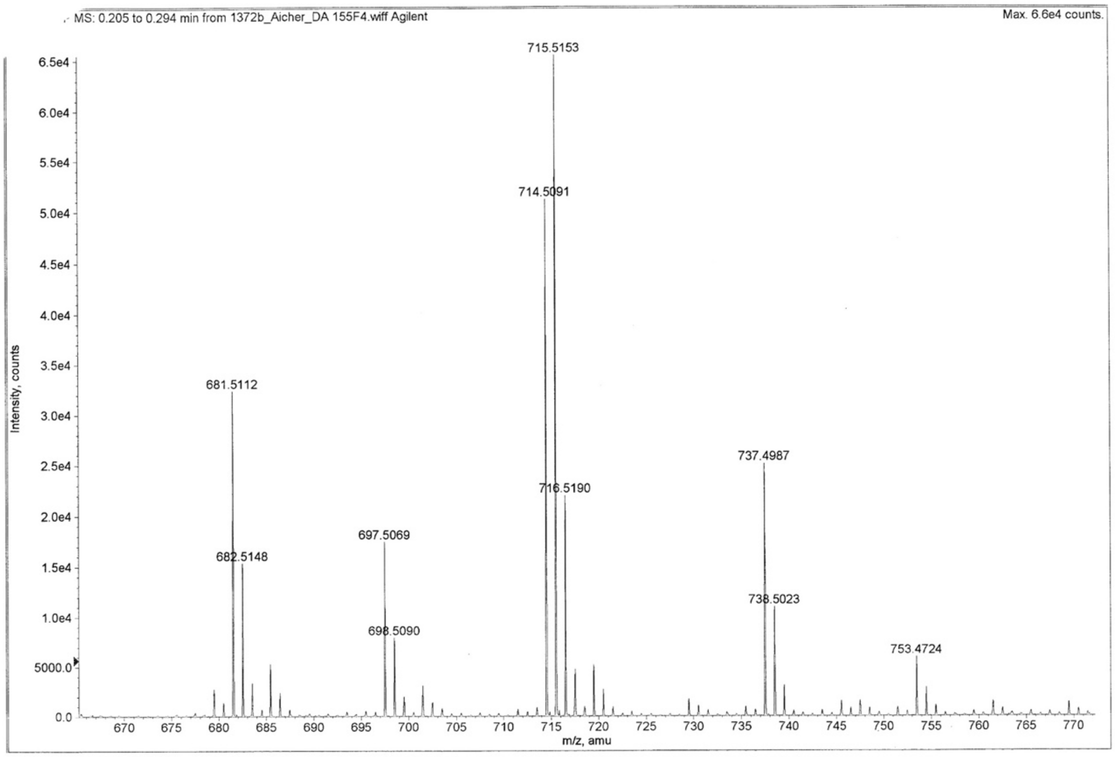

Figure S-4. HR-MS spectrum (ESI+, TOF, CH_3OH) of *meso*-tetrahexyltetrahydroxybacterio–chlorin (**9**).

meso-Tetrahexylporphyrin-7,8-dione (10).

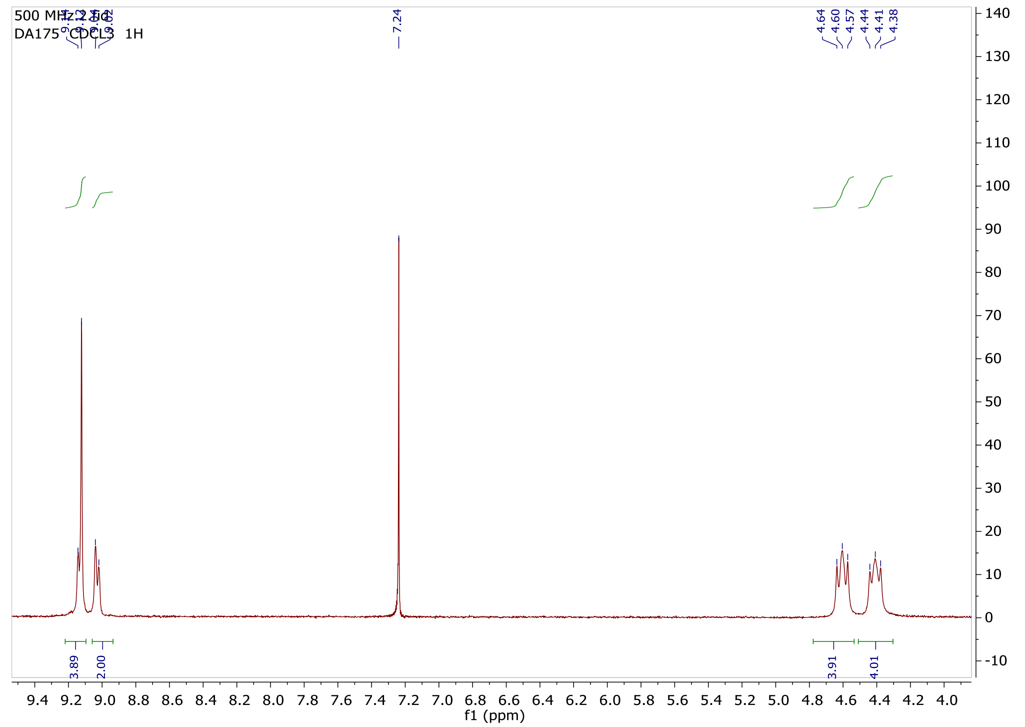

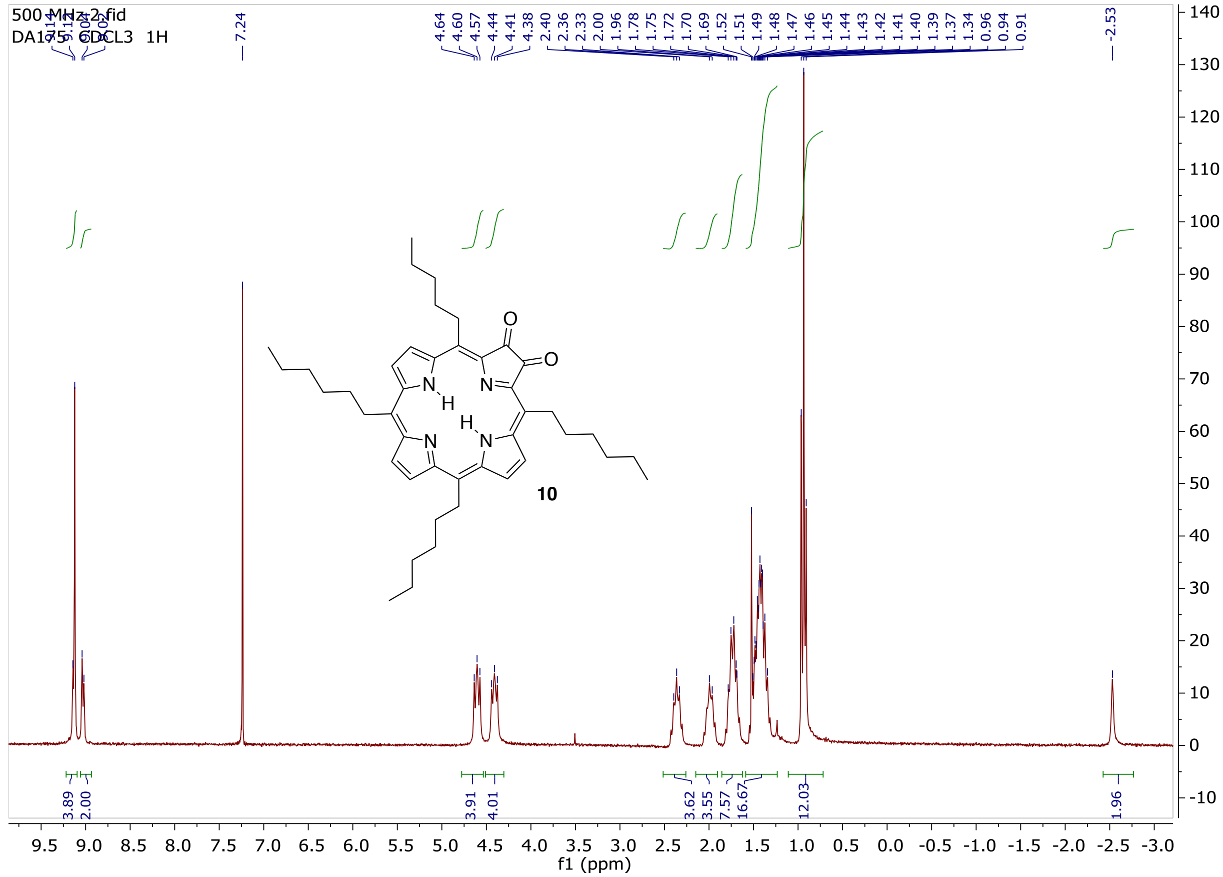

Figure S-1. ^{1}H NMR (500 MHz, $CDCl_3$) spectrum of *meso*-tetrahexylporphyrin-7,8-dione (**10**) (full spectrum, bottom, and detail, top)

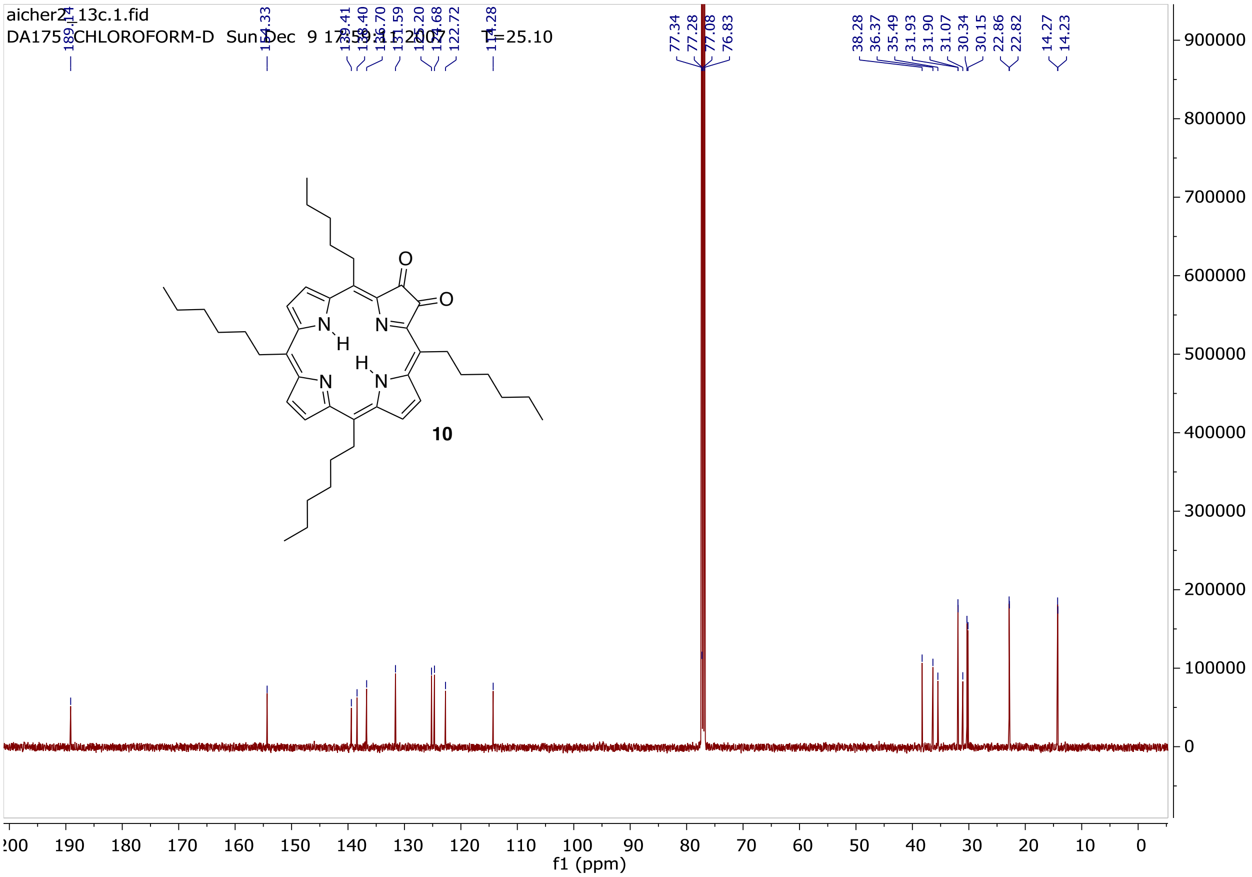

Figure S-2. ^{13}C NMR (126 MHz, $CDCl_3$) spectrum of *meso*-tetrahexylporphyrin-7,8-dione (**10**).

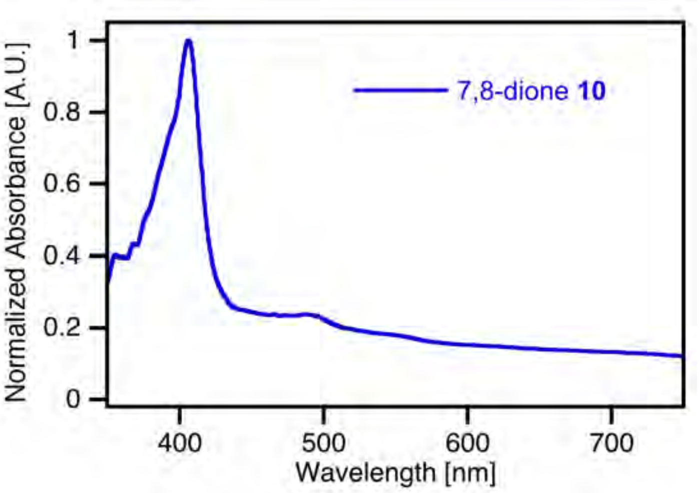

Figure S-3. UV-vis spectrum (CH_2Cl_2) of *meso*-tetrahexylporphyrin-7,8-dione (**10**)

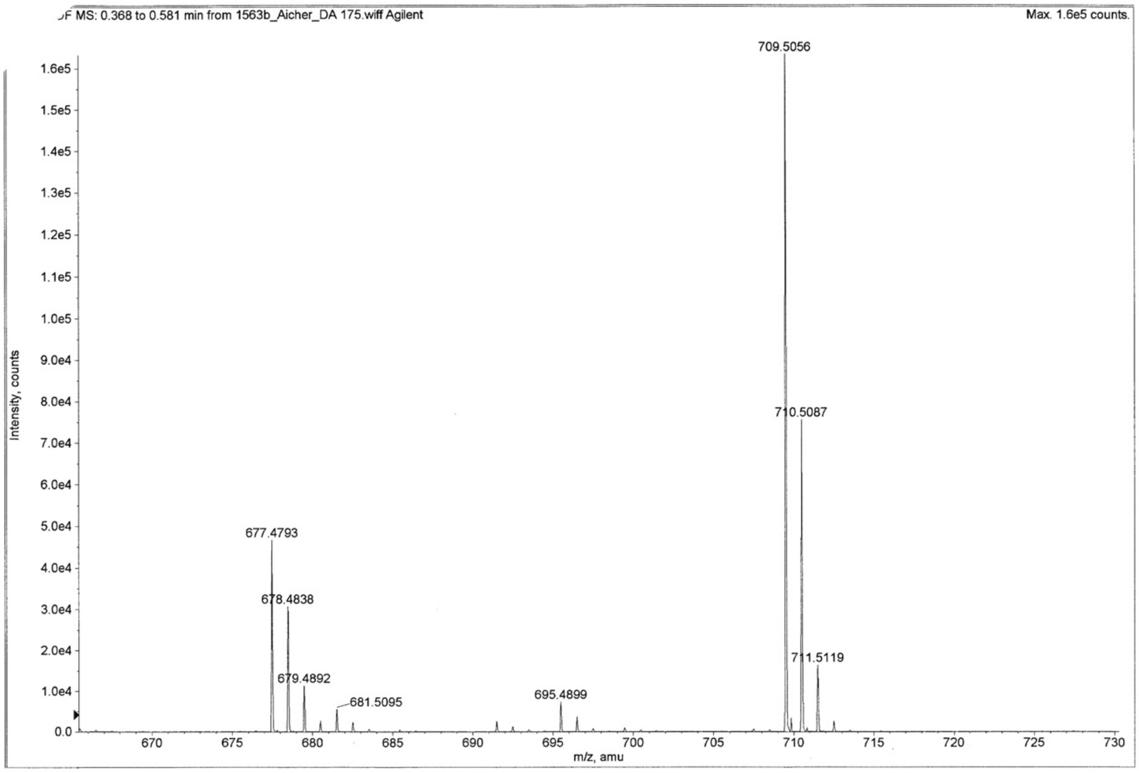

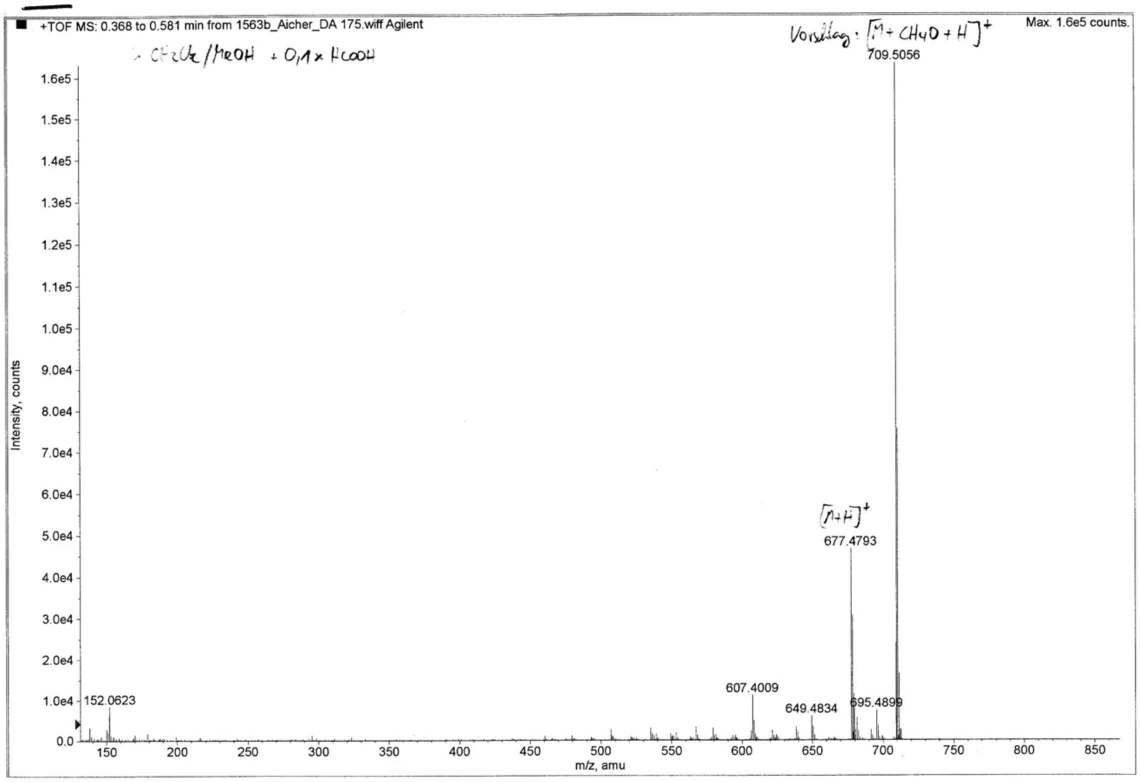

Figure S-4. HR-MS spectrum (ESI+, TOF, MeOH) (full and detail) of *meso*-tetrahexyl-porphyrin-7,8-dione (**10**)

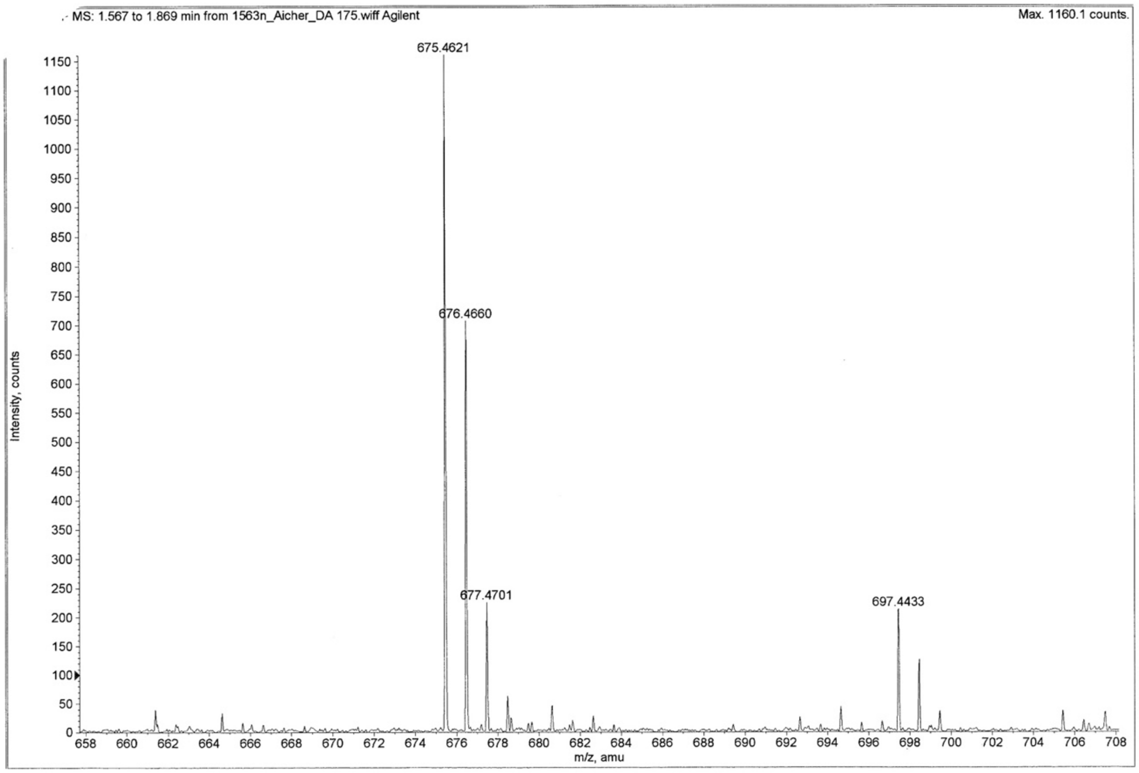

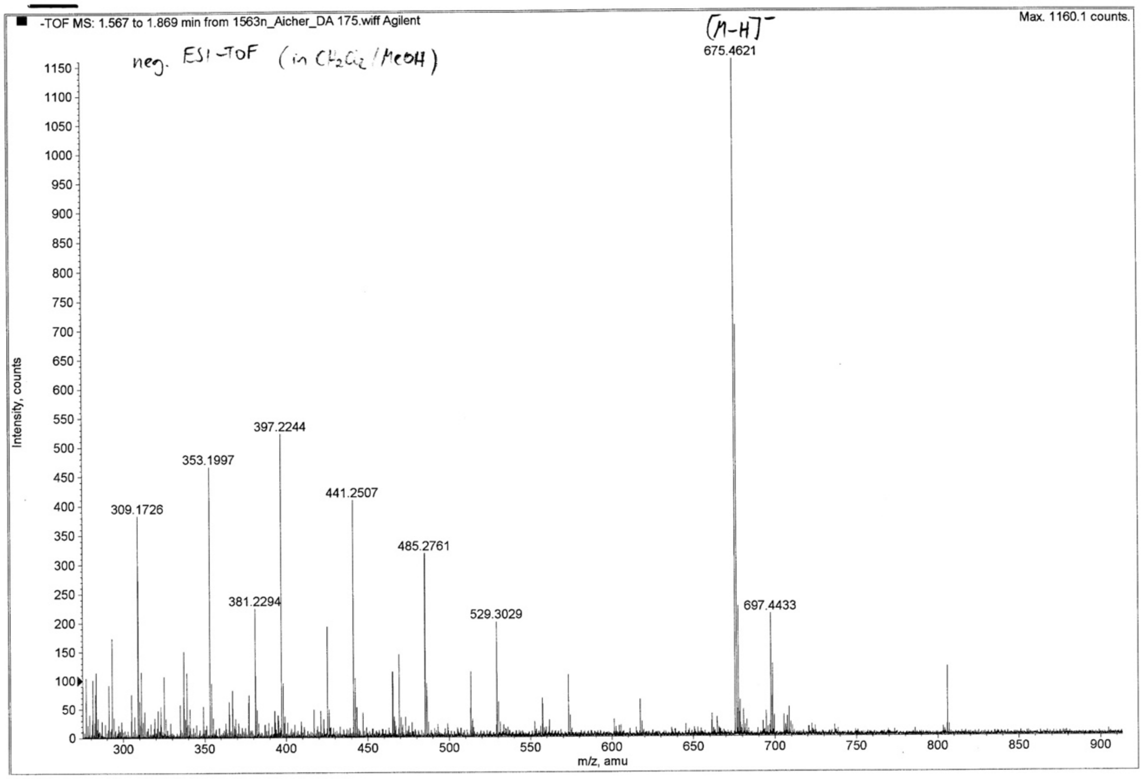

Figure S-5. HR-MS spectrum (ESI–, TOF, CH_2Cl_2/MeOH) (full and detail) of *meso*-tetrahexyl-porphyrin-7,8-dione (**10**).

meso-Tetrahexylchlorin-7-one (11).

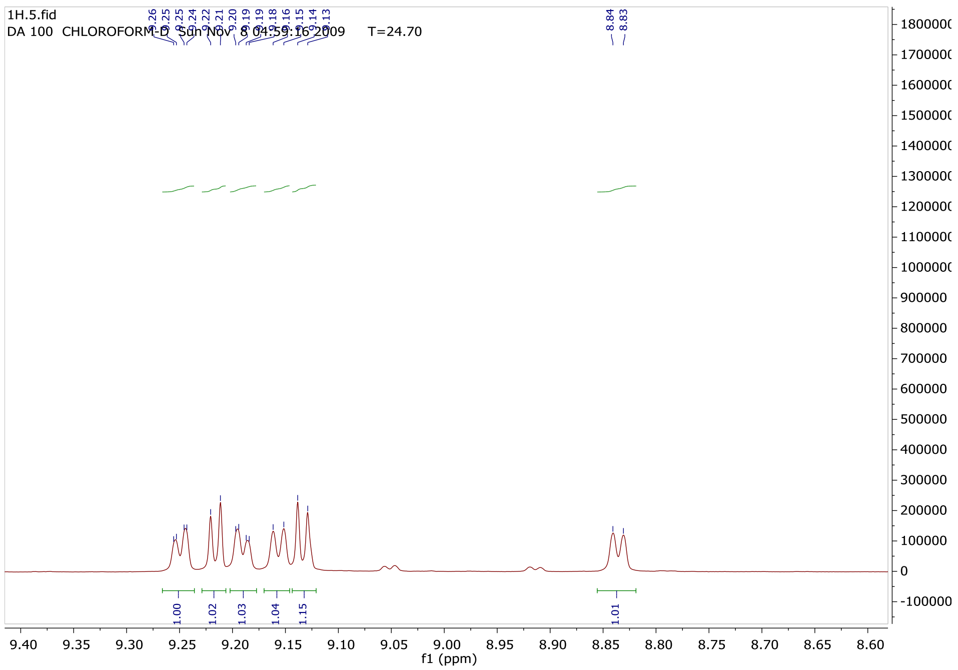

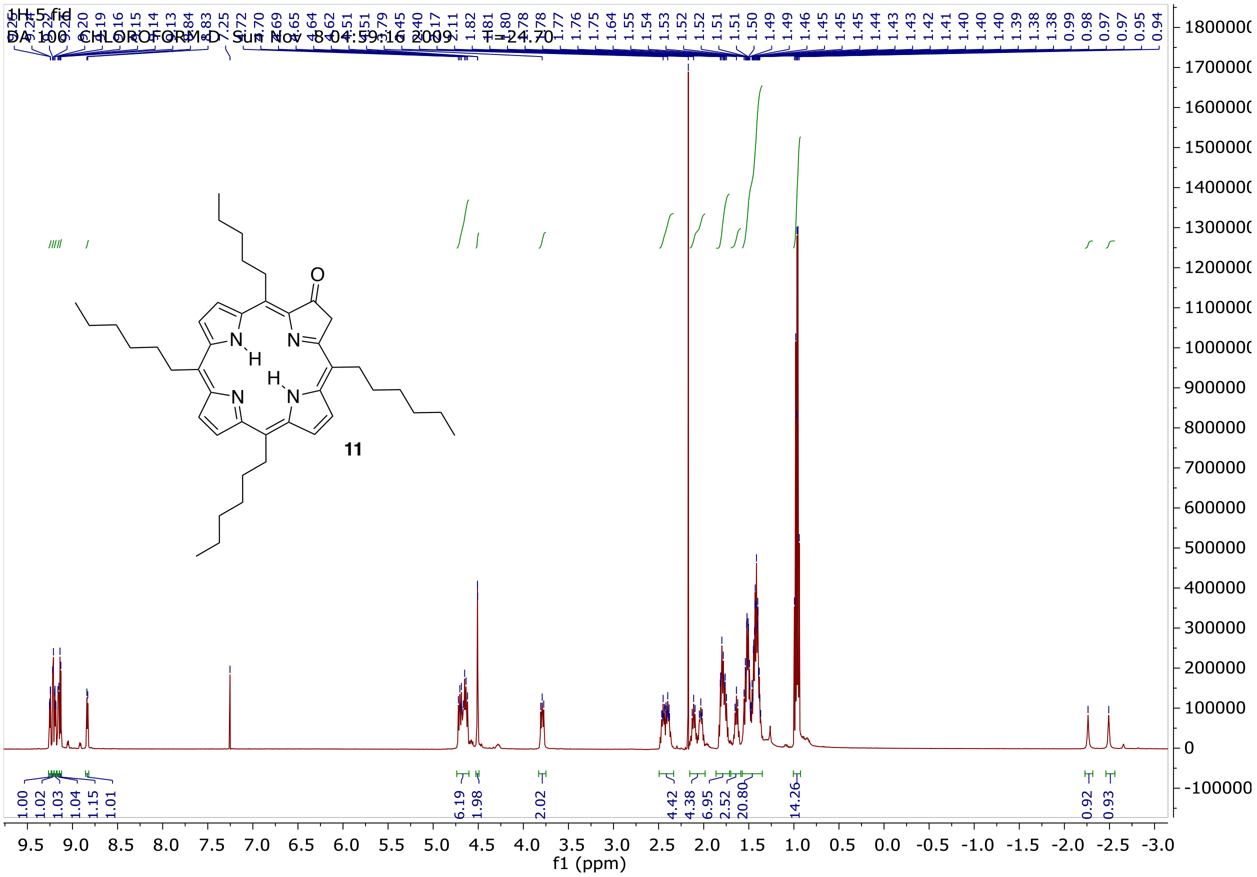

Figure S-1. ^{1}H NMR (500 MHz, $CDCl_3$) spectrum of *meso*-tetrahexylchlorin-7-one (**11**) (full spectrum, bottom, and detail, top).

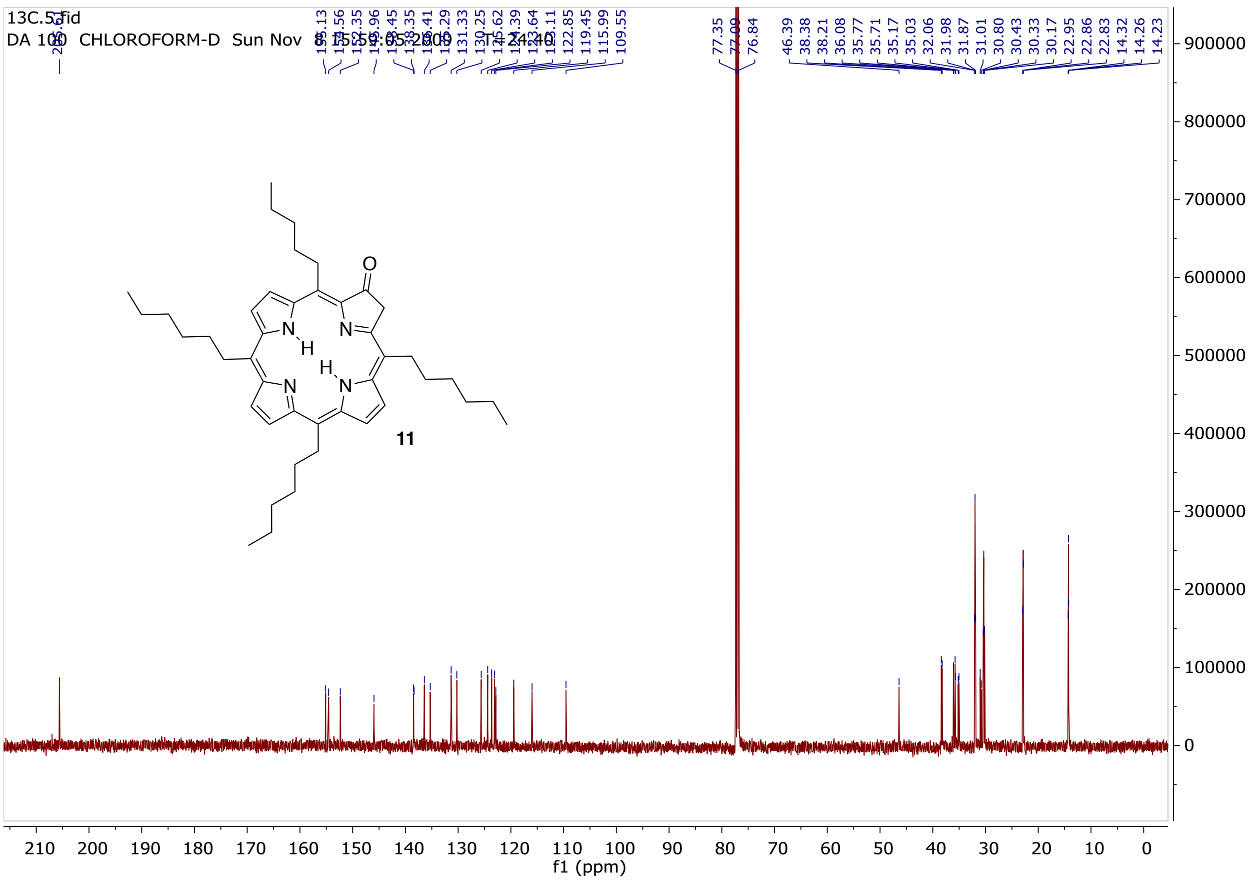

Figure S-2. ^{13}C NMR (126 MHz, $CDCl_3$) spectrum of *meso*-tetrahexylchlorin-7-one (**11**).

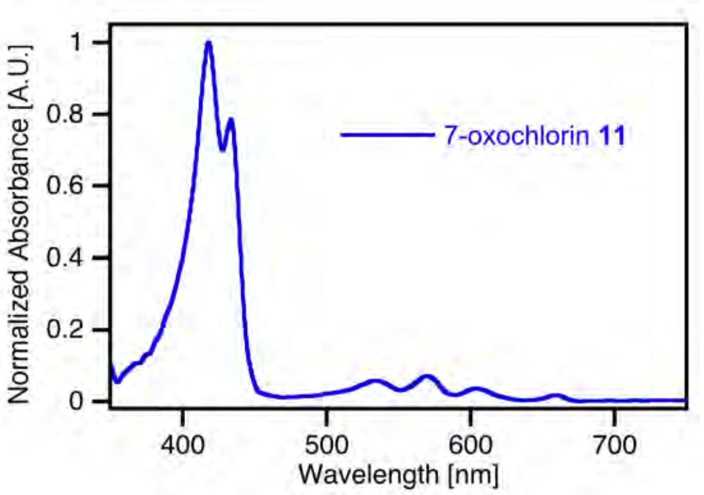

Figure S-3. UV-vis spectrum (CH_2Cl_2) of *meso-t*etrahexylchlorin-7-one (**11**).

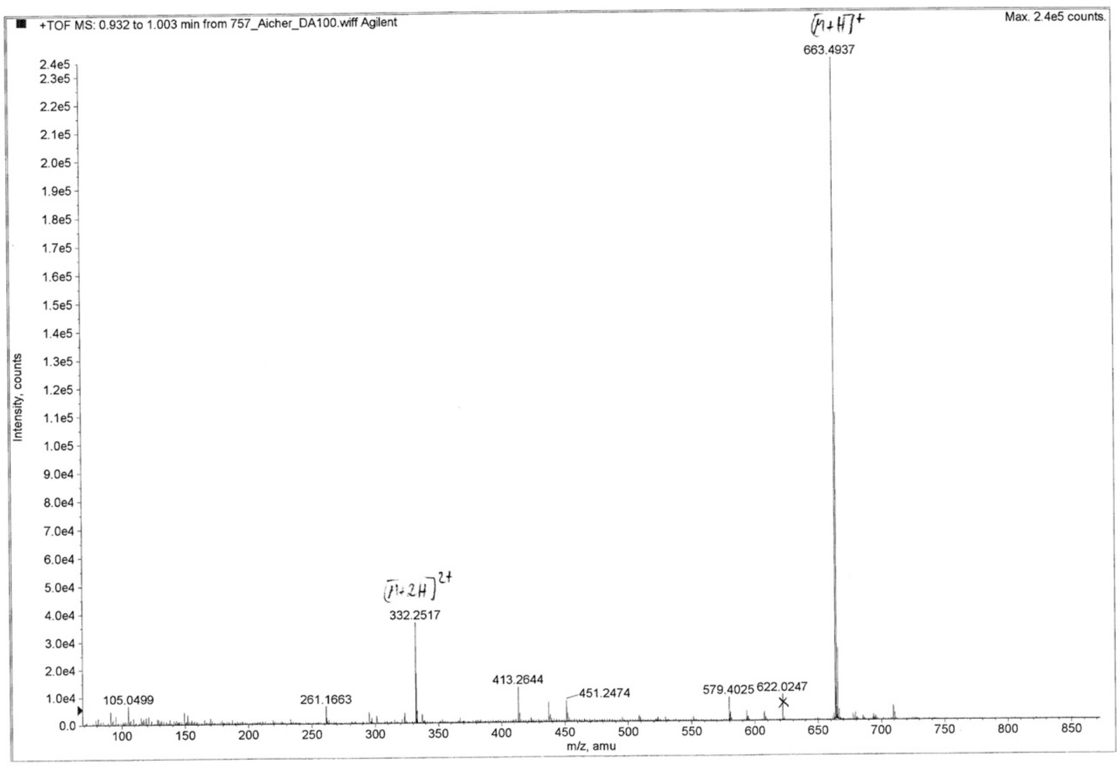

Figure S-4. HR-MS (ESI+, TOF) of *meso*-tetrahexylchlorin-7-one (**11**).

meso-Tetrahexyl-7-hydroxychlorin (12).

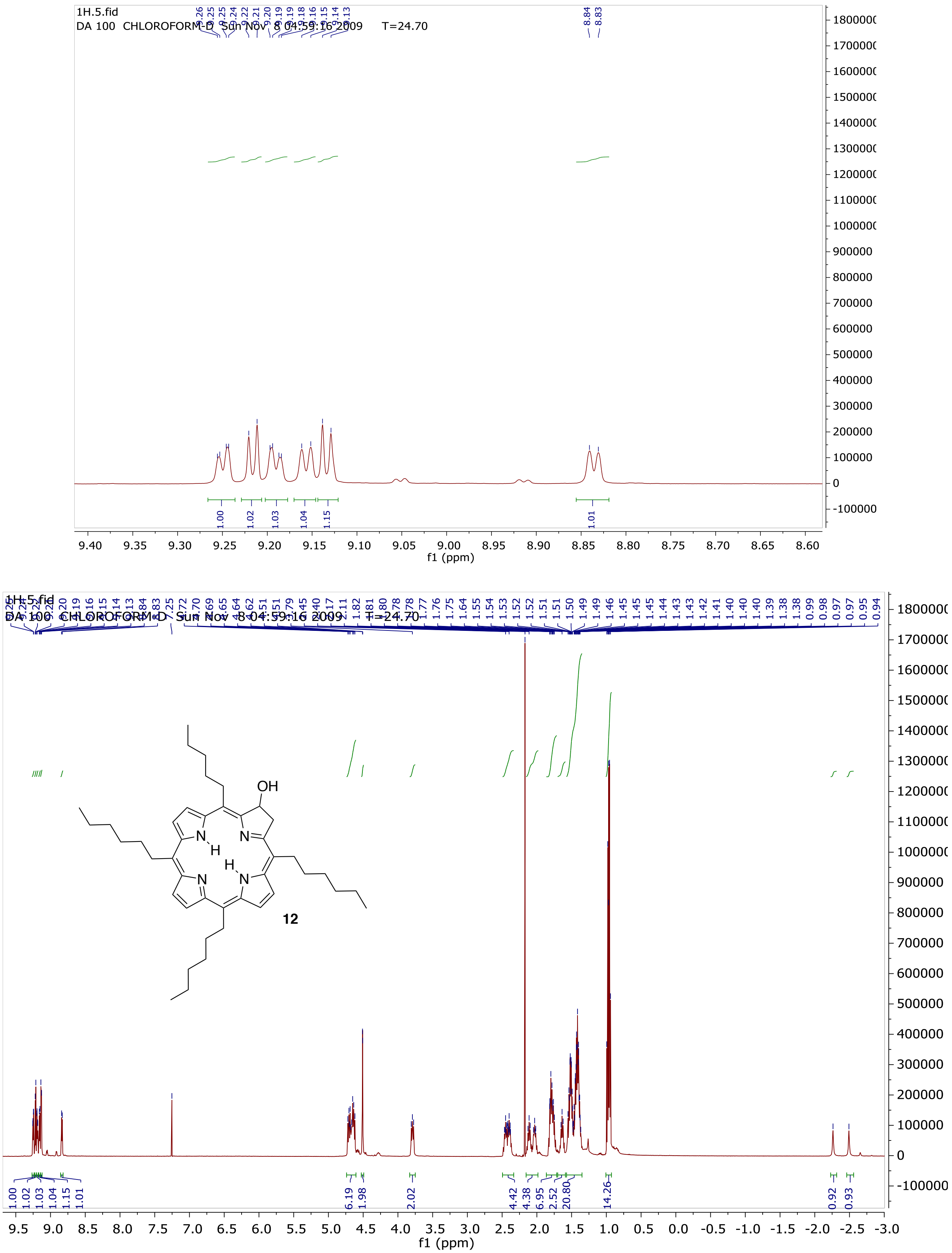

Figure S-1. ^{1}H NMR (500 MHz, $CDCl_3$) spectrum of *meso*-tetrahexyl-7-hydroxychlorin (**12**) (full spectrum, bottom, and detail, top).

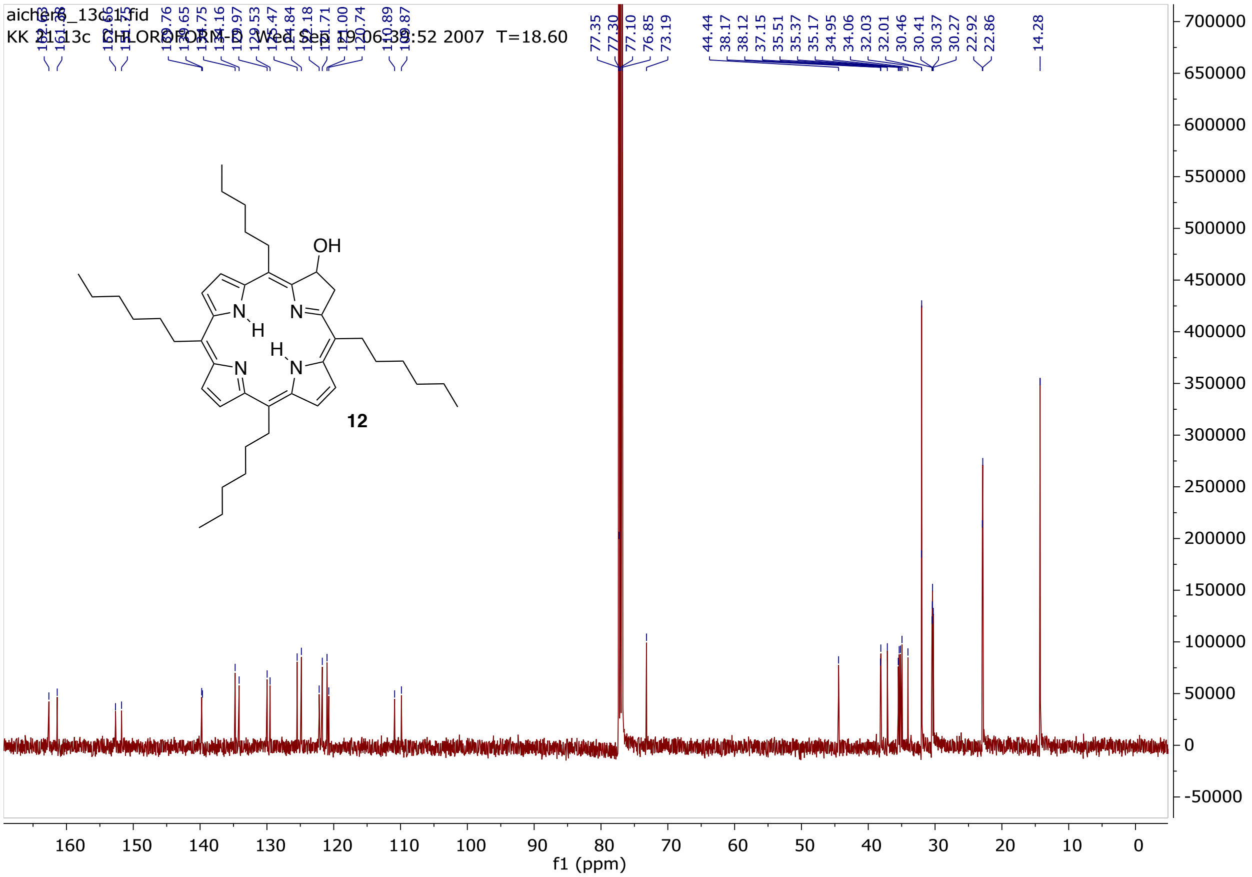

Figure S-2. ^{13}C NMR spectrum (126 MHz, $CDCl_3$) of *meso*-tetrahexyl-7-hydroxychlorin (**12**).

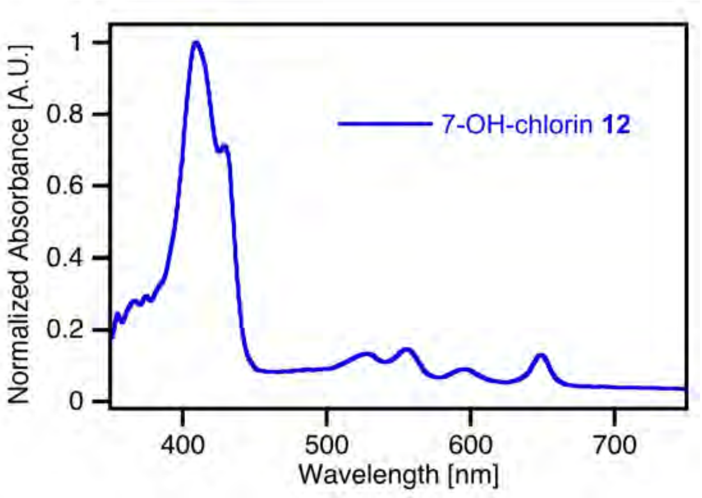

Figure S-3. UV-vis spectrum (CH_2Cl_2) of *meso*-tetrahexyl-7-hydroxychlorin (**12**).

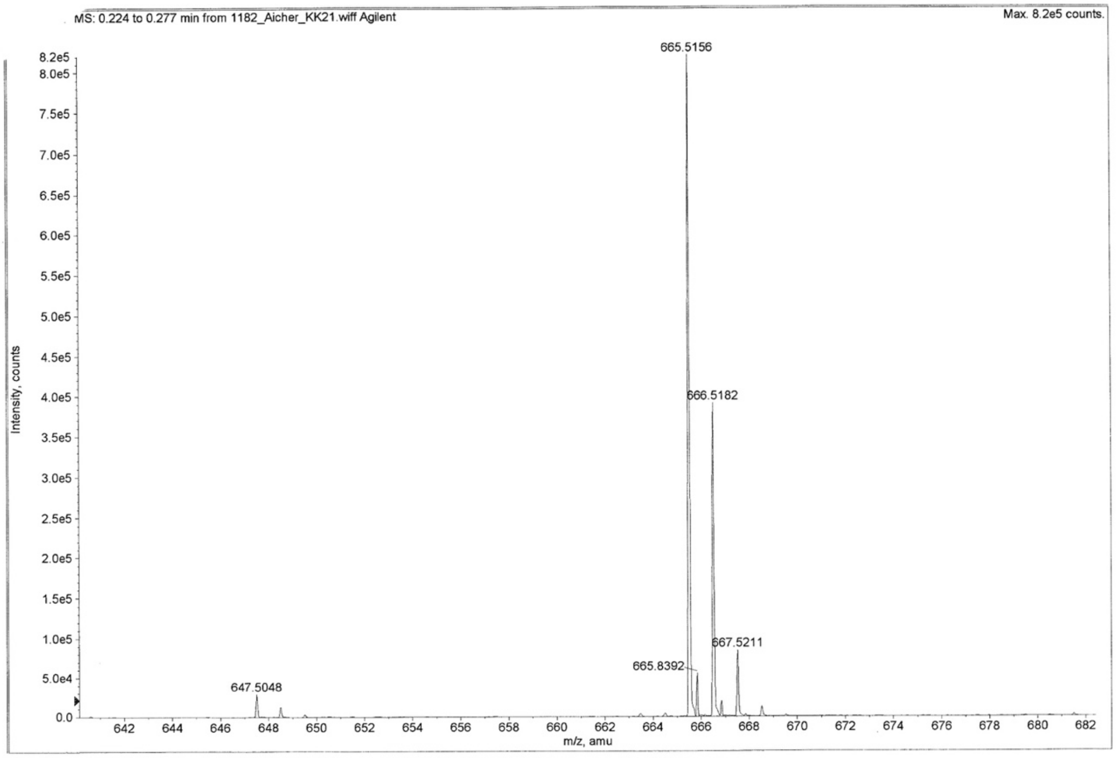

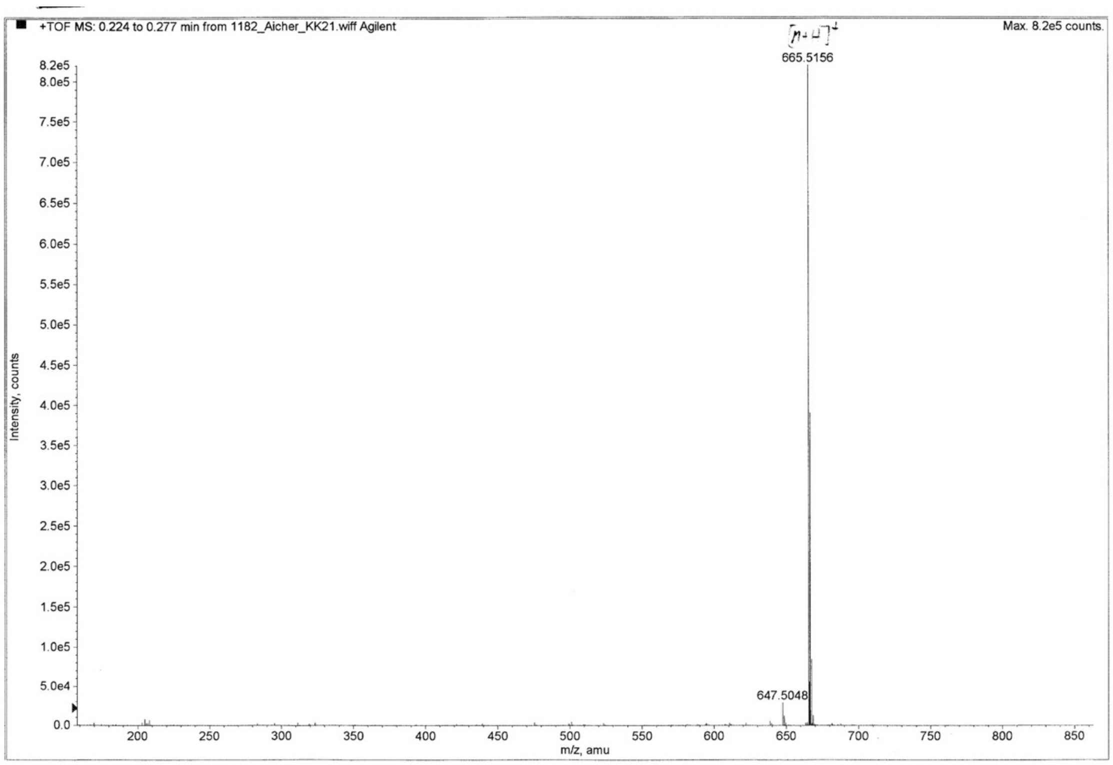

Figure S-4. Mass spectrum (ESI+, TOF) of *meso*-tetrahexyl-7-hydroxychlorin (**12**) (full and detail).

meso-Tetrahexylporpholactone (*meso*-Tetrahexyl-7-oxo-8-oxa-porphyrin) (13).

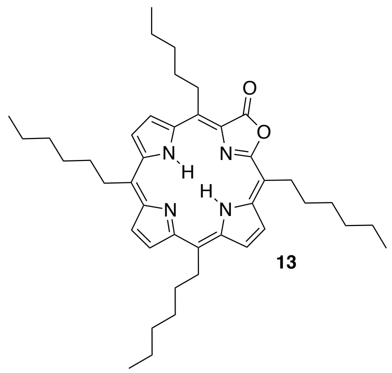

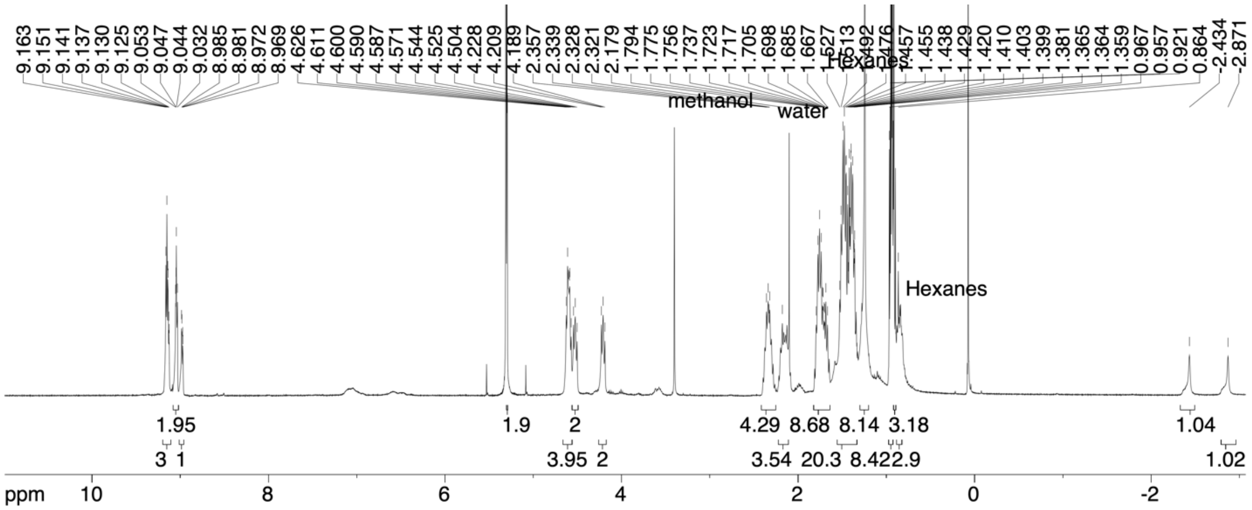

Figure S-1. ^{1}H NMR (400 MHz, $CDCl_3$) spectrum of *meso*-tetrahexylporpholactone (**13**).

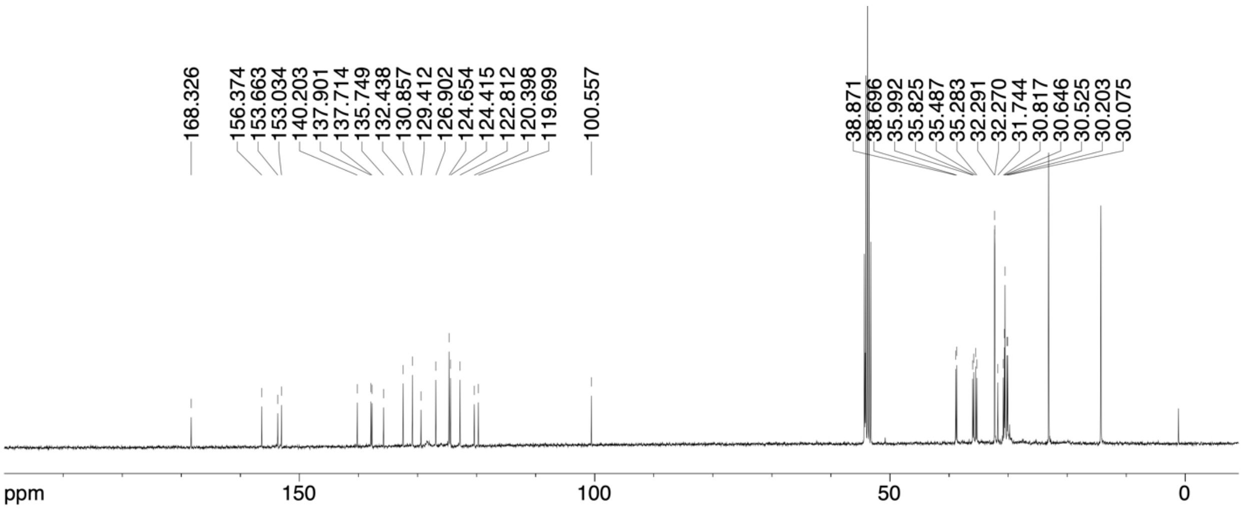

Figure S-2. ^{13}C NMR (101 MHz, $CDCl_3$) of *meso*-tetrahexylporpholactone (**13**).

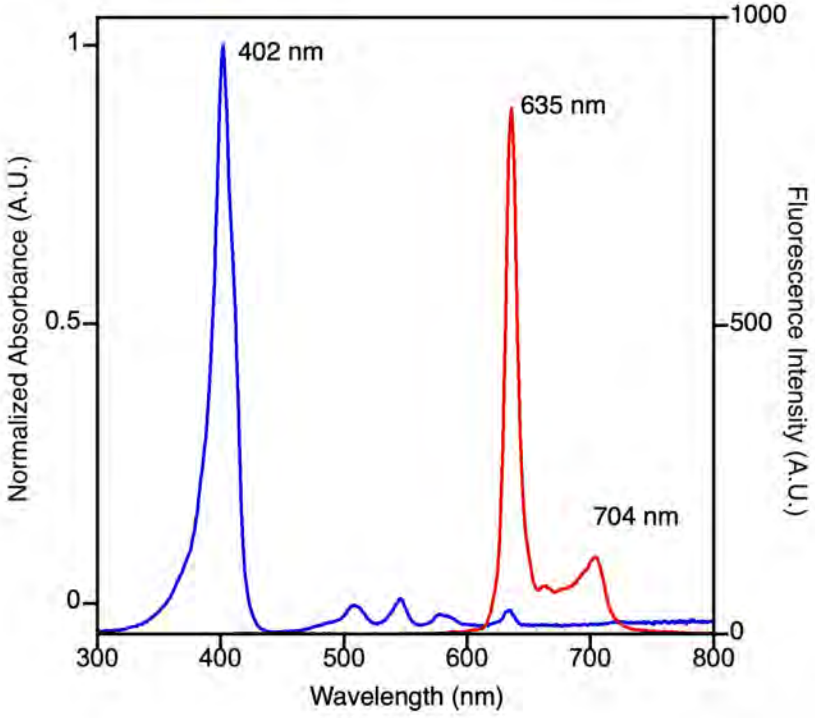

Figure S-3. UV-vis (blue) and fluorescence emission (red) spectra (CH_2Cl_2) of *meso*-tetrahexylporpholactone (**13**); $\lambda_{excitation} = \lambda_{Soret}$.

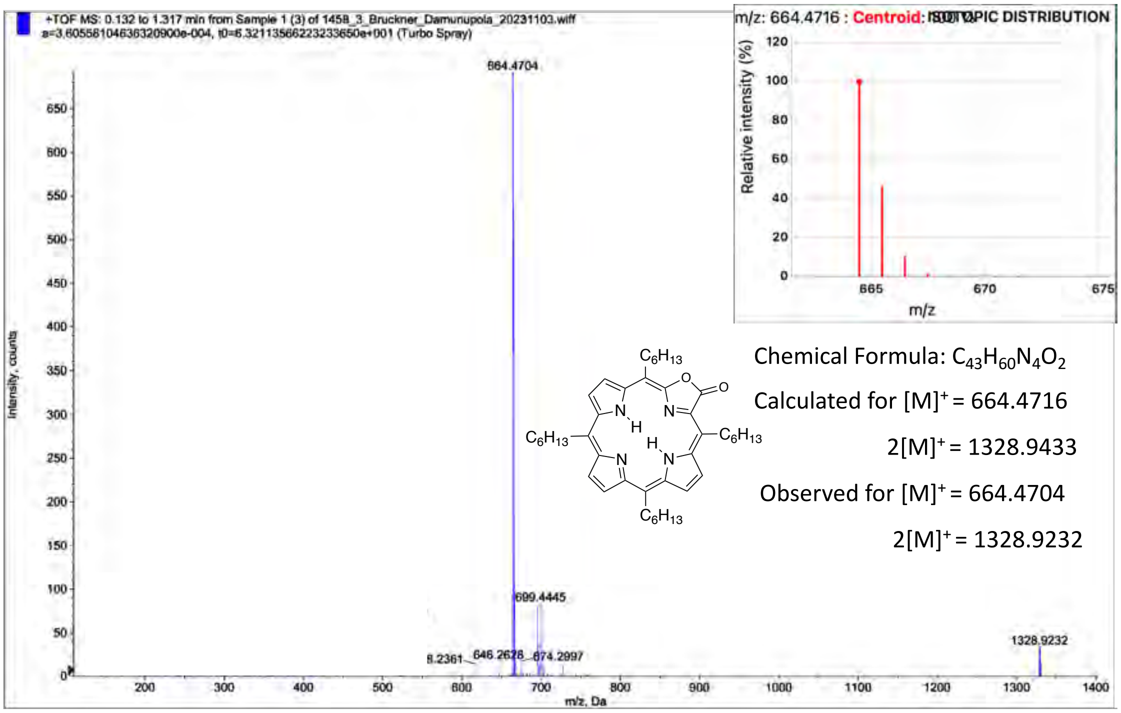

Figure S-4. HR-MS ESI$^+$ (CH_3CN, TOF) of *meso*-tetrahexylporpholactone (**13**).

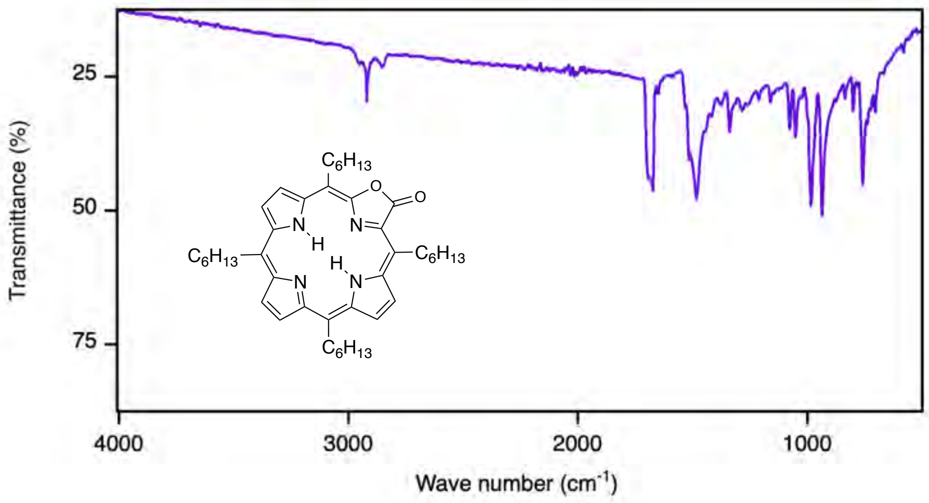

Figure S-5. FT-IR spectrum (neat, ATR) of *meso*-tetrahexylporpholactone (**13**).

5-(1'-Oxo-hexyl)-10,15,20-trihexylporphyrin (14).

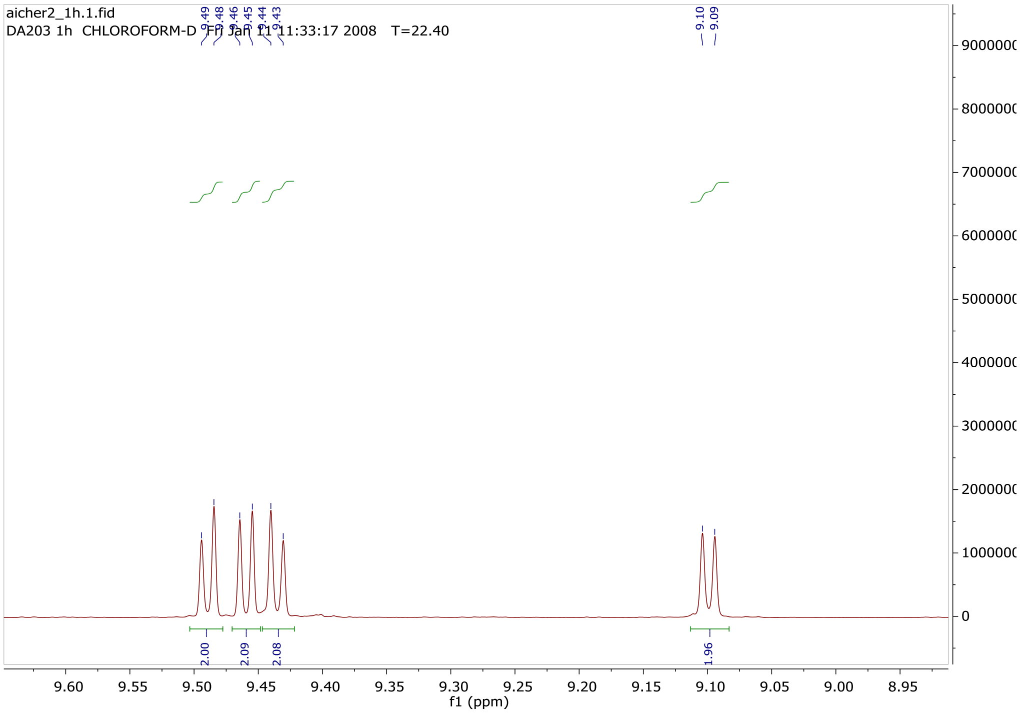

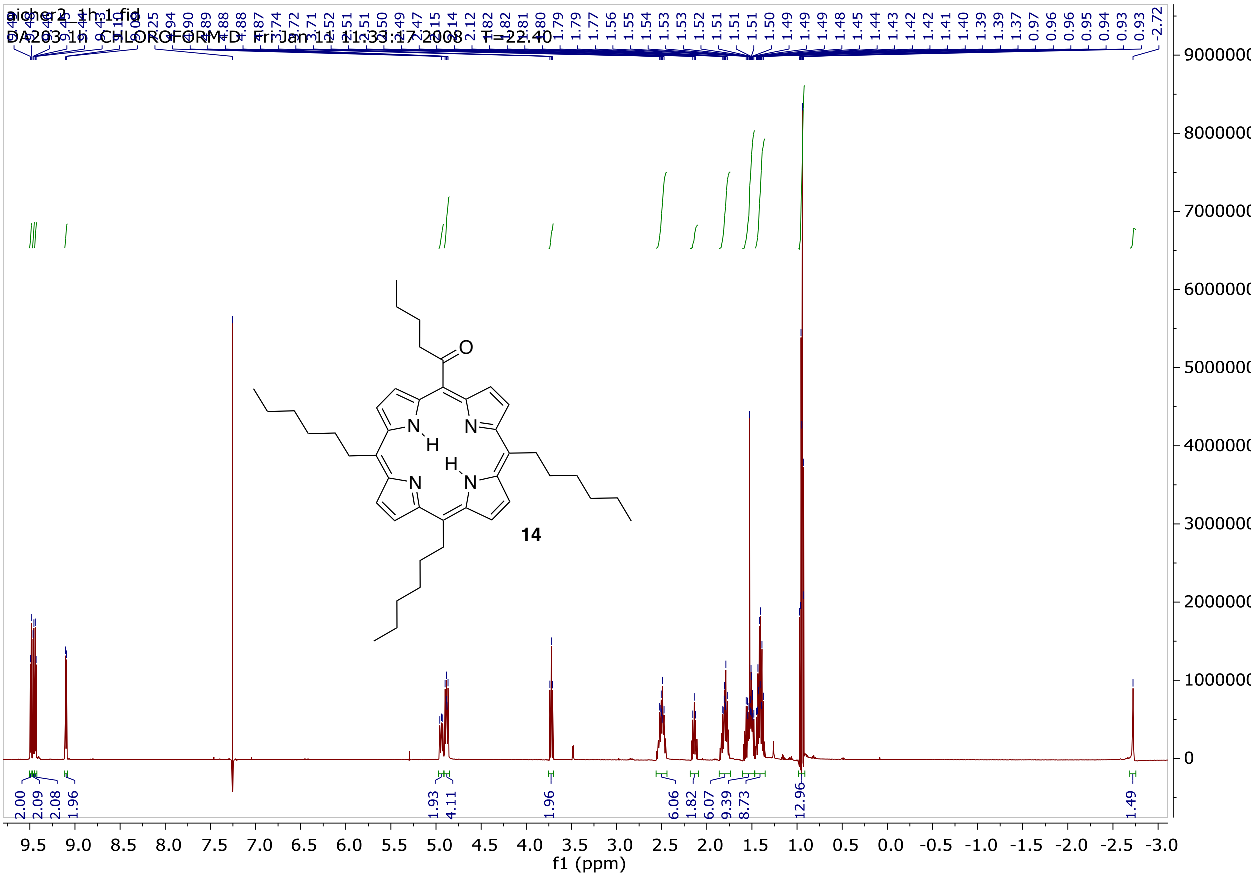

Figure S-1. ^{1}H NMR (500 MHz, $CDCl_3$) spectrum of 5-(1'-oxo-hexyl)-10,15,20-trihexylporphyrin (**14**)

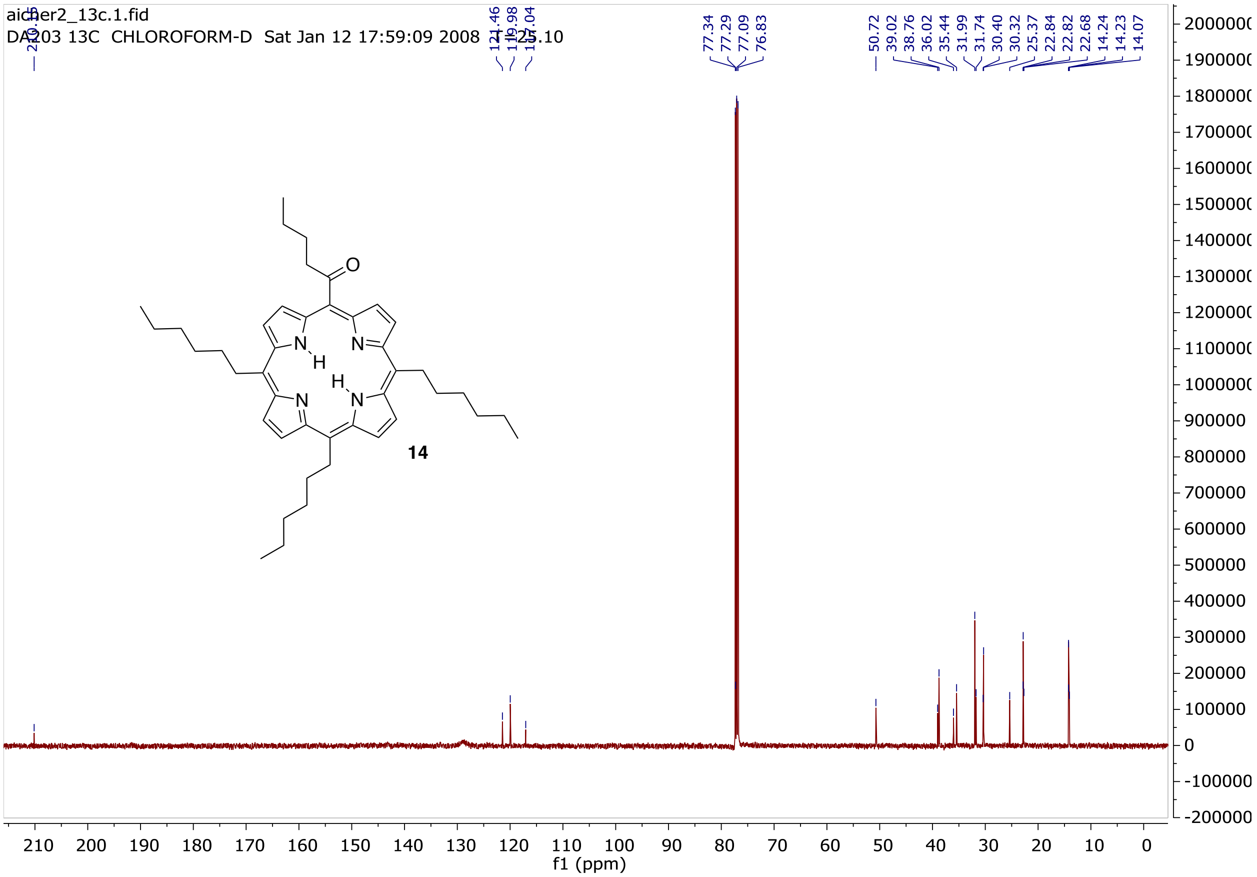

Figure S-2. 13C NMR (126 MHz, $CDCl_3$) of 5-(1'-oxo-hexyl)-10,15,20-trihexylporphyrin (**14**)

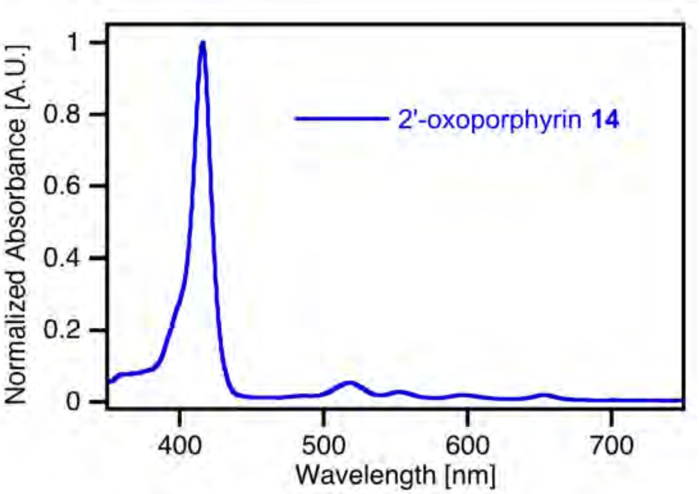

Figure S-3. UV-vis spectrum (CH_2Cl_2) of 5-(1'-oxo-hexyl)-10,15,20-trihexylporphyrin (**14**).

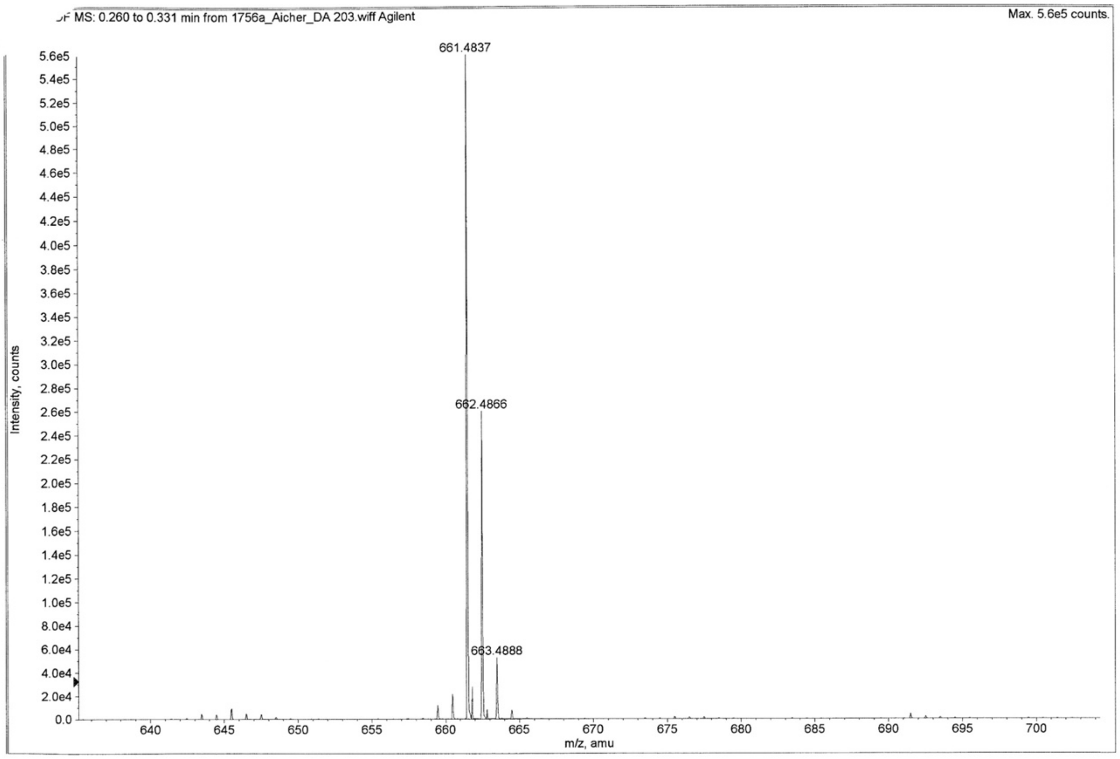

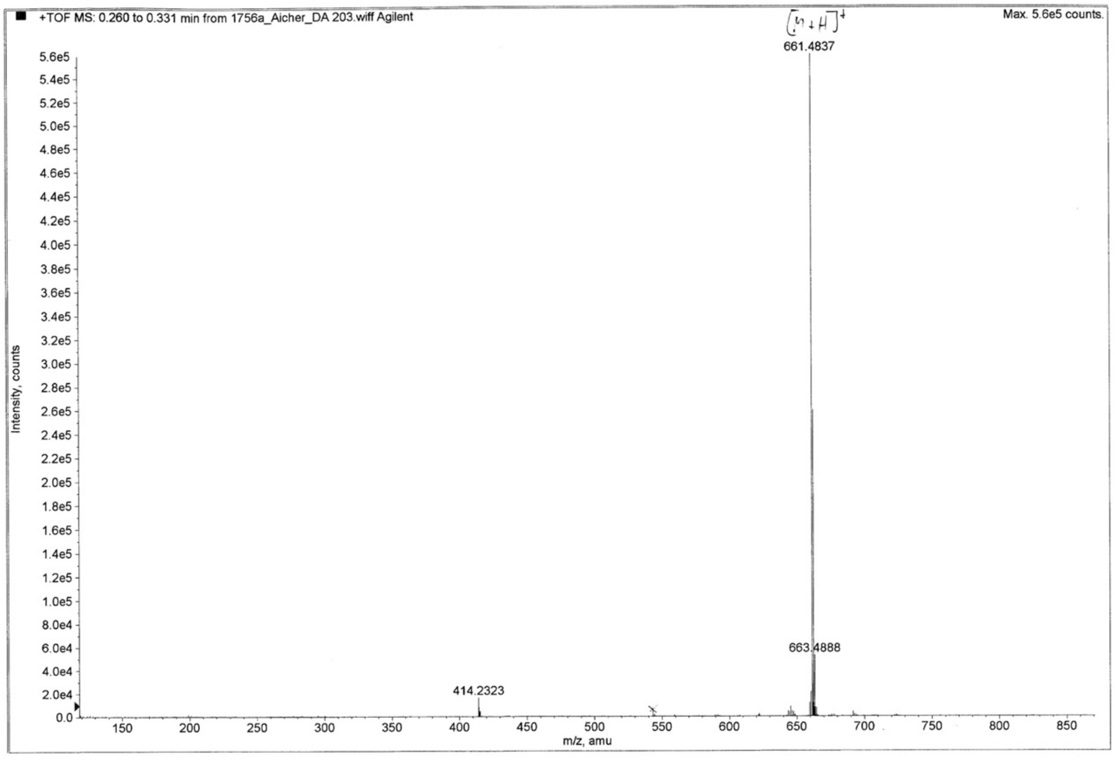

Figure S-4. Mass spec of 5-(1'-oxo-hexyl)-10,15,20-trihexylporphyrin (**14**)

5,15-Dihexyl-3-oxo-2-oxa-porphyrin) (16A)

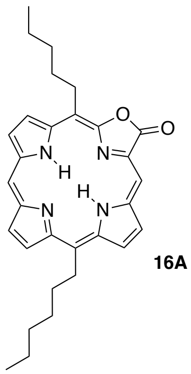

16A

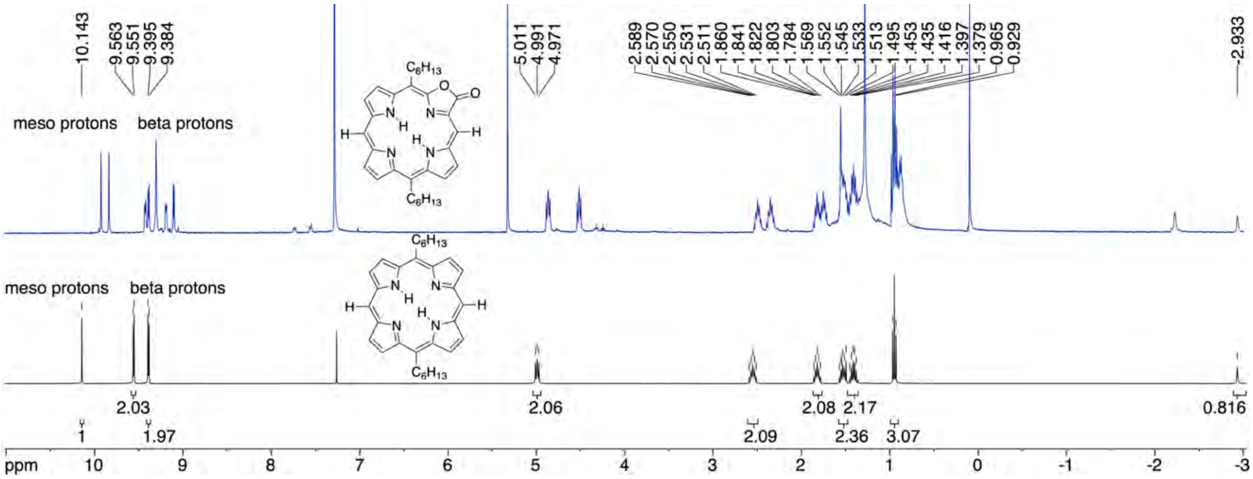

Figure S-1. ^{1}H NMR (400 MHz, $CDCl_3$) spectrum of 5,15-dihexyl-3-oxo-2-oxa-porphyrin) (**16A**) in comparison to that of the parent porphyrin **15**.

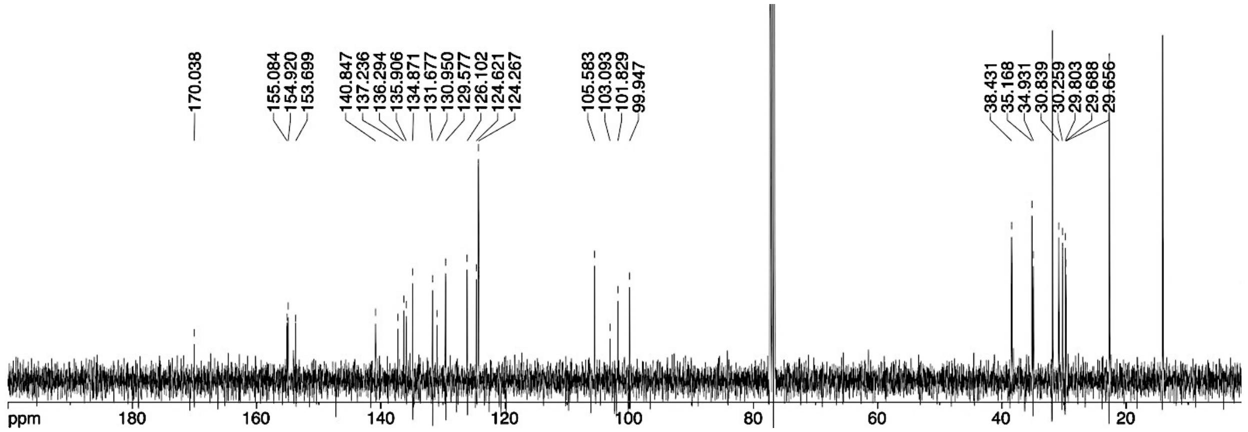

Figure S-2. {^{1}H}^{13}C NMR spectrum (101 MHz, $CDCl_3$) of *meso*-dihexyl-3-oxo-2-oxa-porphyrin) (**16A**)

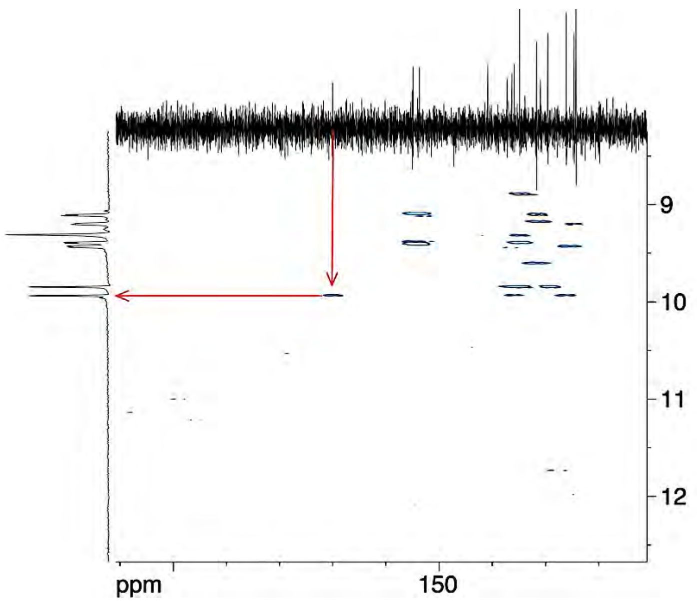

Figure S-3. $^1H,^{13}C$ HMBC NMR spectrum (400, 100 MHz, $CDCl_3$) of *meso*-dihexyl-3-oxo-2-oxa-porphyrin) (**16A**)

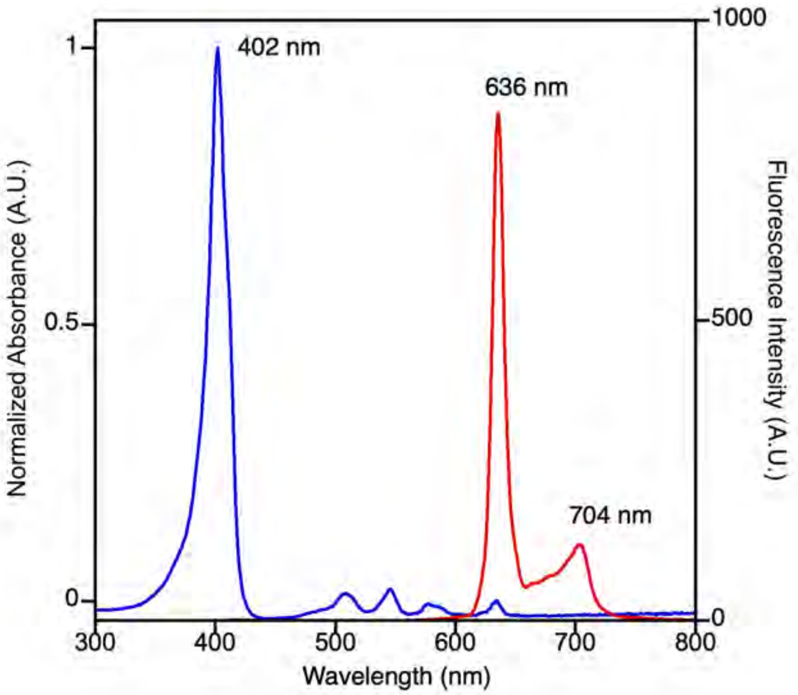

Figure S-4. UV-vis (blue) and fluorescence emission spectra (CH_2Cl_2) of *meso*-dihexyl-3-oxo-2-oxa-porphyrin) (**16A**); $\lambda_{excitation} = \lambda_{Soret}$.

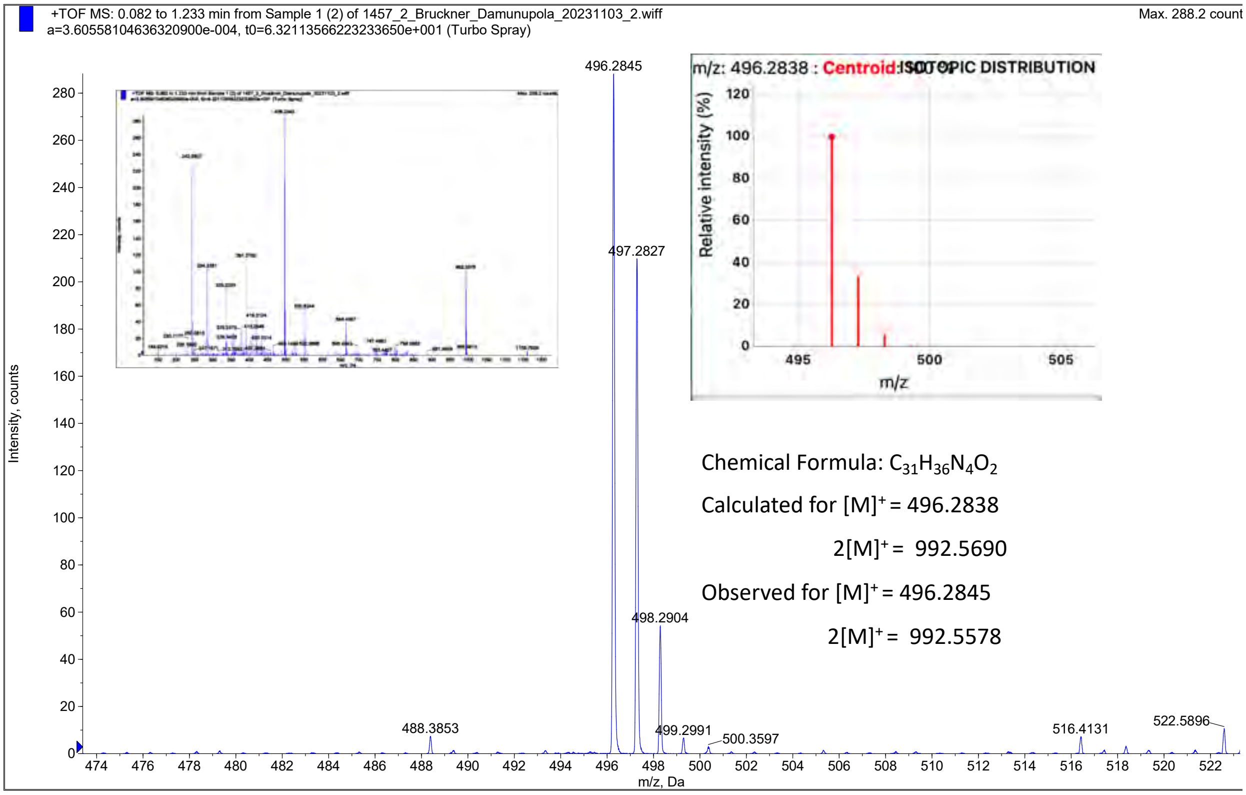

Figure S-5. HR-MS spectrum (ESI+, CH_3CN, TOF) of *meso*-dihexyl-3-oxo-2-oxa-porphyrin) (**16A**).

5,15-Dihexyl-7-oxo-8-oxa-porphyrin) (16B).

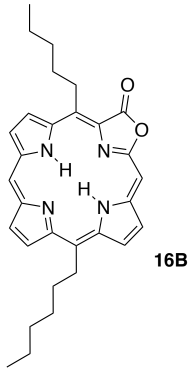

16B

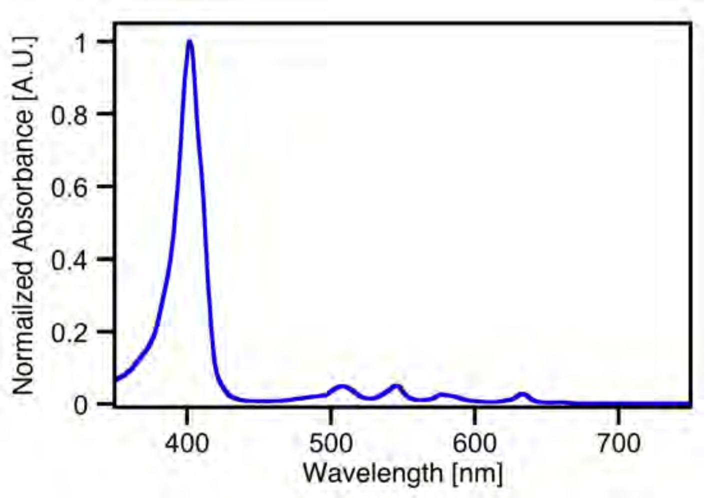

Figure S-1. UV-vis spectrum (CH_2Cl_2) of *meso*-dihexyl-7-oxo-8-oxa-porphyrin) (**16B**).

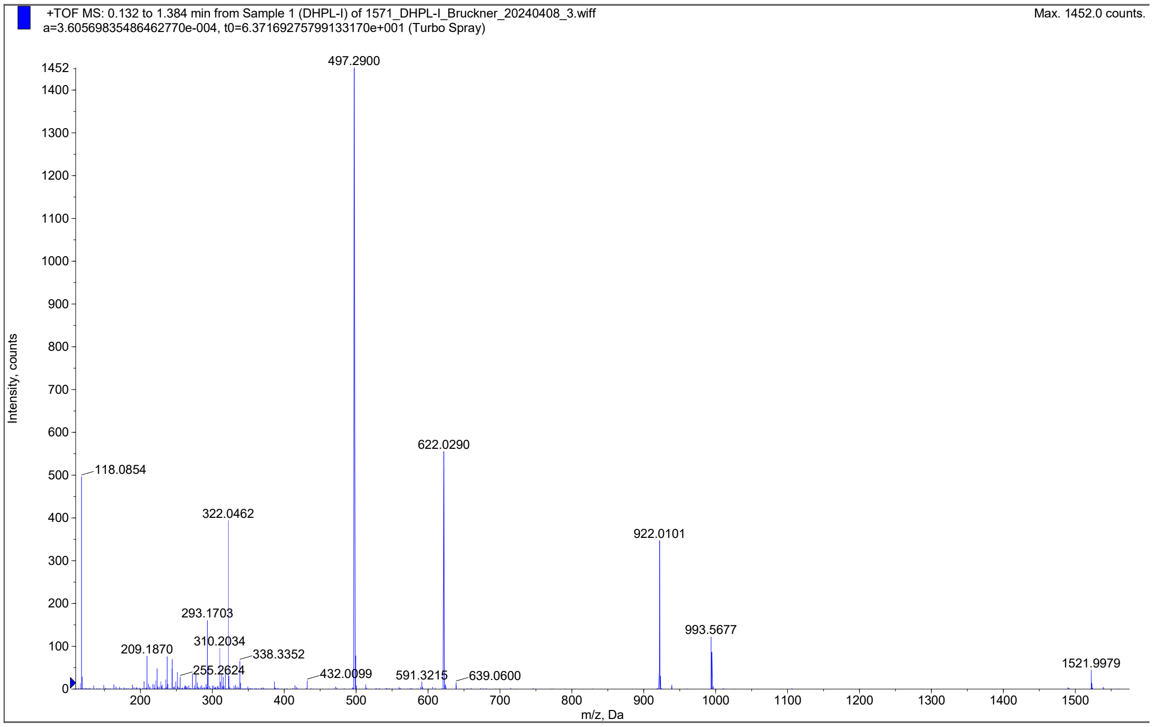

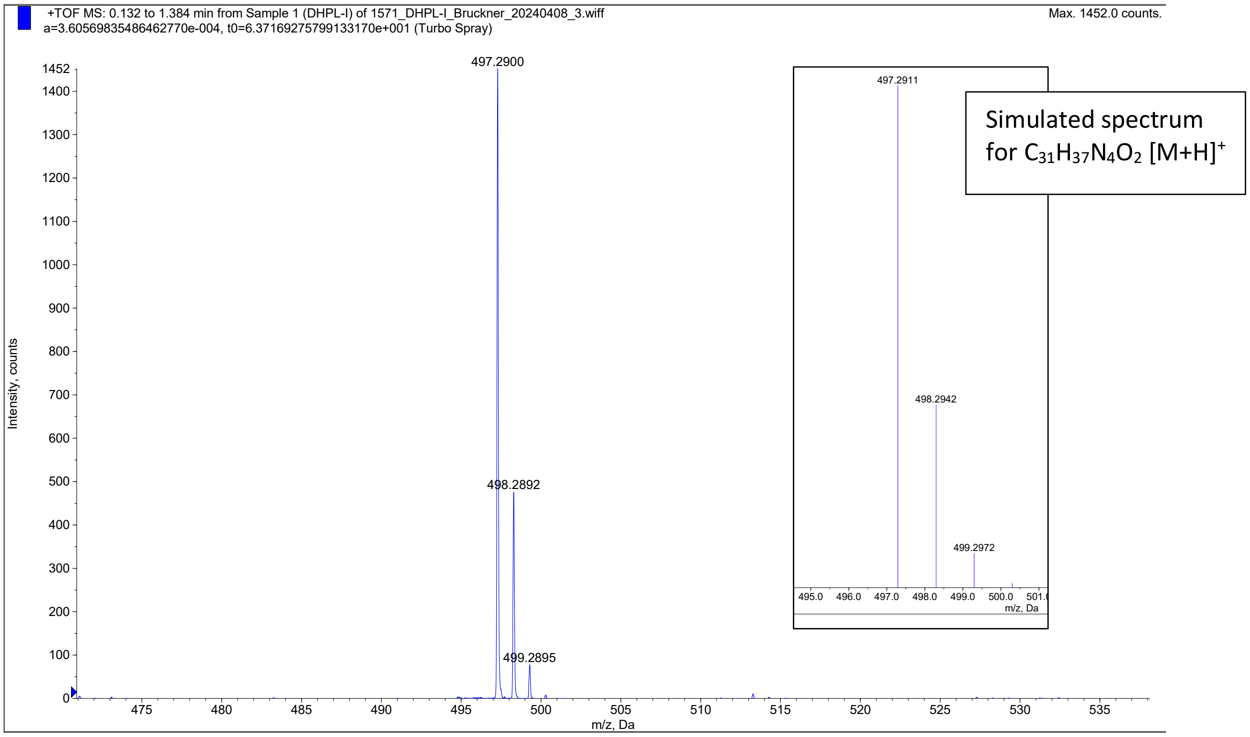

Figure S-2. HR-MS (ESI+, CH_3OH, TOF) of *meso*-dihexyl-7-oxo-8-oxa-porphyrin) (**16B**); full spectrum to, expansion with simulation, bottom.

meso-Tetrahexyl-7,8-*trans*-dihydroxychlorin (17).

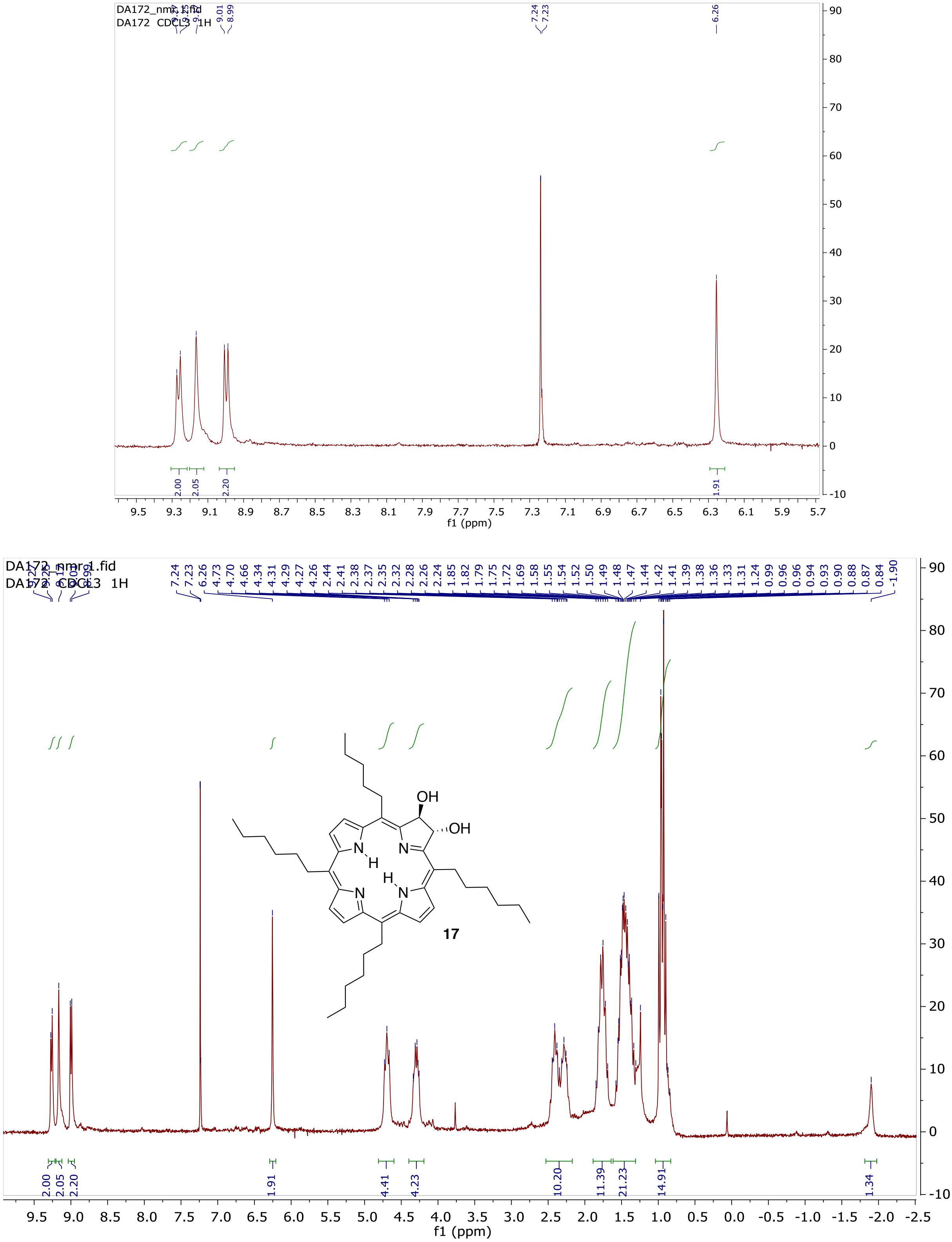

Figure S-1. ^{1}H NMR (250 MHz, $CDCl_3$) spectrum of *meso*-tetrahexyl-7,8-*trans*-dihydroxychlorin (**17**).

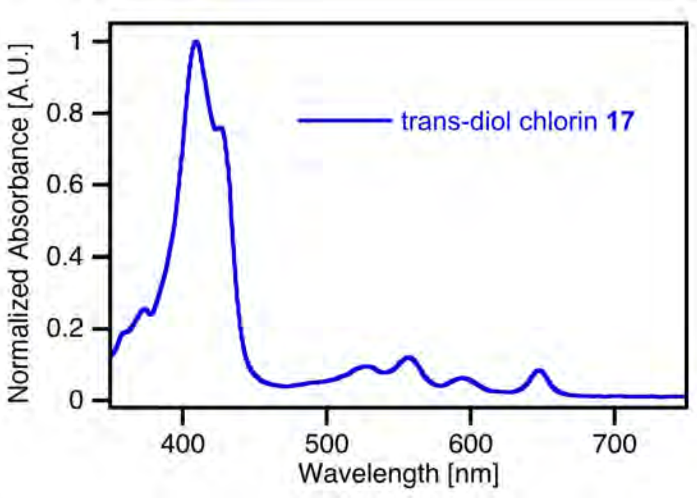

Figure S-2. UV-vis spectrum (CH_2Cl_2) of *meso*-tetrahexyl-7,8-*trans*-dihydroxychlorin (**17**).

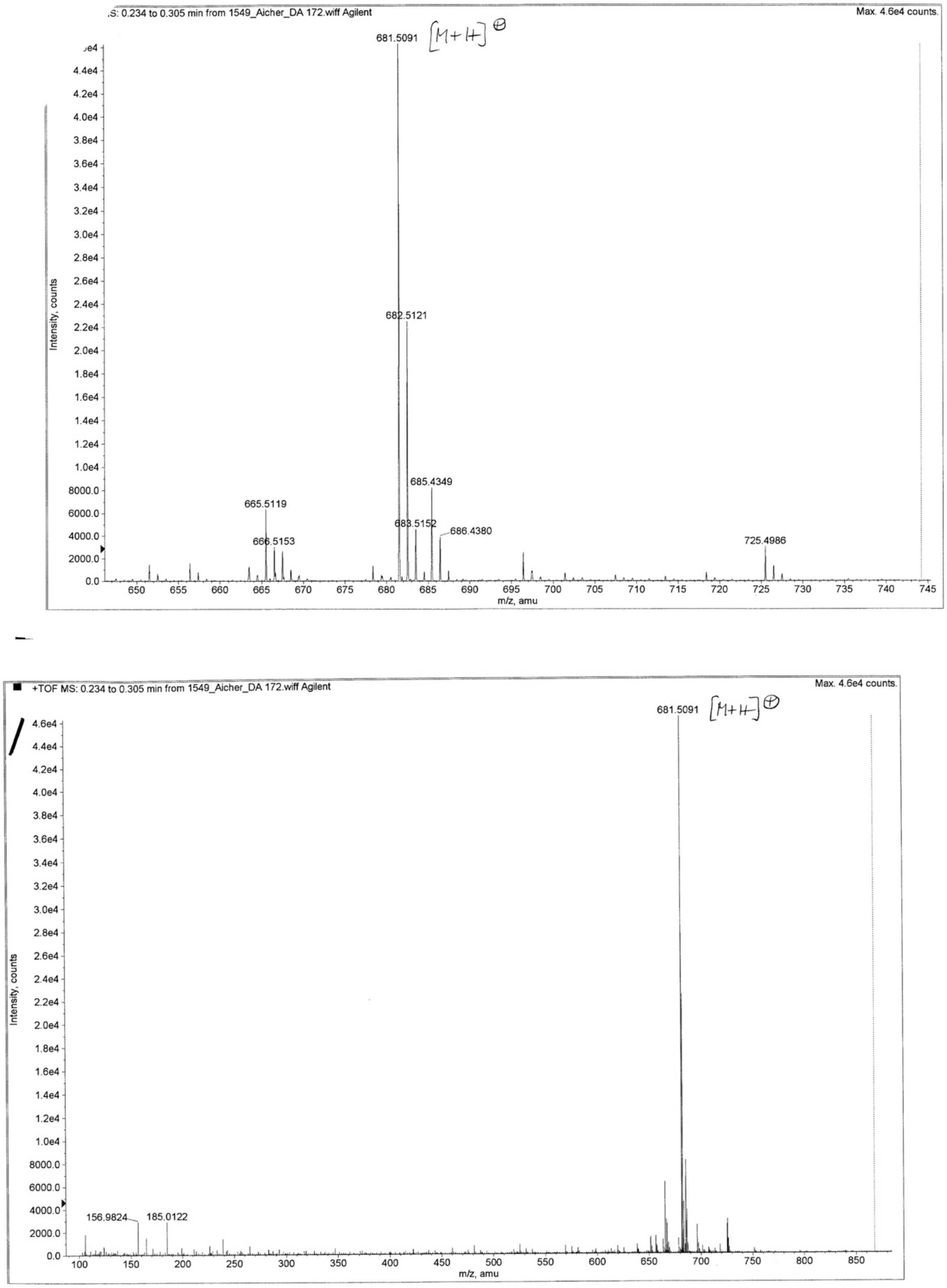

Figure S-3. HR-MS (ESI+, TOF) of *meso*-tetrahexyl-7,8-*trans*-dihydroxychlorin (**17**) (full and detail).

meso-Tetrahexyl-8-hydroxy-8-methyl-chlorin-7-one (18)

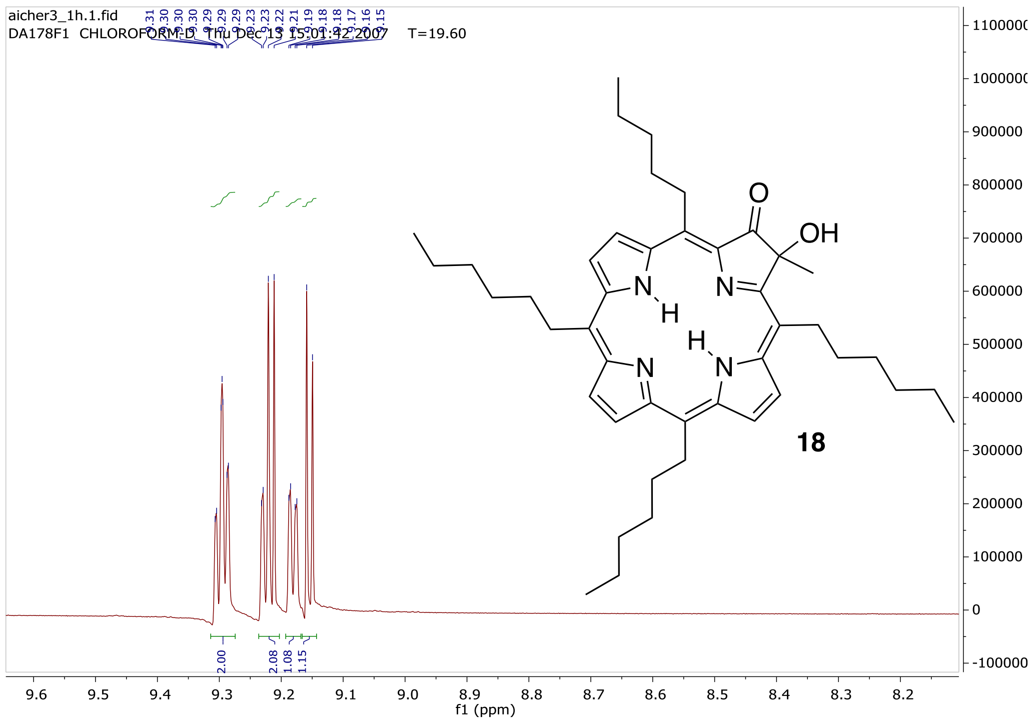

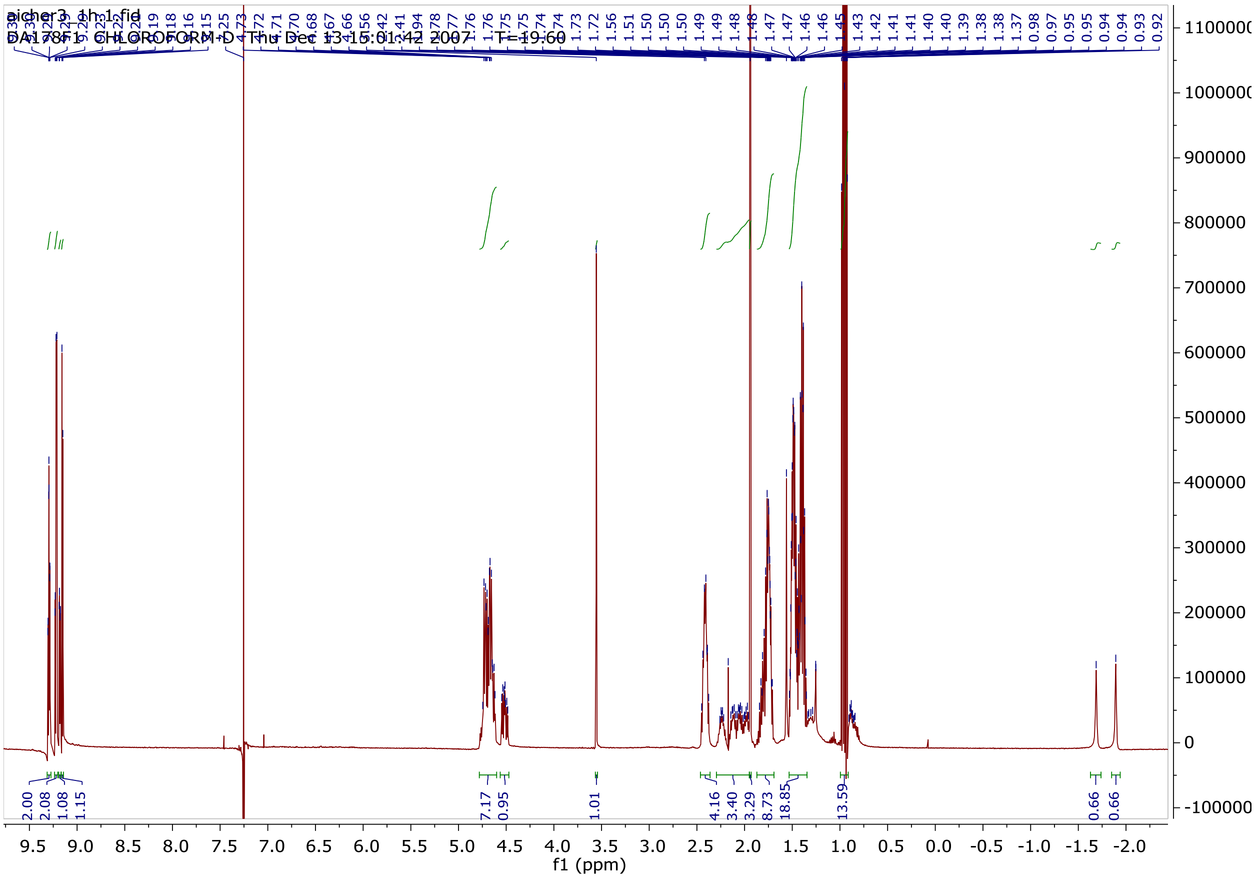

Figure S-1. ^{1}H NMR (500 MHz, $CDCl_3$) spectrum of *meso*-tetrahexyl-8-hydroxy-8-methyl-chlorin-7-one (**18**).

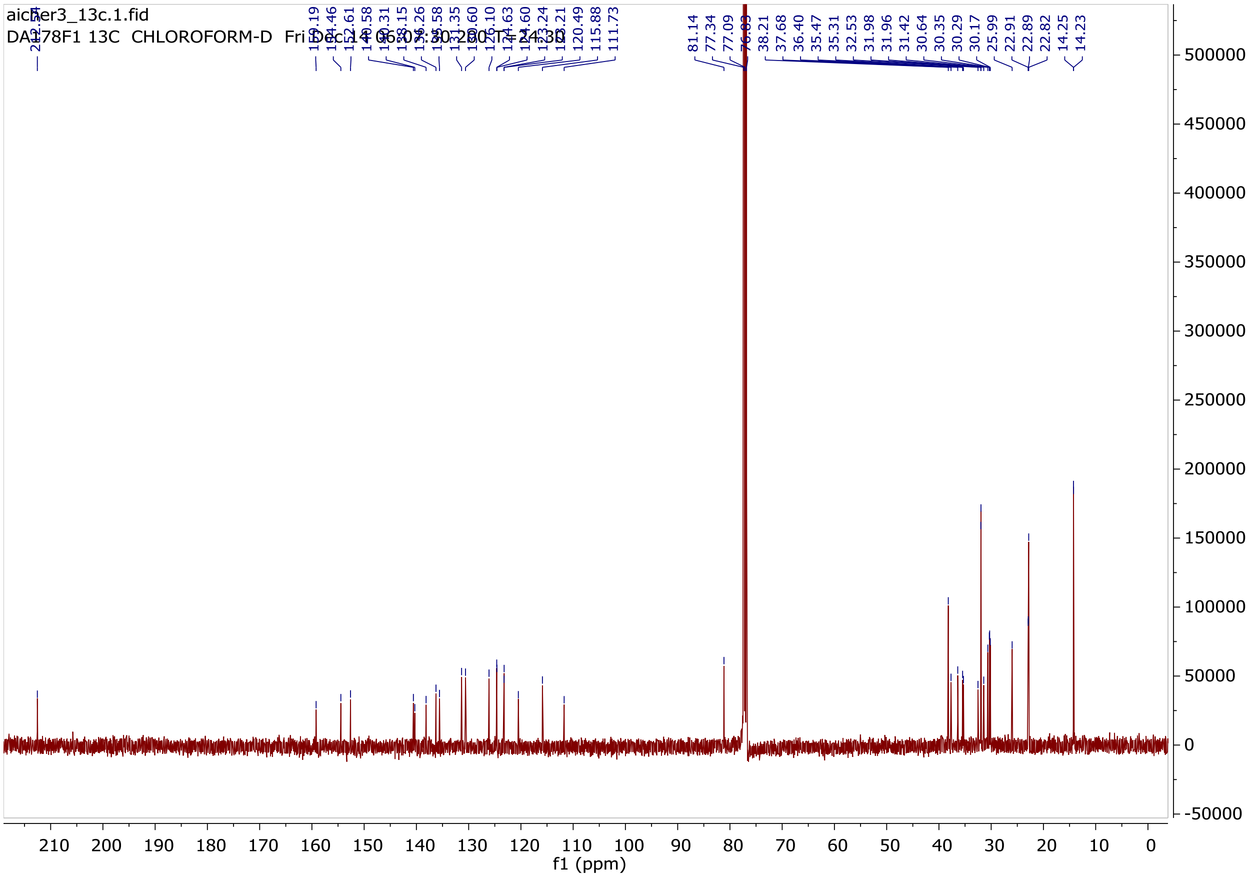

Figure S-2. 13C NMR (126 MHz, $CDCl_3$) of *meso*-tetrahexyl-8-hydroxy-8-methyl-chlorin-7-one (**18**).

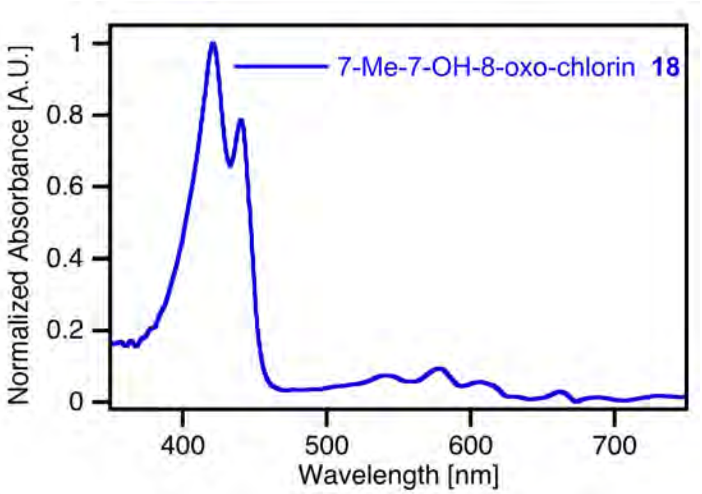

Figure S-3. UV-vis spectrum (CH_2Cl_2) of *meso*-tetrahexyl-8-hydroxy-8-methyl-chlorin-7-one (**18**).

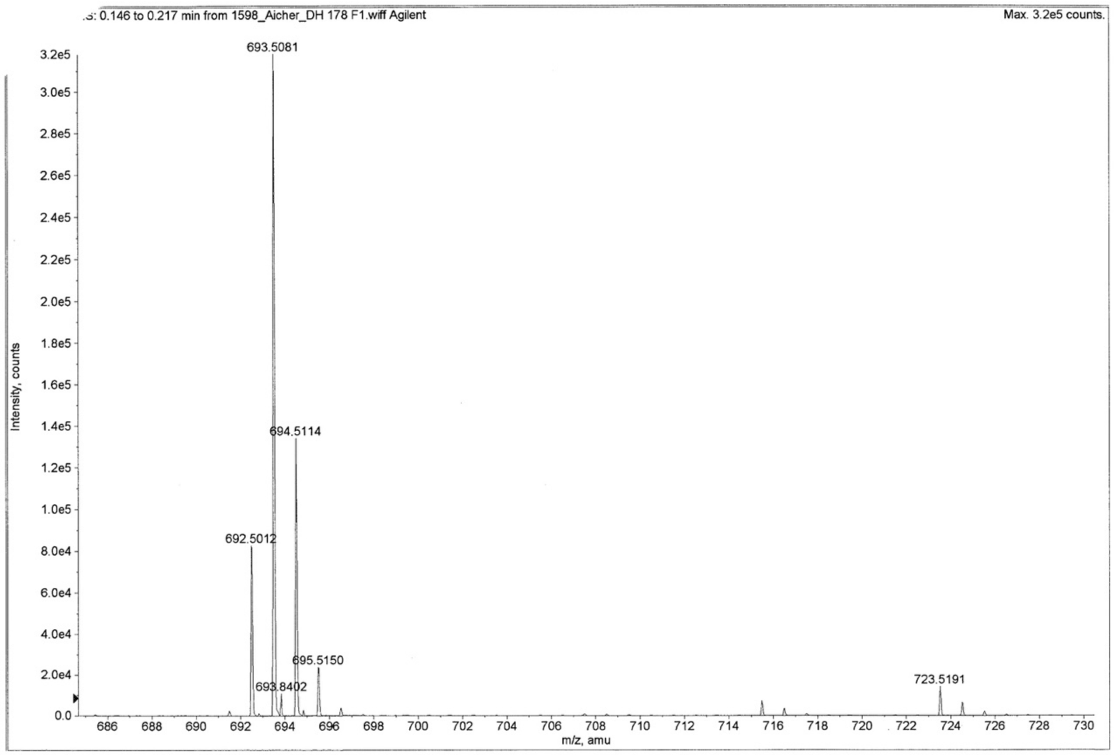

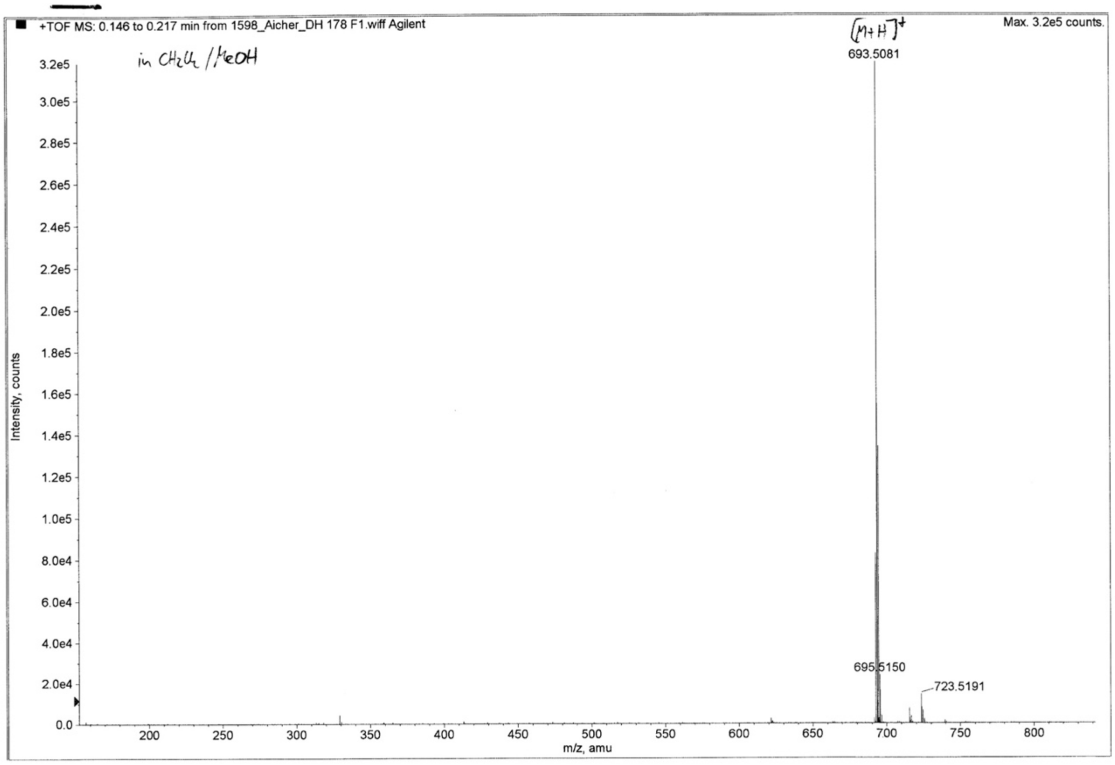

Figure S-4. Mass spec of *meso*-tetrahexyl-8-hydroxy-8-methyl-chlorin-7-one (**18**).

meso-Tetrahexyl-7,8-dihydroxy-7,8-dimethyl-chlorin (19).

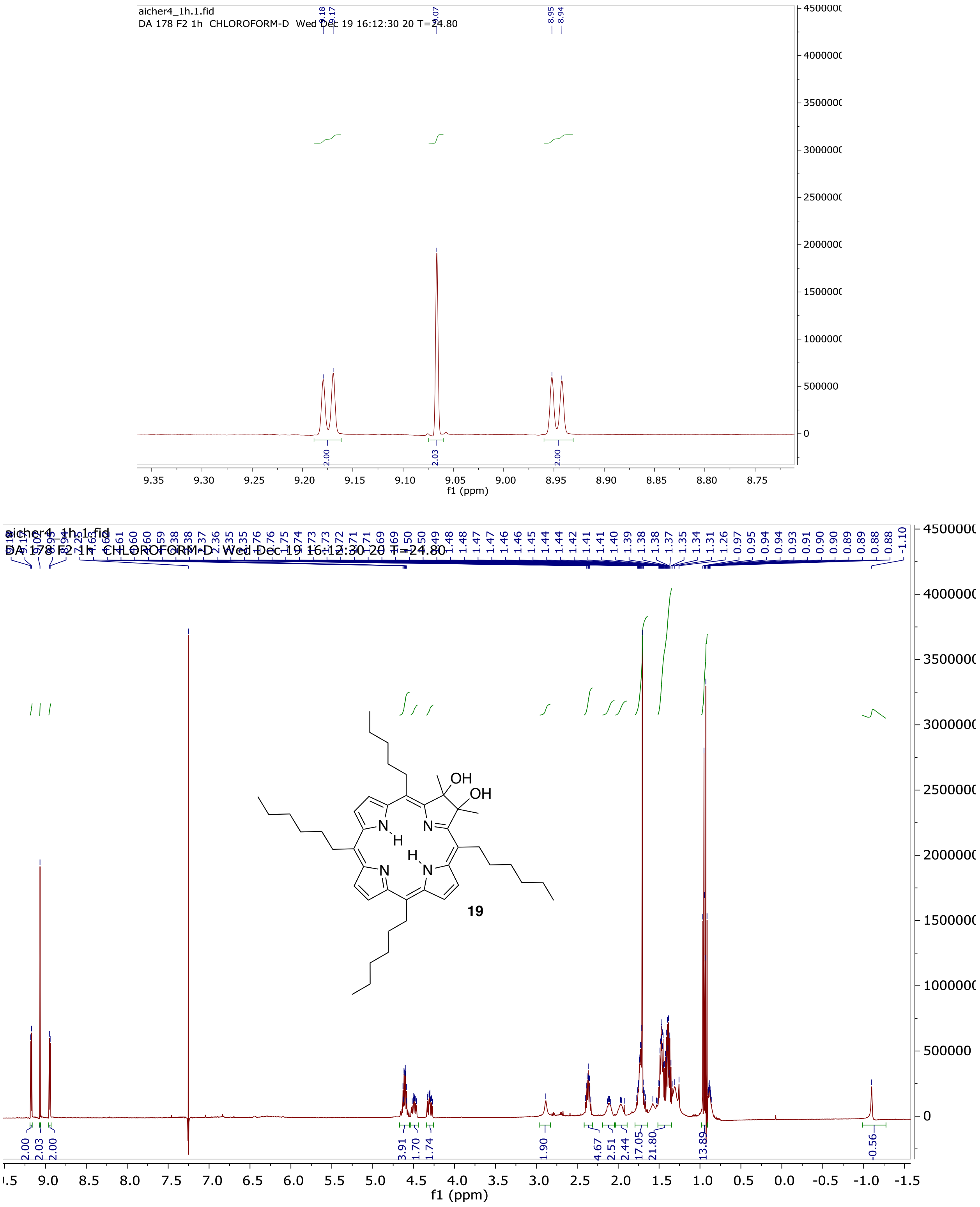

Figure S-1. ^{1}H NMR (500 MHz, $CDCl_3$) spectrum of *meso*-tetrahexyl-7,8-dihydroxy-7,8-dimethyl-chlorin (**19**).

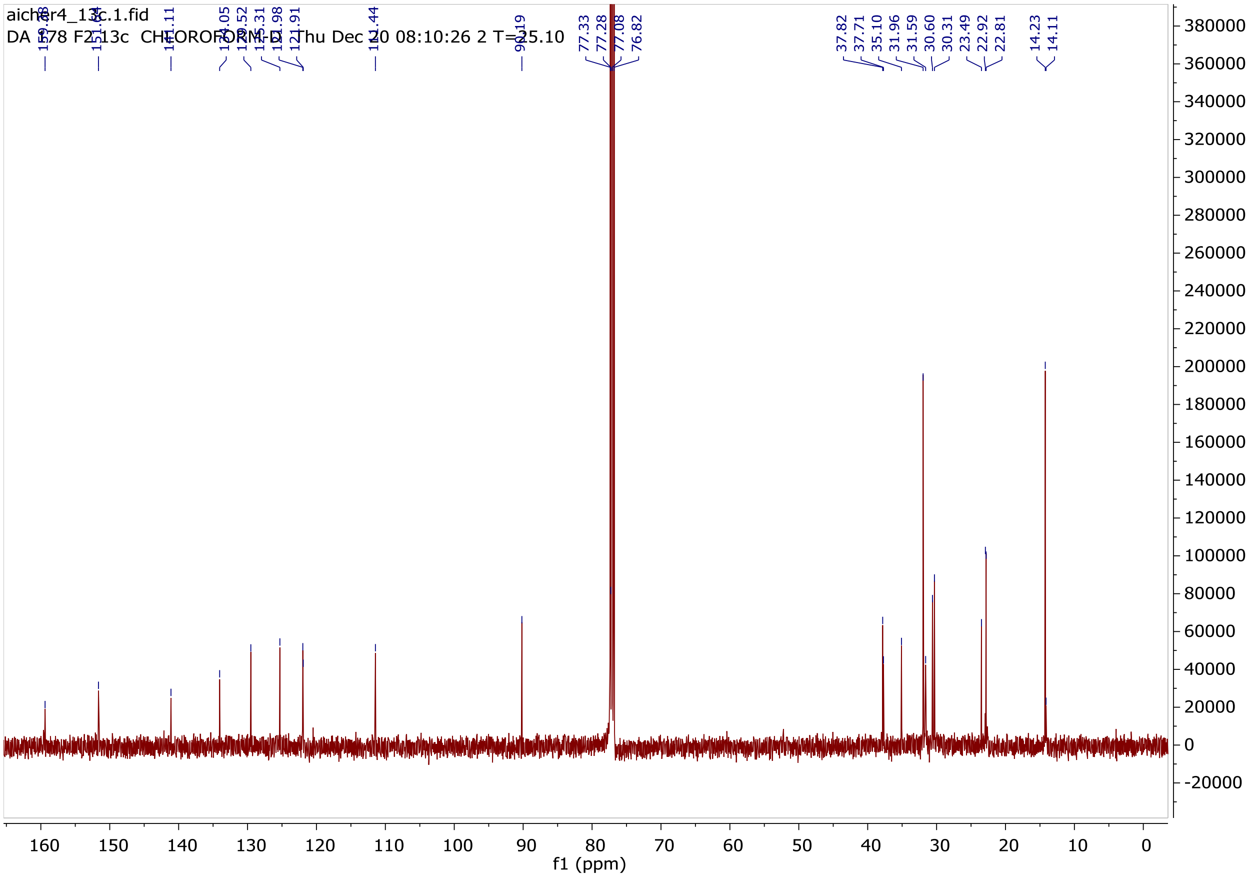

Figure S-2. 13C NMR (126 MHz, $CDCl_3$) of *meso*-tetrahexyl-7,8-dihydroxy-7,8-dimethyl-chlorin (**19**).

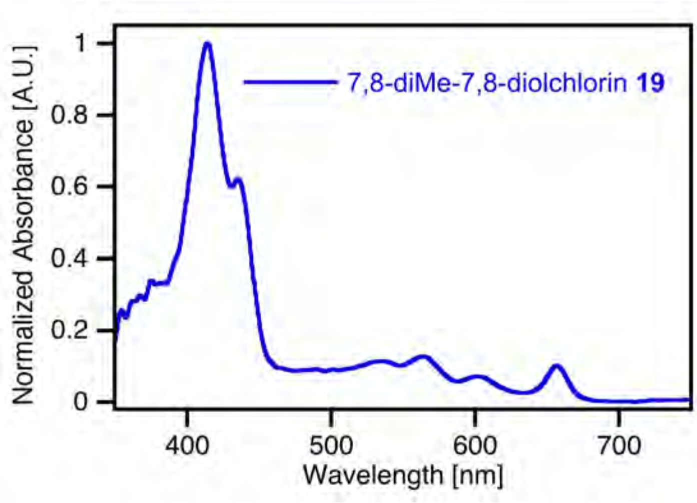

Figure S-3. UV-vis spectrum (CH_2Cl_2) of *meso*-tetrahexyl-7,8-dihydroxy-7,8-dimethyl-chlorin (**19**).

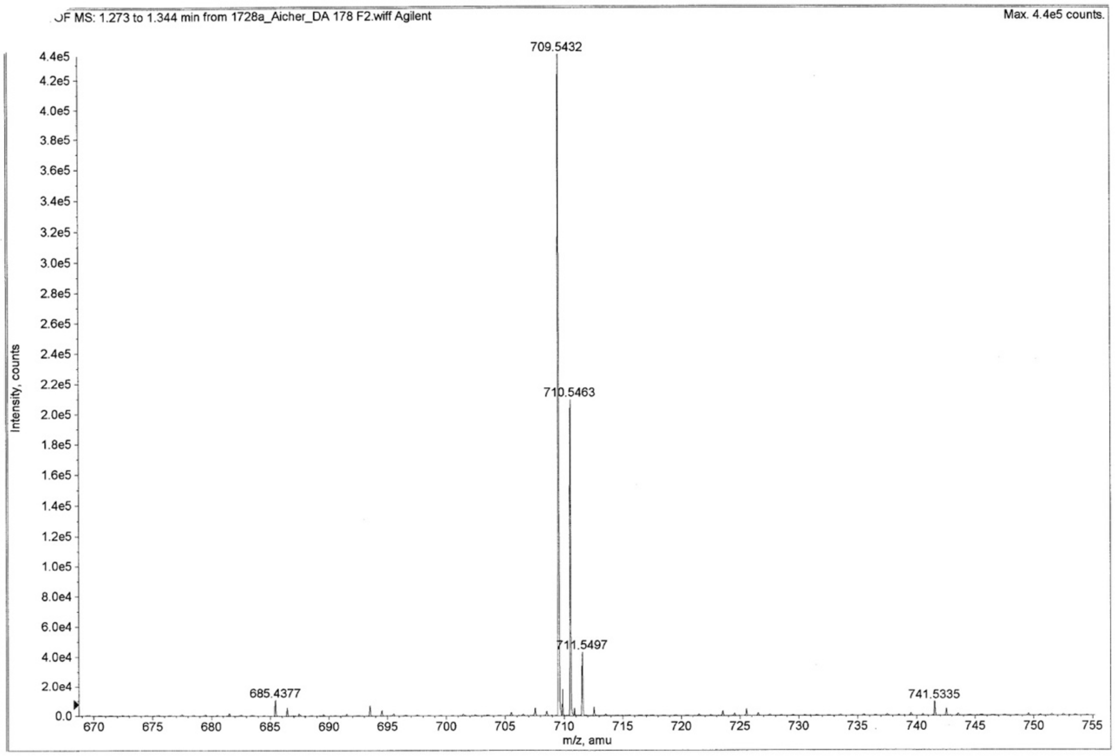

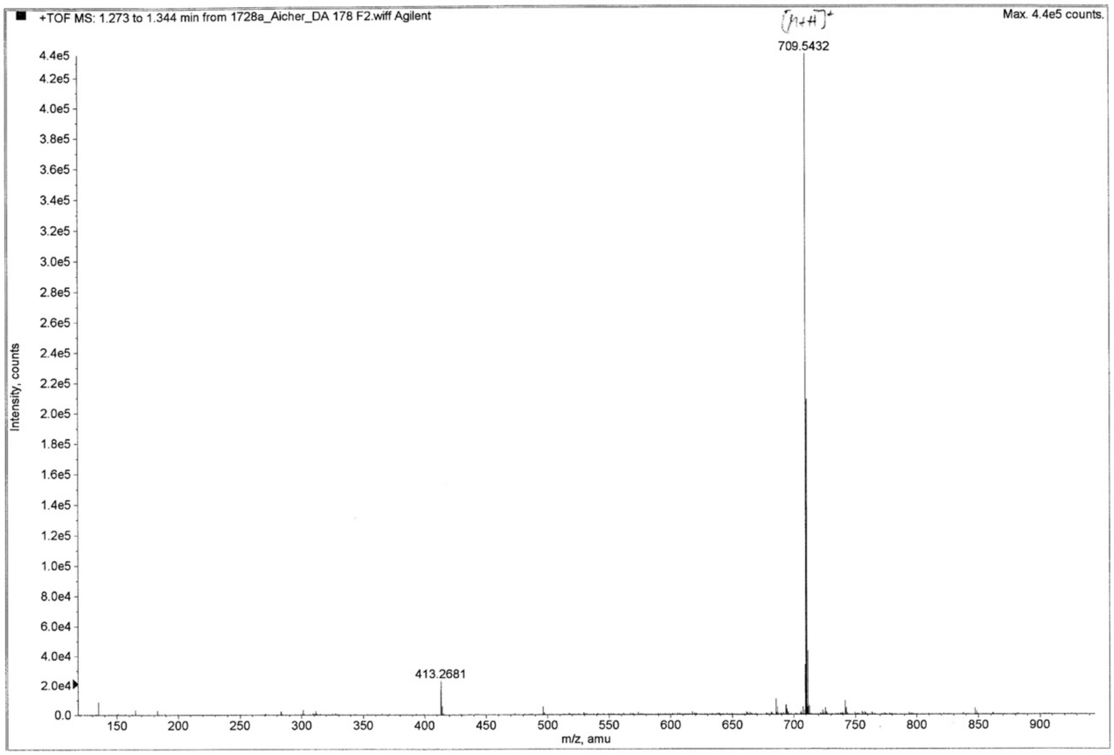

Figure S-4. Mass spec of *meso*-tetrahexyl-7,8-dihydroxy-7,8-dimethyl-chlorin (**19**).

meso-Tetrahexyl-8-hydroxy-8-trifluoromethyl-chlorin-7-one (18^F).

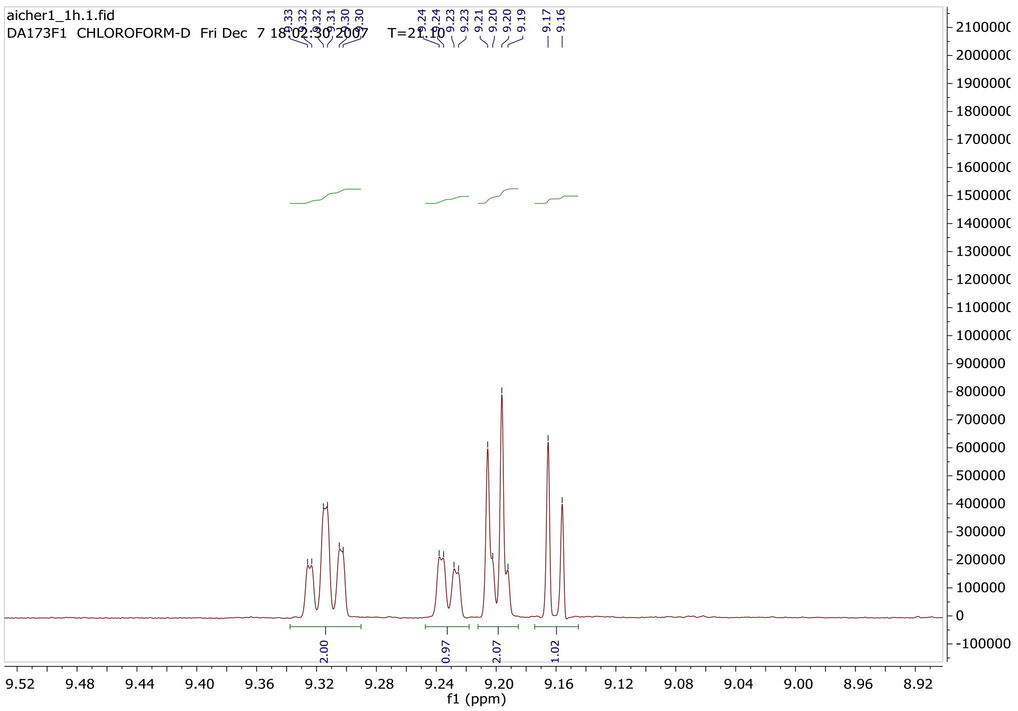

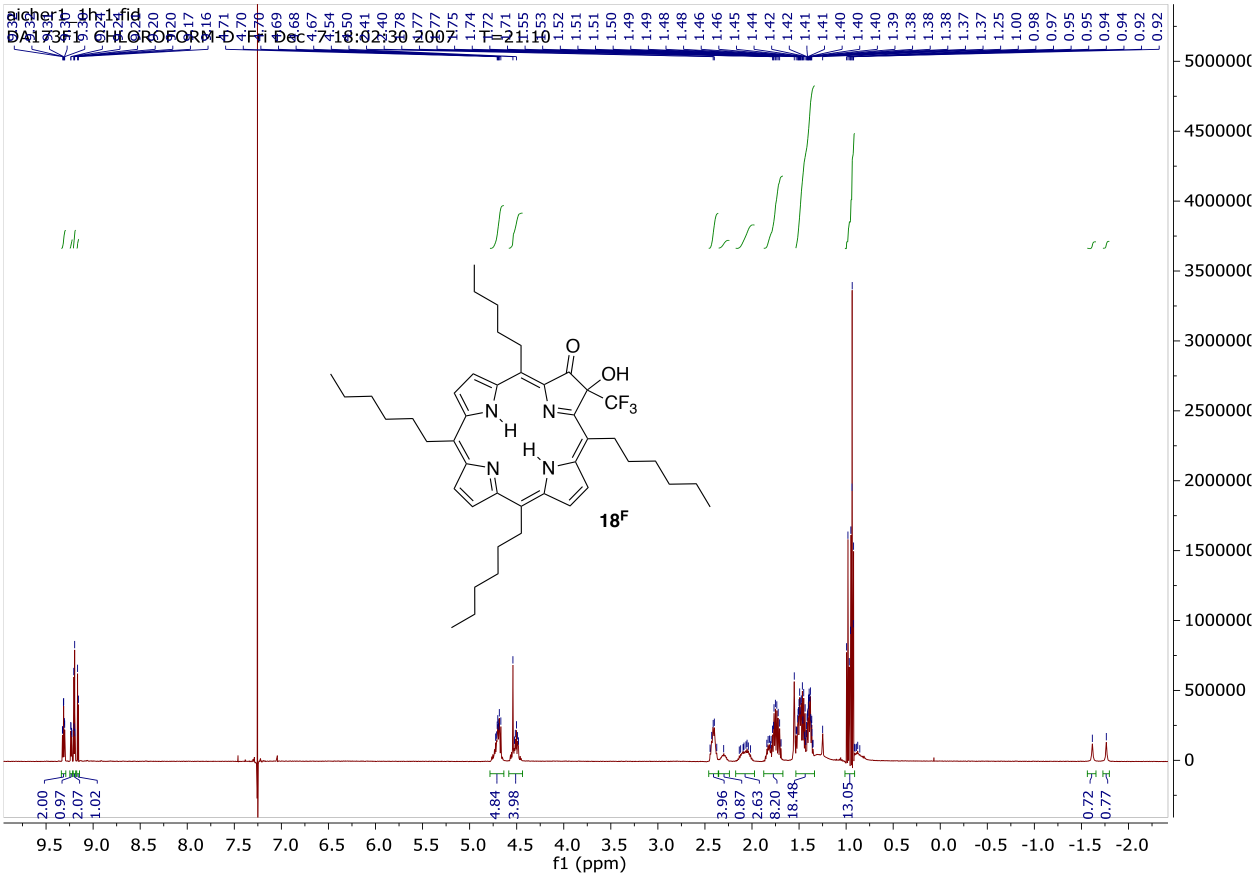

Figure S-1. ^{1}H NMR (500 MHz, $CDCl_3$) spectrum of *meso*-tetrahexyl-8-hydroxy-8-trifluoromethyl-chlorin-7-one (**18^F**).

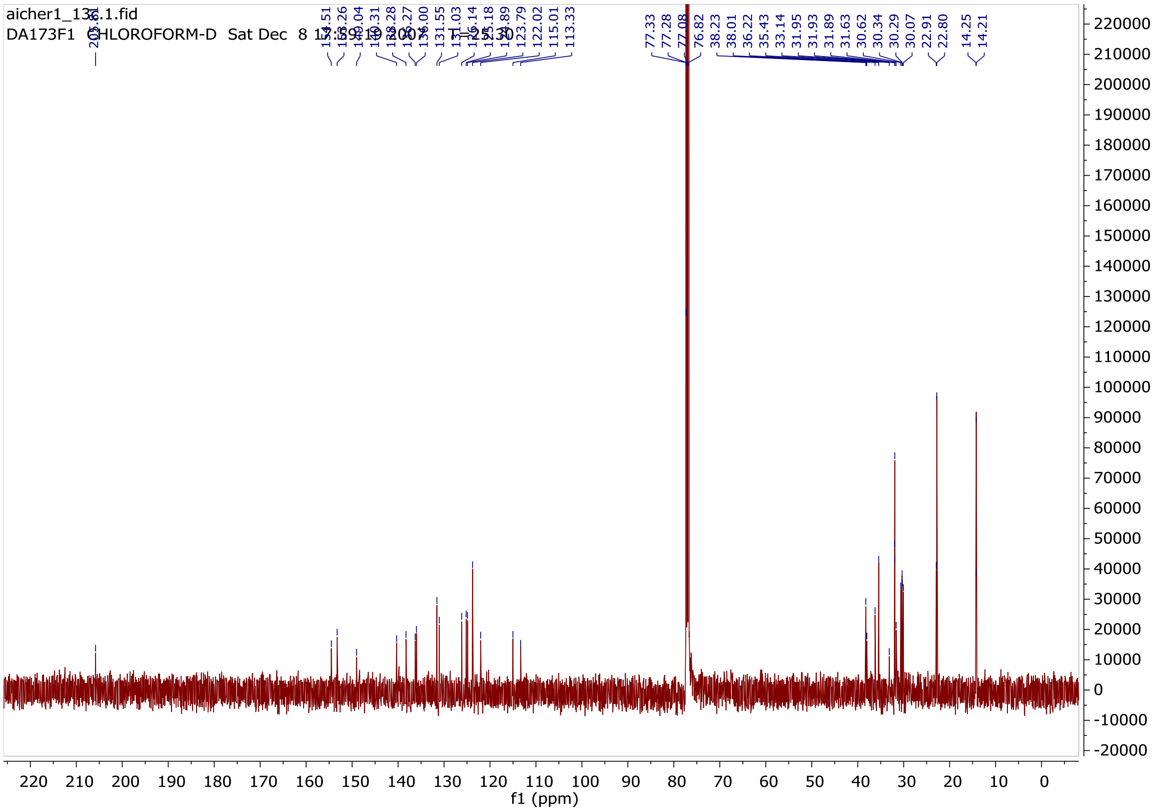

Figure S-2. 13C NMR of *meso*-tetrahexyl-8-hydroxy-8-trifluoromethyl-chlorin-7-one (**18**^{F}).

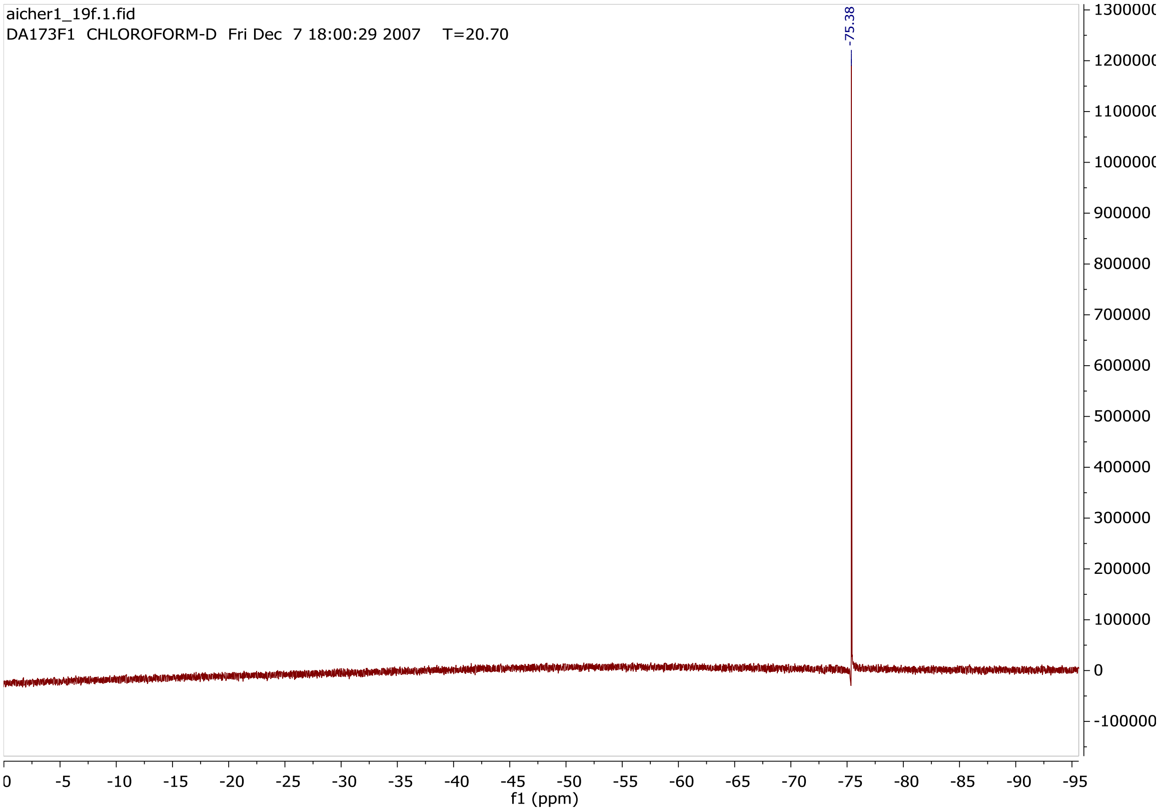

Figure S-3. 19F NMR (126 MHz, $CDCl_3$) of *meso*-tetrahexyl-8-hydroxy-8-trifluoromethyl-chlorin-7-one (**18**^{F}).

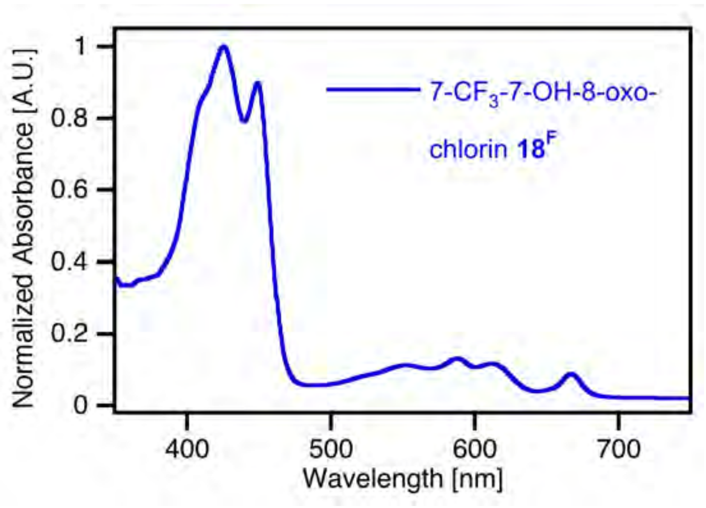

Figure S-4. UV-vis spectrum (CH_2Cl_2) of *meso*-tetrahexyl-8-hydroxy-8-trifluoromethyl-chlorin-7-one (**18**^{F}).

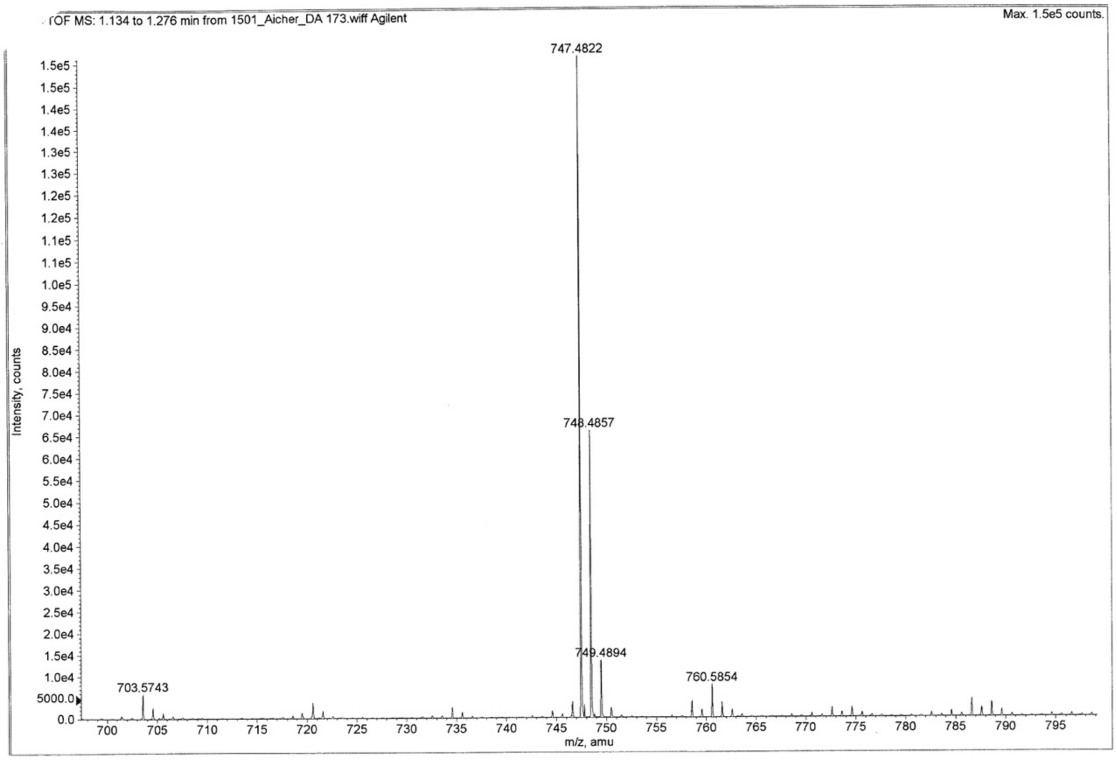

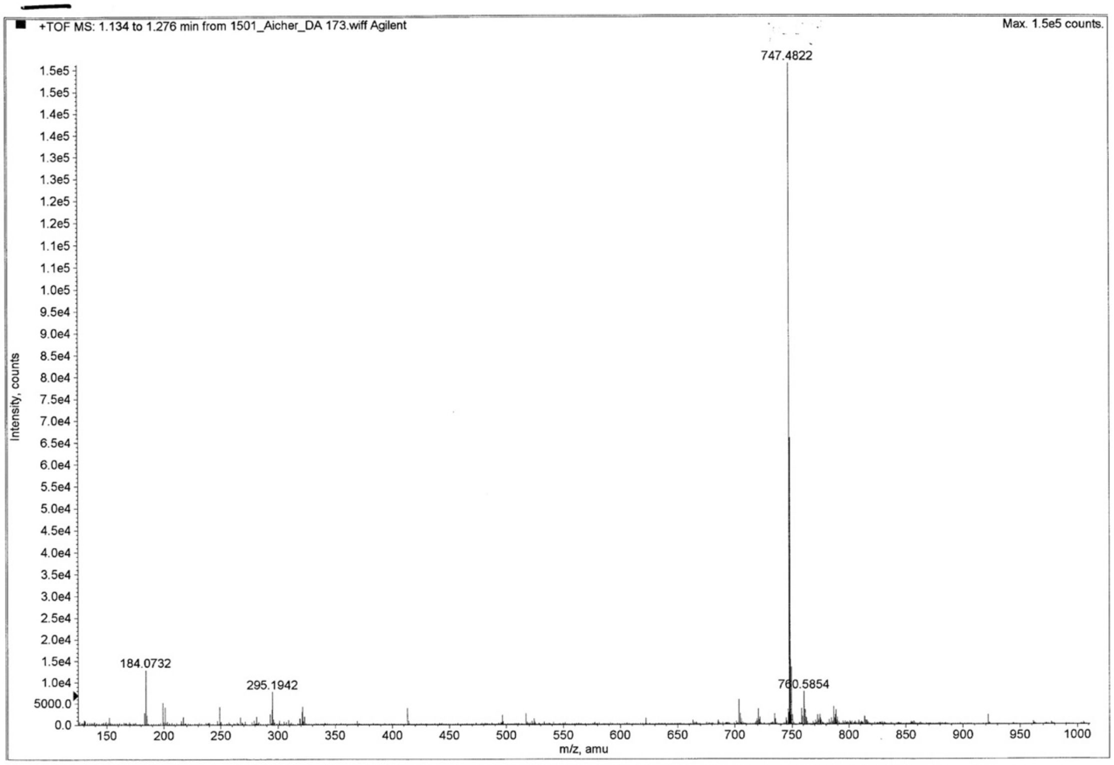

Figure S-5. Mass spec of *meso*-tetrahexyl-8-hydroxy-8-trifluoromethyl-chlorin-7-one (**18^F**).

meso-Tetrahexyl-7,8-dihydroxy-8-methyl-chlorin (20).

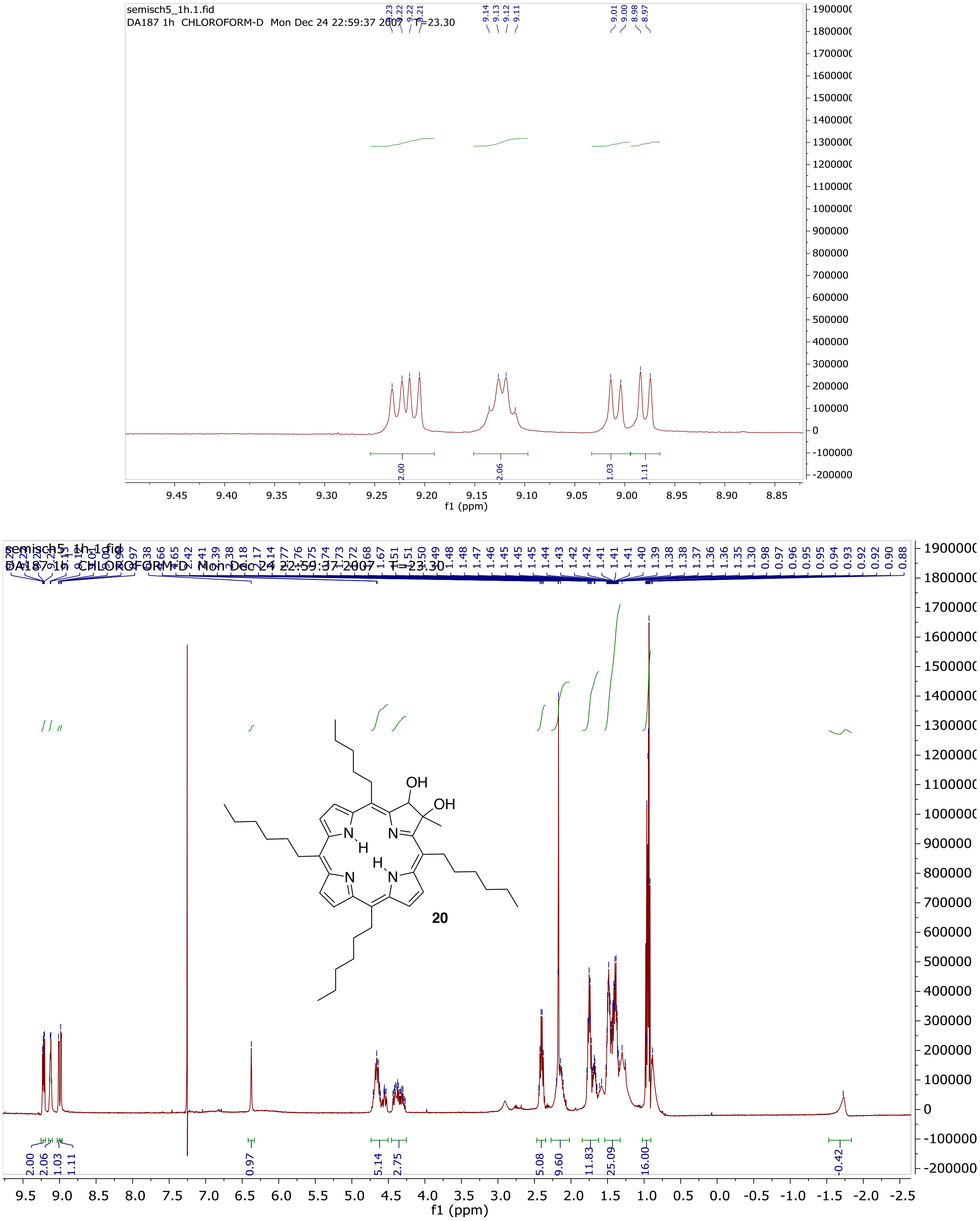

Figure S-1. ^{1}H NMR (500 MHz, $CDCl_3$) spectrum of *meso*-tetrahexyl-7,8-dihydroxy-8-methyl-chlorin (**20**).

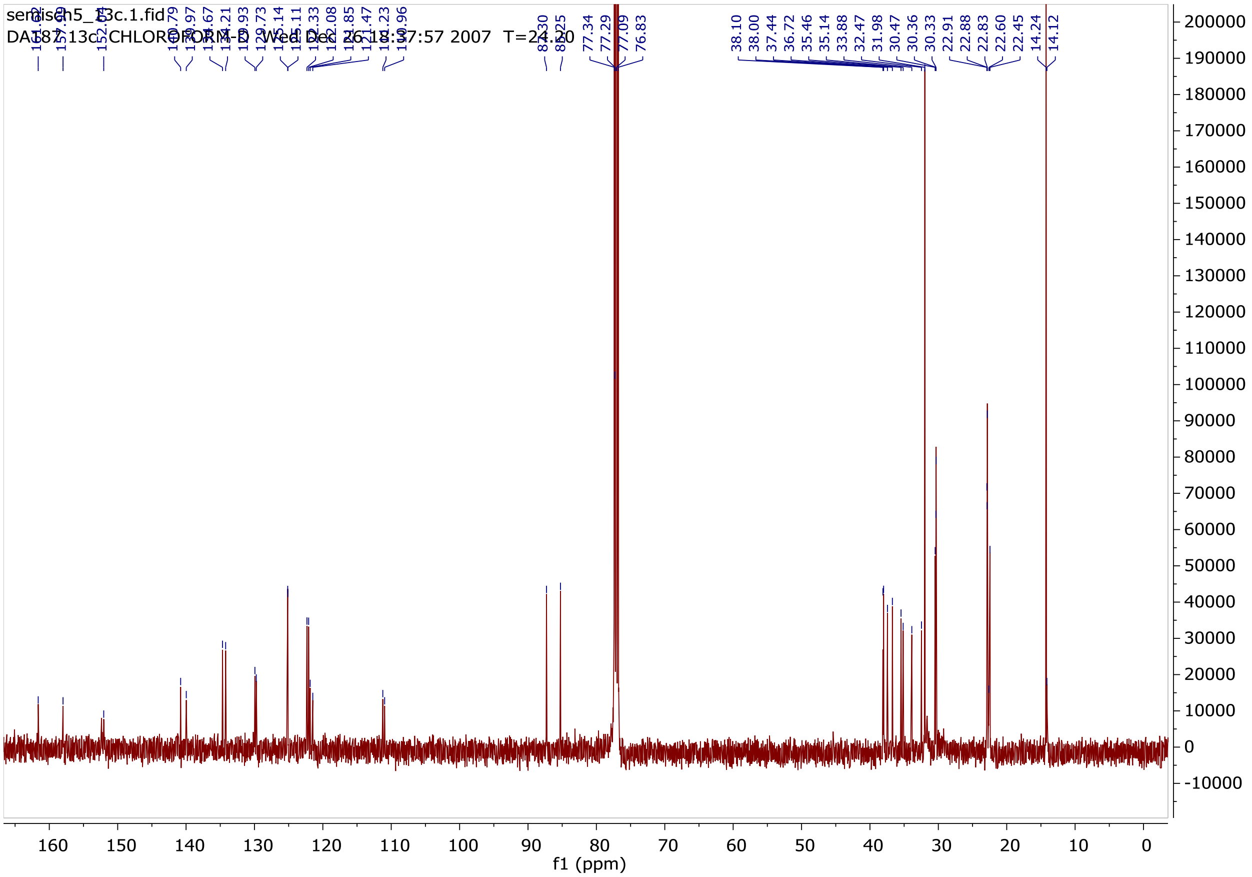

Figure S-2. 13C NMR (126 MHz, $CDCl_3$) of *meso*-tetrahexyl-7,8-dihydroxy-8-methyl-chlorin (**20**).

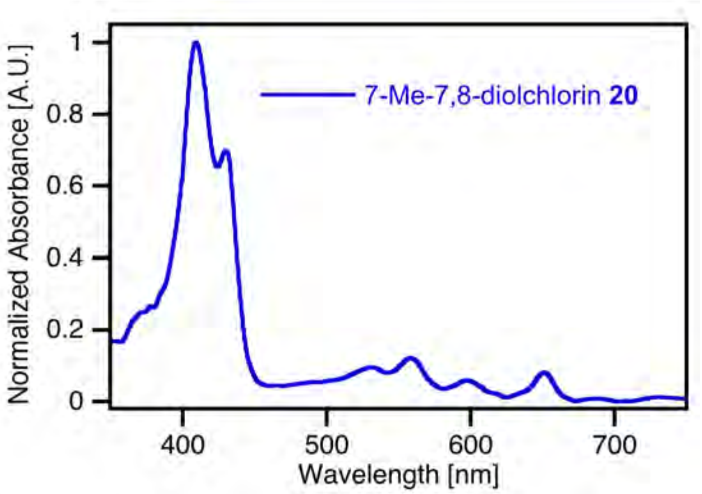

Figure S-3. UV-vis spectrum (CH_2Cl_2) of *meso*-tetrahexyl-7,8-dihydroxy-8-methyl-chlorin (**20**).

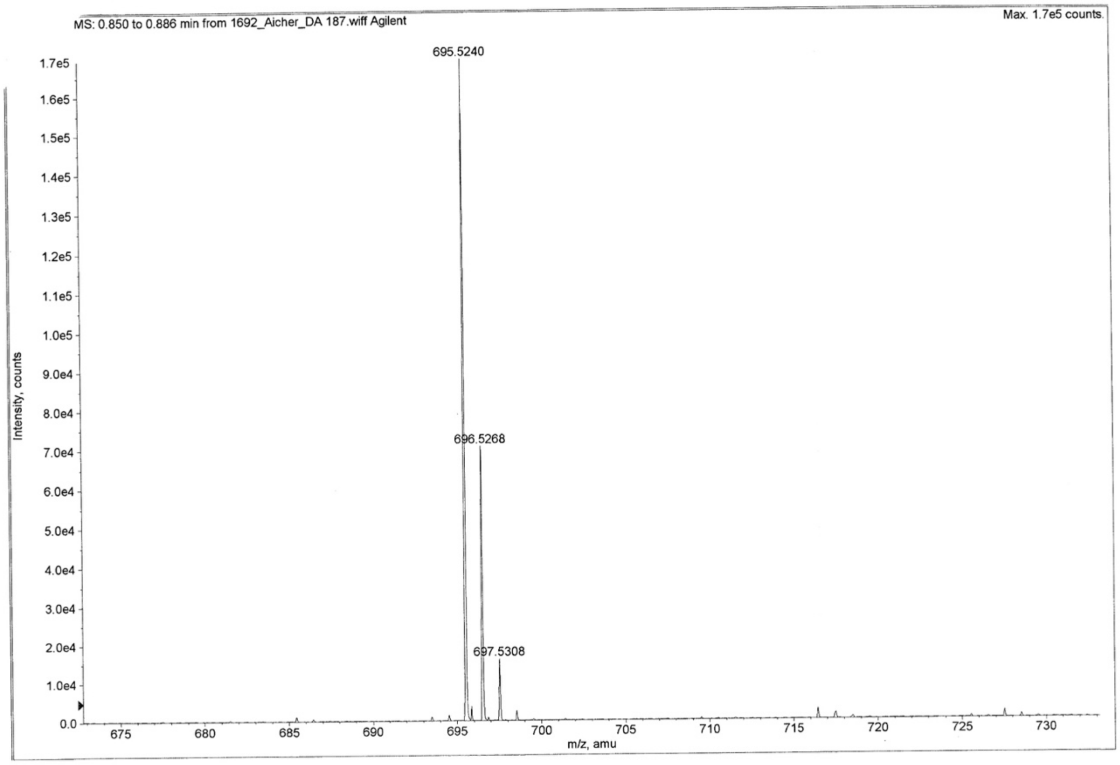

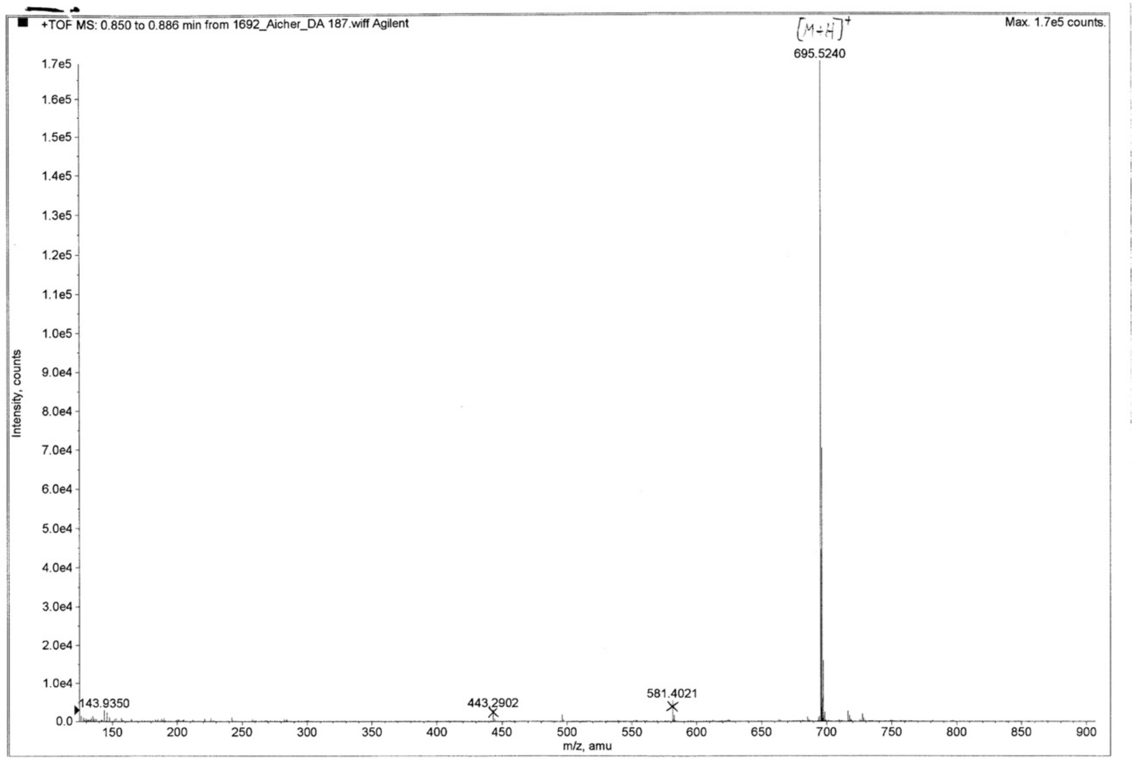

Figure S-4. Mass spec of *meso*-tetrahexyl-7,8-dihydroxy-8-methyl-chlorin (**20**).

meso-Tetrahexyl-7,8-dihydroxy-8-trifluoromethyl-chlorin (20^F).

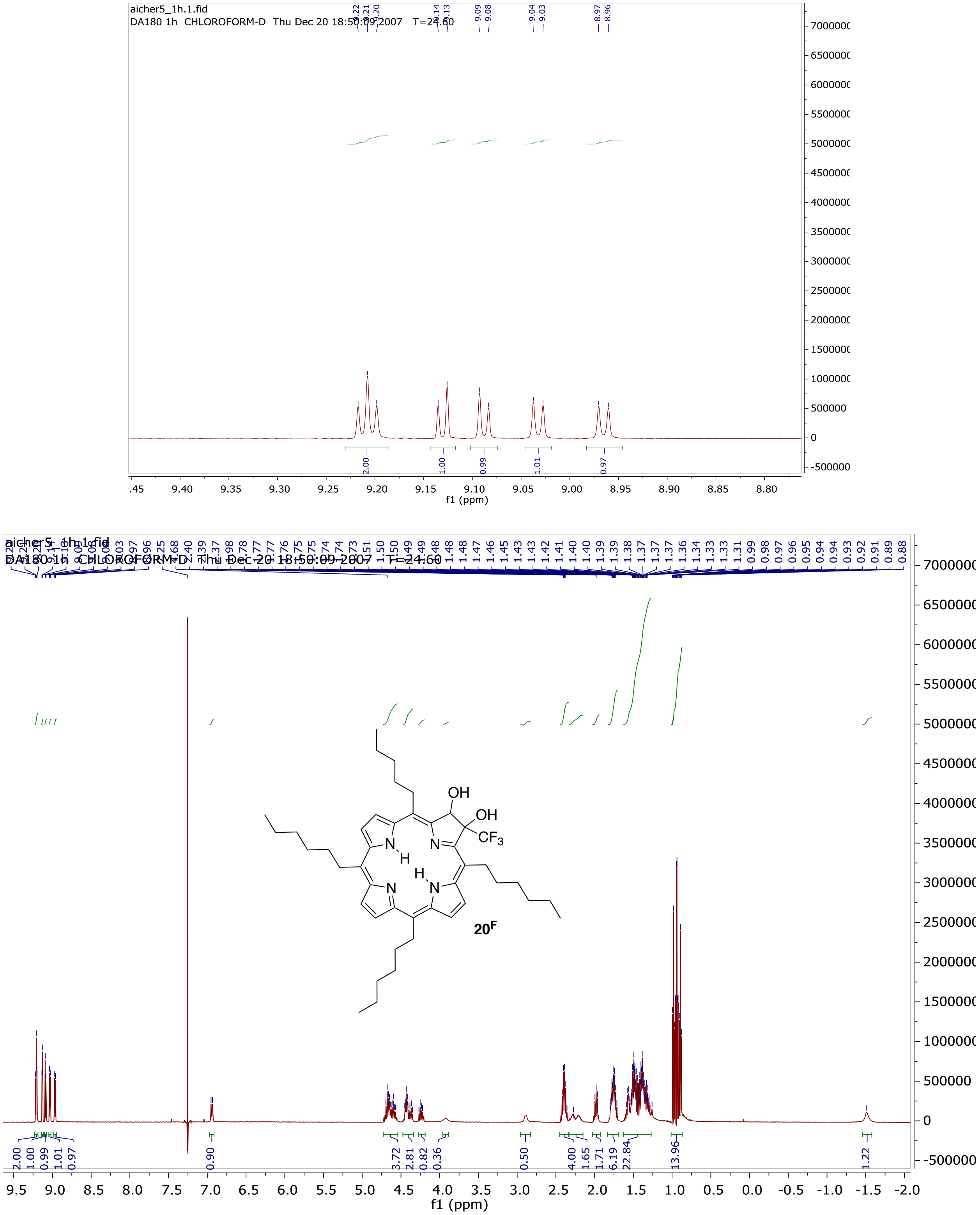

Figure S-1. ^1H NMR (500 MHz, $CDCl_3$) spectrum of *meso*-tetrahexyl-7,8-dihydroxy-8-trifluoromethyl-chlorin (**20^F**) (full and detail).

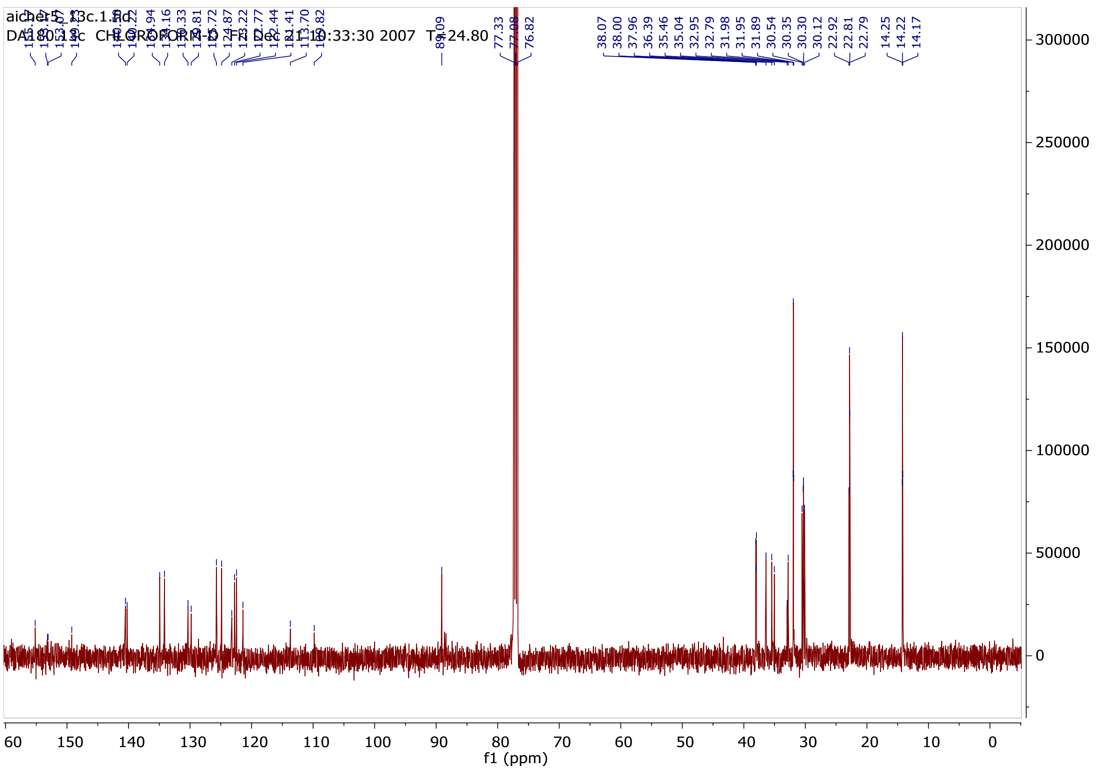

Figure S-2. 13C NMR (126 MHz, $CDCl_3$) of *meso*-tetrahexyl-7,8-dihydroxy-8-trifluoromethyl-chlorin (**20**[F]).

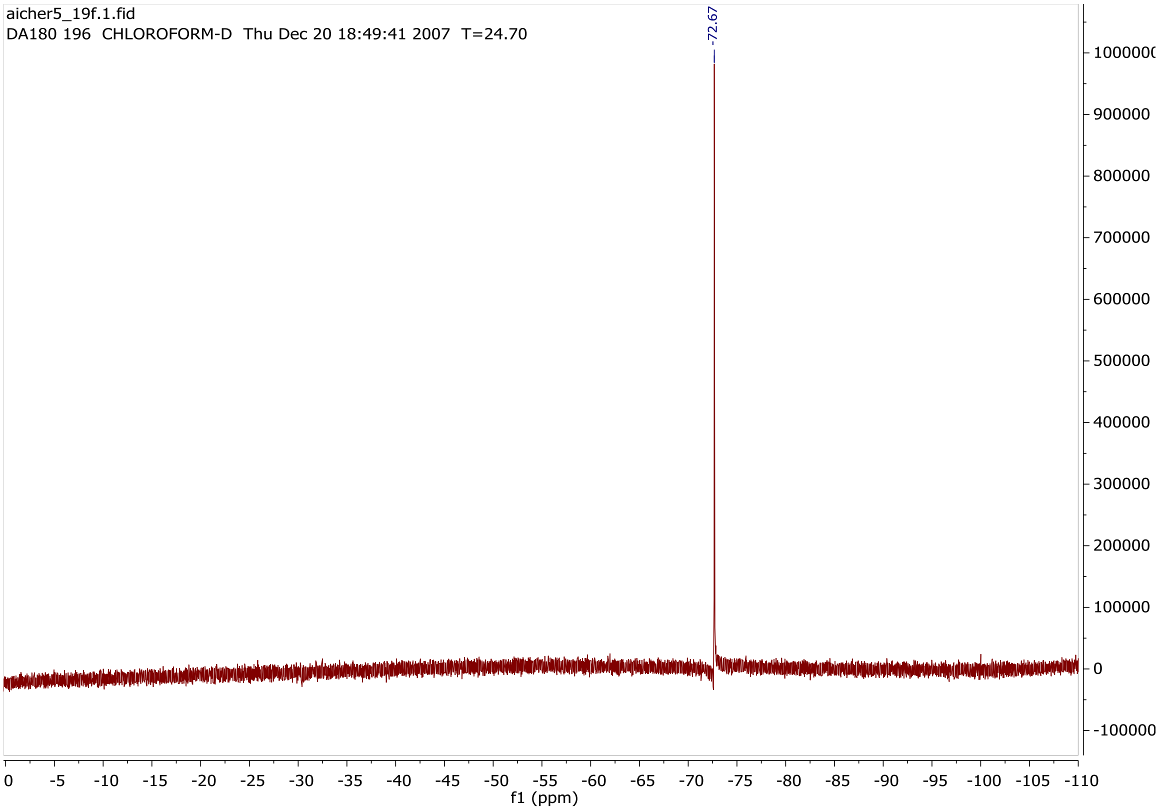

Figure S-3. 19F NMR of *meso*-tetrahexyl-7,8-dihydroxy-8-trifluoromethyl-chlorin (**20**[F]).

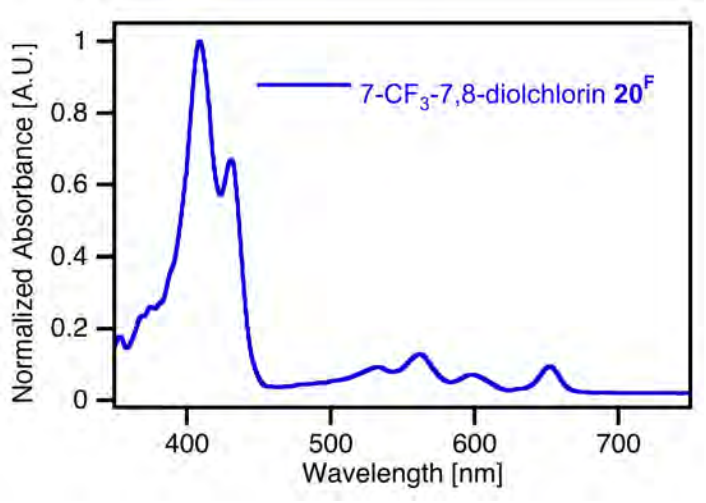

Figure S-4. UV-vis spectrum (CH_2Cl_2) of *meso*-tetrahexyl-7,8-dihydroxy-8-trifluoromethyl-chlorin (**20**^{F}).

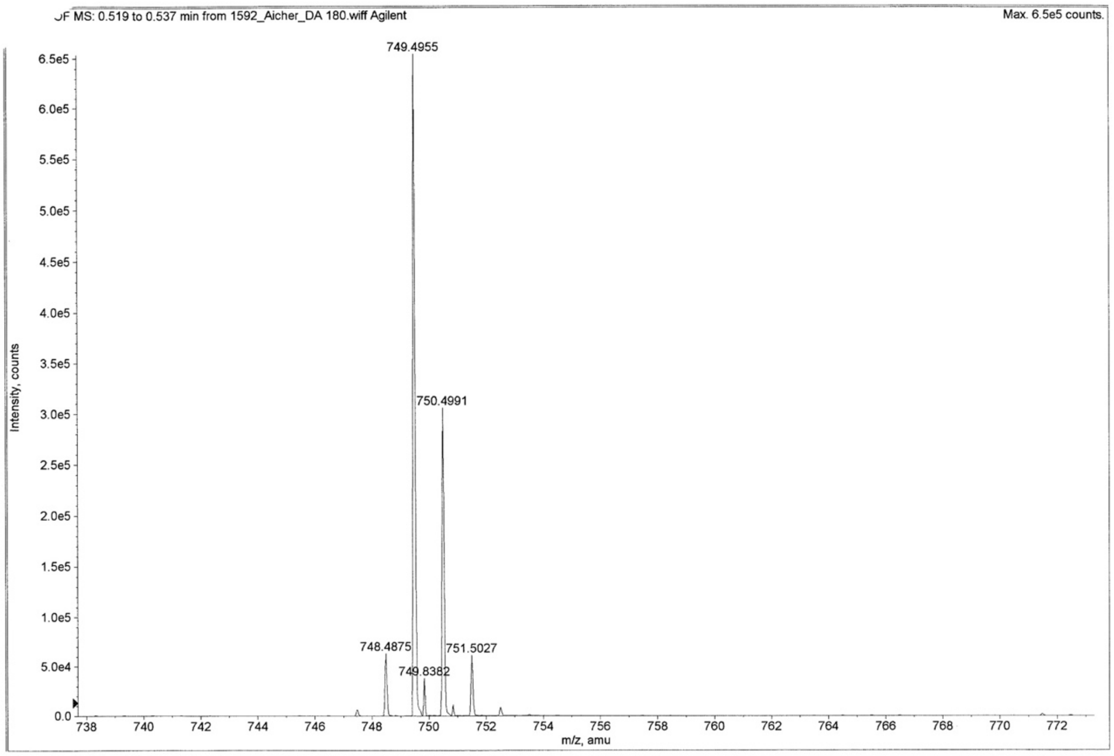

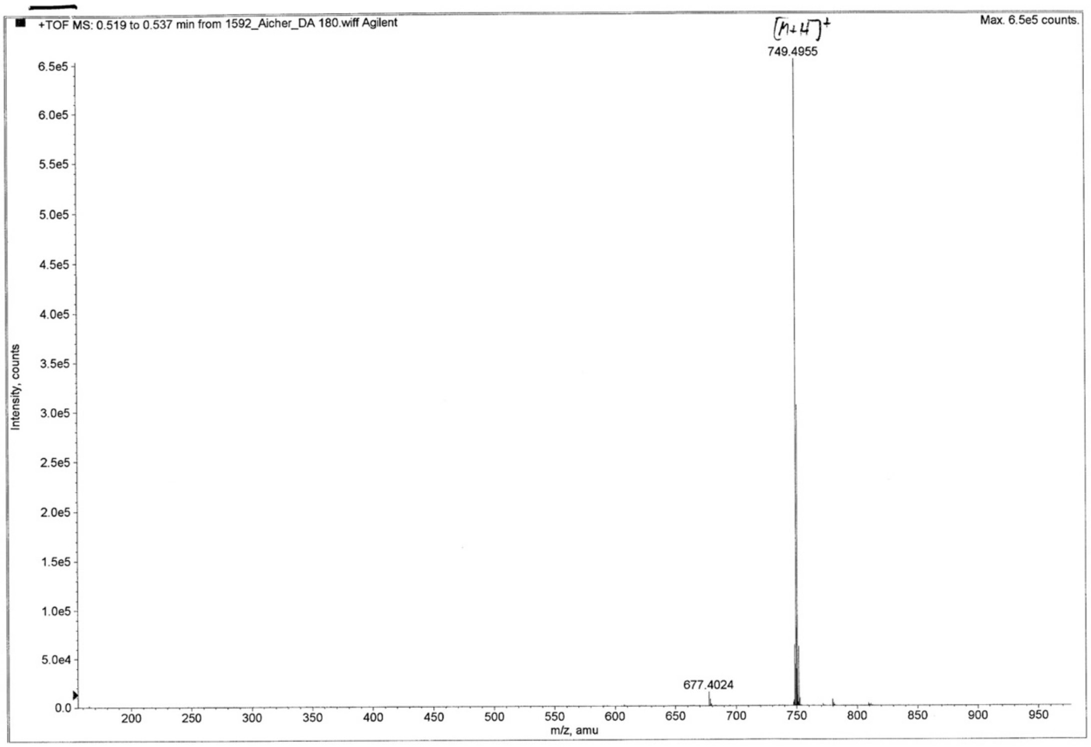

Figure S-5. HR-MS (ESI+, TOF) of *meso*-tetrahexyl-7,8-dihydroxy-8-trifluoromethyl-chlorin (**20**^{F}) (full and detail).
